# A Na^+^ leak channel cloned from *Trichoplax adhaerens* extends extracellular pH and Ca^2+^ sensing for the DEG/ENaC family close to the base of Metazoa

**DOI:** 10.1074/jbc.RA119.010542

**Published:** 2019-09-15

**Authors:** Wassim Elkhatib, Carolyn L. Smith, Adriano Senatore

**Affiliations:** ‡ Department of Biology, University of Toronto Mississauga, Mississauga, Ontario L5L 1C6, Canada; § NINDS, National Institutes of Health, Bethesda, Maryland 20892

**Keywords:** patch clamp, invertebrate, evolution, sodium channel, acid-sensing ion channel (ASIC), calcium, molecular evolution, amiloride, Ca2+ block, DEG/ENaC sodium channels, proton block, Trichoplax adhaerens

## Abstract

Acid-sensitive ion channels belonging to the degenerin/epithelial sodium channel (DEG/ENaC) family activate in response to extracellular protons and are considered unique to deuterostomes. However, sensitivity to pH/protons is more widespread, where, for example, human ENaC Na^+^ leak channels are potentiated and mouse BASIC and *Caenorhabditis elegans* ACD-1 Na^+^ leak channels are blocked by extracellular protons. For many DEG/ENaC channels, extracellular Ca^2+^ ions modulate gating, and in some cases, the binding of protons and Ca^2+^ is interdependent. Here, we functionally characterize a DEG/ENaC channel from the early-diverging animal *Trichoplax adhaerens*, *Tad*NaC6, that conducts Na^+^-selective leak currents *in vitro* sensitive to blockade by both extracellular protons and Ca^2+^. We determine that proton block is enhanced in low external Ca^2+^ concentration, whereas calcium block is enhanced in low external proton concentration, indicative of competitive binding of these two ligands to extracellular sites of the channel protein. *Tad*NaC6 lacks most determinant residues for proton and Ca^2+^ sensitivity in other DEG/ENaC channels, and a mutation of one conserved residue (S353A) associated with Ca^2+^ block in rodent BASIC channels instead affected proton sensitivity, all indicative of independent evolution of H^+^ and Ca^2+^ sensitivity. Strikingly, *Tad*NaC6 was potently activated by the general DEG/ENaC channel blocker amiloride, a rare feature only reported for the acid-activated channel ASIC3. The sequence and structural divergence of *Tad*NaC6, coupled with its noncanonical functional features, provide unique opportunities for probing the proton, Ca^2+^, and amiloride regulation of DEG/ENaC channels and insight into the possible core-gating features of ancestral ion channels.

## Introduction

DEG/ENaC^2^ ion channels comprise a large family of animal-specific Na^+^ channels that operate either as constitutively open Na^+^ leak channels or as transiently open channels that are activated and/or modulated by distinct but sometimes overlapping stimuli. For example, epithelial Na^+^ leak channels (*i.e.* ENaC channels), important for Na^+^ re-uptake and homeostasis, are constitutively open but modulated by extracellular protons, peptide hormones, bile acids, proteolytic cleavage, and mechanical stress (1–3). The vertebrate bile acid–sensitive channels (*i.e.* BASIC or ASIC5 channels) conduct leak Na^+^ currents that are enhanced by bile acids and blocked by external Ca^2+^ ions (4, 5). Acid-sensitive ion channels (ASICs), important for extracellular pH sensing, are potently and transiently activated by extracellular acidification/protons, but are dynamically modulated by extracellular Ca^2+^, mechanical stress, proteolytic cleavage, and neuropeptides (1, 6, 7). The peptide-gated FaNaC channels from gastropod molluscs, which are directly activated by the ligand neuropeptide FMRF-amide (8), are also inhibited by protons and Ca^2+^ (9). In *Caenorhabditis elegans*, the mechanosensory degenerin (DEG) and MEC channels are activated by mechanical stimuli (10), whereas ACD-1 acts as a Na^+^ leak channel that is blocked by external protons and Ca^2+^ (11). Indeed, the extensive diversity in DEG/ENaC channel gating, coupled with their independent duplication in distinct animal lineages and incomplete lineage sorting apparent on phylogenetic trees (12–14), makes it difficult to conceptualize what the core functional attributes of the primordial DEG/ENaC channel might have been like. To date, the most early-diverging DEG/ENaC channels to be functionally characterized are from the cnidarian *Hydra magnipapillata*, where they were found to be similar to FaNaC channels in their activation by neuropeptides (*Hydra*–RF-amides) and blocked by external Ca^2+^ (13) but, in contrast, insensitive to protons (14). Notably, DEG/ENaC channels are present in the genomes and transcriptomes of the three most early diverging animal phyla: Placozoa, Porifera, and Ctenophora (12), but their functional features have not yet been explored. Thus, studies on DEG/ENaC homologues from these early diverging animals will help develop a better understanding about the core characteristics and evolution of this large family of ion channels.

Among these, placozoans such as *Trichoplax adhaerens* are particularly interesting in that, despite lacking synapses and a nervous system, they express a nearly complete set of genes involved in electrical neural signaling (15), including most major families and classes of ion channels. Furthermore, *Trichoplax* is able to conduct motile behaviors, including feeding, chemotaxis, and geotaxis (16–18), employing secreted neuropeptides for roles in coordinating cellular activity (19, 20). Notably, with the exception of the voltage-gated T-type calcium channel (21), the functional properties of *Trichoplax* electrogenic genes have yet to be reported. Here, we report the cloning and functional expression of a second *Trichoplax* ion channel from the DEG/ENaC family, named *Tad*NaC6 for *Trichoplax adhaerens* Na^+^ channel subtype 6.

Phylogenetically, all but one of the *Trichoplax* DEG/ENaC channels form a sister clade with deuterostome pH-sensitive ASIC channels, whereas the homologues from protostome invertebrates such as arthropods, molluscs, and nematodes form separate clades. *In situ* hybridization of *Tad*NaC6 revealed expression within rows of cells that line the periphery on the animal, a region bearing cells thought to be involved in cellular communication (20, 22). *In vitro* expression of *Tad*NaC6 in Chinese hamster ovary (CHO) cells, coupled with whole-cell patch-clamp recording, revealed robust Na^+^-selective leak currents that were blocked by both external protons and Ca^2+^, a functional profile also reported for the mouse BASIC and nematode ACD-1 DEG/ENaC channels (5, 11). Proton block of *Tad*NaC6 was biphasic in the presence of 2 mm external Ca^2+^, but monophasic in 0.1 mm Ca^2+^, revealing an interplay between proton and Ca^2+^ block. In accordance, proton block was enhanced in low Ca^2+^, while Ca^2+^ block was enhanced in low [H^+^], indicative of competition for channel block by these two extracellular ligands. Notably, an S353A mutation, within a homologous site shown to be a key determinant for differential Ca^2+^ sensitivity in rodent BASIC channels (5), had no effect on Ca^2+^ block for *Tad*NaC6, but instead, it slightly reduced its proton sensitivity. Indeed, given its phylogenetic distance from the functionally similar mouse BASIC and nematode ACD-1 Na^+^ leak channels, and the absence of key amino acids involved in proton and Ca^2+^ sensitivity in other DEG/ENaC channels, it is likely that the gating features of *Tad*NaC6 evolved independently. Nevertheless, our work extends extracellular protons and Ca^2+^ as modulatory ligands for DEG/ENaC channels close to the base of Metazoa. Finally, *Tad*NaC6 was found to be activated by the general DEG/ENaC channel blocker amiloride, a rare feature only reported for rat ASIC3 (23) and a mutant version of human ASIC2 (24), but lacks key residues attributed to this phenomenon. Instead, diminazene, another general DEG/ENaC channel blocker, produced a low-affinity block corroborating that the two drugs have fundamentally different modes of action. The highly-divergent sequence and atypical properties observed for *Tad*NaC6 provide some unique perspectives on the structure, function, and evolution of proton- and Ca^2+^-sensitive DEG/ENaC channels.

## Results

### T. adhaerens DEG/ENaC ion channels phylogenetically cluster with vertebrate ASIC channels and exhibit distinct spatial expression patterns

**Figure 1.**
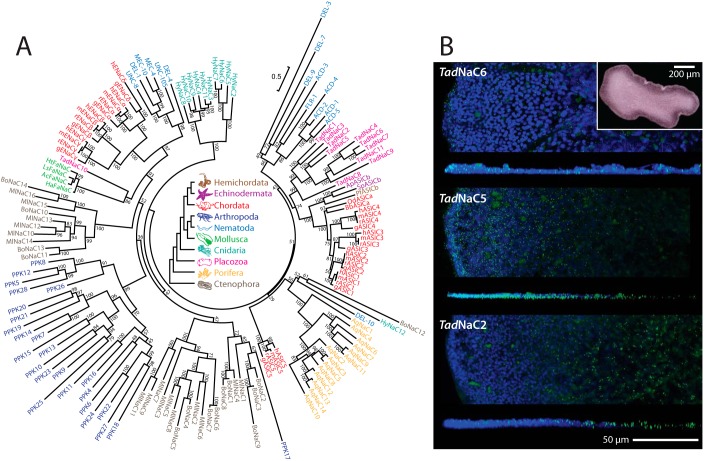
*A,* maximum likelihood phylogeny of DEG/ENaC ion channels from various animals inferred from a MUSCLE protein alignment with IQTree. Ultrafast bootstrap support values are indicated, and ion channel names are colored according to the respective phylum as indicated by the *inset* within the center of the phylogenetic tree. *B,* fluorescence *in situ* hybridization with probes for *Tad*NaC6, *Tad*NaC5, and *Tad*NaC2 in whole mounts of *Trichoplax*. For each probe (*green grains*), the *upper image* shows a horizontal projection of optical sections through a region extending halfway across the animal, and the *lower image* shows a vertical projection of a 12-μm–wide strip through the same region. Larger *pale green objects* are autofluorescent cellular inclusions. Nuclei are *blue. Scale,* 50 μm. The *inset on the top right* is a photograph of a *T. adhaerens* specimen in the same orientation as stained animals imaged using fluorescence imaging. *Scale,* 200 μm.

Previously, we reported the cloning of complete open reading frames for nine DEG/ENaC ion channel homologues identified in the transcriptome of the early-diverging animal *T. adhaerens*, while two others identified in the genome failed to amplify via RT-PCR from whole-animal total RNA.^3^ A maximum likelihood phylogenetic tree of the *Trichoplax* channels, along with DEG/ENaC channel proteins from various representative animals, reveals that all but one of the 11 *Trichoplax* homologues form a sister clade with ASIC channels from vertebrates, whereas the BASIC channels (also known as ASIC5 or BLiNAC channels) form a separate node from these two groups (Fig. 1*A*). Similar to the nomenclature used to name the cnidarian (*Hydra*) DEG/ENaC Na^+^ channels (*i.e.* HyNaCs), we chose to refer to the *Trichoplax* channels as *Tad*NaCs 1–11. Notably, no protostome invertebrate DEG/ENaC channels clustered between the *Trichoplax* and vertebrate ASIC clades, and generally, the phylogenetic positions of the various ion channels throughout the tree do not correspond with the metazoan phylogeny (Fig. 1*A*, inset). Whether this extensive incomplete lineage sorting is attributable to sequence divergence/convergence, or extensive gene gain/loss, is difficult to discern. However, it is notable that clades with strong bootstrap support tend to be populated by channels from a single species or phylum, consistent with proposed lineage-specific genetic expansion events for DEG/ENaC channels within the Metazoa (12). One exception is a node/clade that includes a *Tad*NaC10, the FMRF-amide peptide-activated channels from gastropods (*i.e.* FaNaCs) (25–27), the vertebrate ENaCs (28), and the nematode mechanically-gated DEG/UNC/MEC/DEL channels (29). The gating modalities of this particular clade of channels are relatively well-understood and quite distinct from each other, underscoring that even closely-related channels at the protein sequence level can exhibit divergent functional profiles. The phylogenetic tree also depicts 14 DEG/ENaC ion channel homologues identified in an updated gene dataset for the sponge *Amphimedon queenslandica* (Fig. 1*A*), notable because these were previously reported absent (14). Finally, at least two distinct clades of ctenophore DEG/ENaC channels are apparent on the tree, none sharing strongly supported nodes with clades from other animals. Altogether, the DEG/ENaC ion channel family appears to have undergone extensive lineage-specific evolutionary change, a feature similarly observed for other gene families involved in sensory functions (30).

A recent single cell transcriptome study on *T. adhaerens* revealed that the DEG/ENaC channels *Tad*NaC2 (TRIADDRAFT_58143), *Tad*NaC3 (TRIADDRAFT_58141), and *Tad*NaC8 (TRIADDRAFT_29260) are co-expressed in lipophil cells, which bear large acidophilic vesicles proposed to be secreted along the ventral epithelium for external digestion during feeding (22). A single channel, *Tad*NaC10 was found to be expressed in ciliated epithelial cells, possibly the ventral cells involved in ciliary locomotion (31). Here, fluorescence i*n situ* hybridization of *Tad*NaC6, *Tad*NaC5, and *Tad*NaC2 revealed somewhat overlapping cellular expression, with *Tad*NaC6 label being most prevalent in a narrow band at the edge, whereas the *Tad*NaC5 label is abundant in a region extending ∼100 μm from the edge. *Tad*NaC2 label is very sparse within 100 μm of the edge but more prevalent farther in the interior (Fig. 1*B*), the zone occupied by lipophil cells.

### TadNaC6 is a proton-inhibited cation leak channel in vitro, similar to vertebrate BASIC and nematode ACD-1 channels

**Figure 2.**
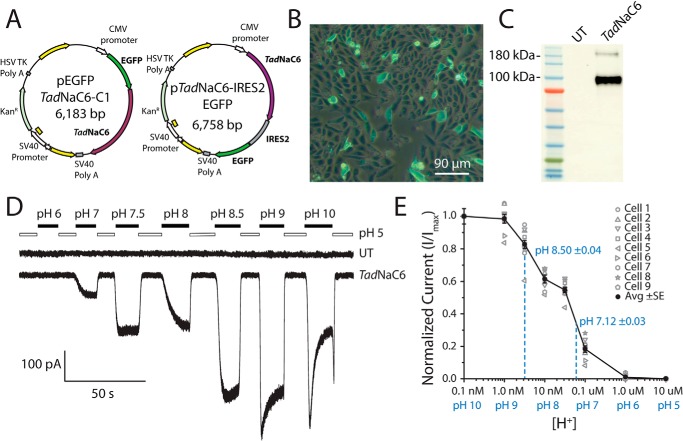
*A,* illustration of plasmid vectors used to heterologously express the *Tad*NaC6 channel in CHO cells. The pEGFP–*Tad*NaC6–C1 fusion expresses an N-terminally GFP-tagged protein *in vitro*, and p*Tad*NaC6–IRES2–EGFP expresses untagged *Tad*NaC6 co-expressed with GFP through an internal ribosome entry site. *B,* overlaid fluorescence and transmitted light images of CHO cells transfected with the pEGFP–*Tad*NaC6–C1 plasmid revealing efficient expression of the GFP-tagged *Tad*NaC6 channels *in vitro. C,* Western blotting of protein lysates from CHO cells transfected with pEGFP–*Tad*NaC6–C1 with anti-GFP polyclonal antibodies confirms expression of the full-length GFP–*Tad*NaC6 fusion protein. *D,* untagged *Tad*NaC6 produced robust cation leak currents in CHO cells recorded via whole-cell voltage clamp. Untransfected cells (*UT*) produced no leak currents when membrane voltage was held at −60 mV, whereas *Tad*NaC6-transfected cells produced inward currents that were blocked by acidic pH and approached a maximum amplitude at pH values above 9. *E,* dose-response curve of proton block for *Tad*NaC6 reveals a biphasic attenuation of normalized maximal inward current with decreasing pH values, with a high-affinity block IC_50_ of pH 8.5 ± 0.04 and a lower affinity block IC_50_ of 7.12 ± 0.03. Current amplitudes for individual cells are shown in *gray*, and the average current values plus/minus standard error in *black.*

We subcloned all nine cloned *Trichoplax* DEG/ENaC channel open reading frames into the mammalian expression vectors pIRES2-EGFP and pEGFP-C1 (Fig. 2*A*), in order to attempt functional expression for *in vitro* electrophysiology. Although various expressed *Trichoplax* channels have produced functional currents *in vitro*, this study focuses solely on *Tad*NaC6, which possesses some striking and unique functional attributes. The vector pEGFP–TadNaC6 permits ectopic expression of the channel with an N-terminal fusion tag of the green fluorescent protein (GFP), which was used to visualize successful channel expression in CHO cells via fluorescence microscopy (Fig. 2*B*), and as a full-length channel protein fused to GFP on Western blottings using polyclonal anti-GFP antibodies (Fig. 2*C*). Also evident is a fainter band on the blot with a molecular mass of roughly 200 kDa, absent in untransfected cell lysates, which possibly represents dimerized EGFP–TadNaC6 fusion proteins. Expression of the unfused *Tad*NaC6 cDNA from the p*Tad*NaC6–IR–EGFP vector in CHO cells produced robust acid-sensitive depolarizing leak currents recorded under a whole-cell voltage clamp when the membrane voltage was held at −60 mV (Fig. 2*D*). Leak inward currents were considerably active upon perfusion with an extracellular saline of pH of 7.5, whereas an acidic external solution of pH 5 completely blocked channel currents, and pH 9 and 10 solutions elicited maximal inward currents. *Tad*NaC6 thus forms a functional homomeric channel *in vitro*, with gating features similar to those of vertebrate BASIC and *C. elegans* ACD-1 channels, all conducting constitutive cation leak currents at rest that are not activated by extracellular protons (5, 11). This is in contrast to the ASIC channels, which become transiently activated upon extracellular acidification (32, 33). Furthermore, similar to ACD-1 and mouse BASIC, *Tad*NaC6 is blocked by extracellular protons. To our knowledge, this is the first report of acid sensitivity for a DEG/ENaC channel from an early-diverging metazoan. Instead, the cloned channels from the cnidarian *H. magnipapillata*, which form a clade with *Tad*NaCs and vertebrate ASIC and BASIC channels (Fig. 1*A*), were reported insensitive to pH and activated by RF-amide neuropeptides *in vitro* (14, 34).

Evidently, the kinetics of *Tad*NaC6 macroscopic currents change with pH, with desensitization becoming apparent after transition from a pH 5 to a pH 8.5 external solution, and more pronounced when transitioning from pH 5 to pH 10 (Fig. 2*D*). In this regard, relief from proton binding/inhibition, which serves to activate and then desensitize *Tad*NaC6, appears similar to proton activation of the ASIC channels. Thus, it appears as though activation and subsequent desensitization of *Tad*NaC6 are strictly dependent on transition between single-channel states, caused by dissociation of protons. We note that the current onset at pH 7 and 8 reflects slower channel activation compared with the intermediate pH of 7.5, as well as the more basic pH conditions ≥8.5. The slow activation kinetics at pH 8 mirror the steady-state current, where proton block of *Tad*NaC6 is biphasic through this pH range, comprising a high-affinity proton block with an average IC_50_ pH of 8.5 ± 0.04 (plus or minus standard error, *n* = 9) and a low-affinity block with an IC_50_ of pH 7.12 ± 0.03 (Fig. 2*E*). Therefore, under these experimental conditions having external solutions bearing 140 mm [Na^+^] and 2 mm [Ca^2+^], *Tad*NaC6 appears to have at least two extracellular binding sites for protons that affect both gating and activation kinetics.

### TadNaC6 bears hallmark structural features of the DEG/ENaC superfamily

**Figure 3.**
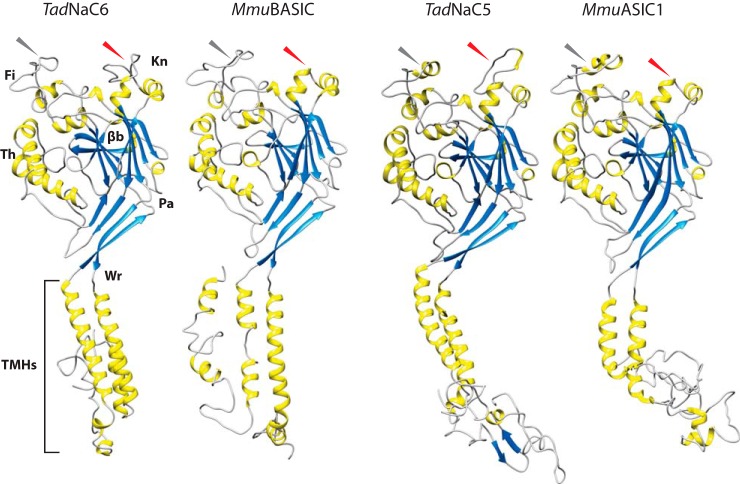
**Structural modeling of *Tad*NaC6, mouse (*Mus musculus, Mmu*) BASIC, *Tad*NaC5, and mouse ASIC1 with Phyre2.** Predicted α-helices, β-strands, and loops are colored *yellow*, *blue*, and *gray*, respectively. *TMHs*, transmembrane helices; *Wr*, wrist domain; *Pa*, palm domain; β*b,* β-ball domain; *Kn,* knuckle domain; *Fi,* finger domain; *Th,* thumb domain. *Red* and *gray arrow heads* indicate variable regions within the finger and knuckle domains as described in the text. Confidence in the models is shown as follows: 403 residues (82%) modeled at >90% accuracy for *Tad*NaC6; 440 residues (89%) modeled at >90% accuracy for *Mmu*ASIC5; 403 residues (74%) modeled at >90% accuracy for *Tad*NaC5; and 415 residues (79%) modeled at >90% accuracy for *Mmu*ASIC1.

**Figure 4.**
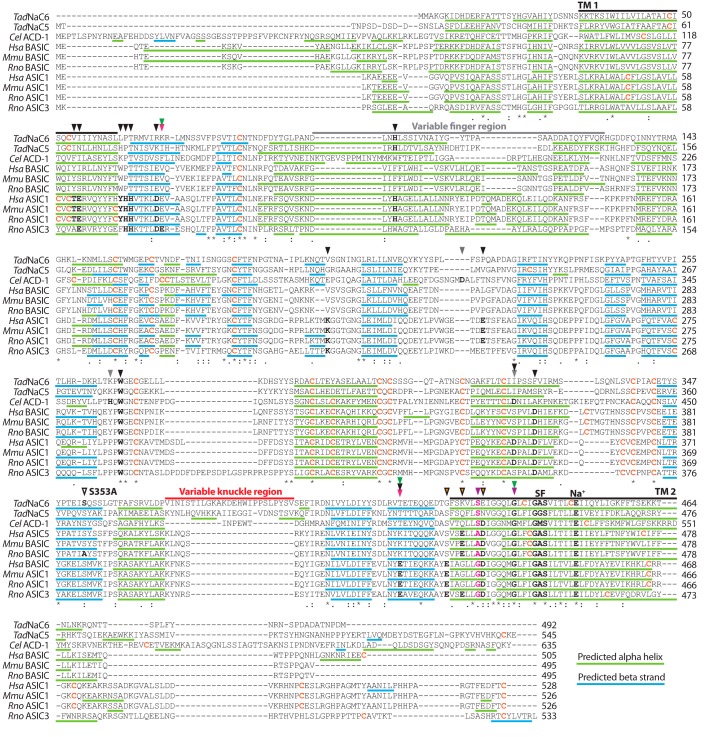
**MUSCLE protein alignment of *Tad*NaC6 and *Tad*NaC5 with *C. elegans* ACD-1, ASIC, and BASIC channels from human (*Hsa*), mouse (*Mmu*), rat (*Rno*), and rat ASIC3.** *Black arrow heads* denote amino acids positions previously shown to be important for proton activation of ASIC channels (36, 37, 64, 70, 82, 83), and *gray arrow heads* amino acid positions involved in proton-inhibition of ACD-1 (11). *Arrow heads with black outlines* denote residues associated with Ca^2+^ block of ASIC channels (*orange fill*) (66, 67) and BASIC channels (*no fill*) (5). *Pink arrow heads* denote amino acids previously shown to be required for the activation of rat ASIC3 by amiloride (23); *purple arrow heads* denote glycine amino acids in ASIC2 that rendered the channel amiloride-activated upon mutation to valine (G430V) and cysteine (G437C) (24); and *green arrow heads* denote amino acids associated with sensitivity to block by diminazene (75). The *bold pink residues* in transmembrane helix 2 denote the degenerin locus (74). Cysteines, which can form extracellular disulfide bridges, are shown in *bold orange. Colored underlines* represent amino acid sequences predicted with Phyre2 to form α-helices (*green*) and β-strands (*blue*), and *black lines* denote transmembrane helices (TM1 and TM2).

Structural modeling of *Tad*NaC6, as well as the other *Tad*NaC channel subunits, predicts conserved transmembrane helices that combine with helices from two additional subunits in the trimeric channel complex to form a functional six-helix ion-conducting pore (Fig. 3 and Fig. S1). In addition, the *Trichoplax* channels all are predicted to possess the six hallmark extracellular “ball-in-hand” structures of the DEG/ENaC channel family: 1) a palm domain made up of an array of closely-packed β-strands; 2) a wrist domain, which connects the palm domain to the transmembrane helices; 3) a thumb domain, important for channel ligand gating and made up of two tightly disulfide-linked α-helices; 4) a finger domain, comprising three α-helices and thought to regulate gating via interactions with the thumb domain; 5) a β-ball, made up of four β-strands; and 6) a knuckle domain, made up of two closely-linked α-helices (35). Models of *Tad*NaC3, *Tad*NaC6, and *Tad*NaC7 lack predictions for one of the three α-helices in the finger domain (helix 2), similar to models of the vertebrate BASIC channels that bear a reduced or absent helix in this region (Fig. 3 and Fig. S2). In contrast, *Tad*NaC2, *Tad*NaC5, *Tad*NaC8, *Tad*NaC9, and *Tad*NaC10 all bear three predicted finger helices, whereas *Tad*NaC2, *Tad*NaC9, and *Tad*NaC10 bear an additional predicted helix between the thumb and wrist domains, absent in the other analyzed channels except for nematode ACD-1. Notably, the finger domain is one of the most variable extracellular regions among the different classes of the DEG/ENaC channels (35). In accordance, a protein alignment reveals poor sequence homology in this region and, furthermore, a variability in sequence length with respective gaps in the alignment of 26 for *Tad*NaC6, 24 for *Tad*NaC5, 23 for BASICs and ASIC3, as compared with the longer sequences for ASIC1 (only 16 gaps) and ACD-1 (10 gaps; Fig. 4). Also notable is that most *Tad*NaCs bear insertions between the two knuckle helices, predicted to form loops that are absent in models of all vertebrate ASIC and BASIC channels, and *C. elegans* ACD-1 (Fig. 3 and Figs. S1 and S2).

Consistent with an independent radiation of DEG/ENaC channels within the Placozoa, aligned *Trichoplax Tad*NaC6 and *Tad*NaC5 proteins share more sequence features with each other than they do with homologues from vertebrates and *C. elegans* (Fig. 4). Nevertheless, both *Trichoplax* channels share with the other channel predictions for two conserved transmembrane helices, the second bearing more sequence homology, including the canonical “G*X*S” selectivity filter motifs crucial for Na^+^ selectivity and, overall, similar predicted secondary structures throughout the alignment (Fig. 4). Extracellular cysteine residues, which are important for stabilizing extracellular secondary structures, are particularly conserved, and of the 33 identical amino acids that span the alignment between transmembrane helices 1 and 2, 11 (33%) are cysteines. Glycines, which promote formation of loops between secondary structures, are also highly conserved at six positions (*i.e.* 18%), whereas 11 of the remaining 16 amino acids are hydrophobic (∼69%). Finally, we note the absence of key residues in *Tad*NaC6 that are conserved in deuterostome ASIC channels, which are crucial for proton activation, including a histidine located between transmembrane helix 1 and β-strand 1, and a lysine located in the loop between β-strands 5 and 6 (36, 37).

### Ion selectivity profiling of TadNaC6 reveals a moderate preference for Na^+^ over K^+^ ions, similar to other DEG/ENaC channels

**Figure 5.**
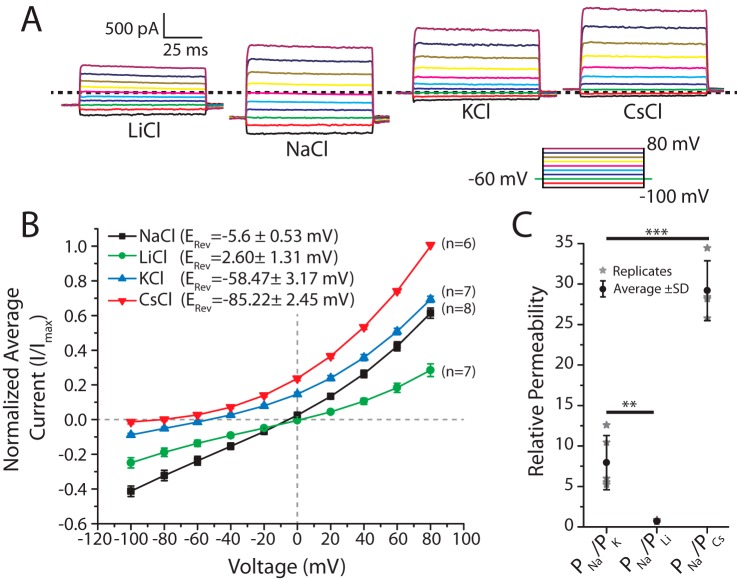
*A,* sample voltage-clamp macroscopic current traces of *Tad*NaC6 channels recorded at different voltages, using an invariant internal recording solution of 150 mm Na^+^, and perfused external solutions of 150 mm *X*^+^ ions where *X* = Li, Na, K, or Cs. The *dotted line* represents a current amplitude of zero. *B,* current-voltage plot of average peak inward and outward currents conducted by *Tad*NaC6 under different bi-ionic conditions. *C,* scatter plot of permeability of Na^+^ relative to *X*^+^ for *Tad*NaC6 reveals a preference for conducting Na^+^ over K^+^ and Cs^+^ and a higher selectivity for Li^+^. *Asterisks* denote *p* values for Bonferonni means comparison tests, after one-way ANOVA (*p* value of 4.7 × 10^−9^), with ** indicating a *p* value < 0.005 and *** denoting a *p* value < 0.0005.

DEG/ENaC ion channels are highly to moderately selective for Na^+^ over K^+^ ions (38). To evaluate the ion selectivity of *Tad*NaC6, we conducted bi-ionic reversal-potential experiments using 150 mm [Na^+^] in the internal solution and external perfusion of solutions bearing either 150 mm [Li^+^], 150 mm [Na^+^], 150 mm [K^+^], or 150 mm [Cs^+^] (Fig. 5). A voltage step to −100 mV produced the largest amplitude inward current when Na^+^ was present in the external environment and was sequentially decreased in amplitude upon replacement of Na^+^ with external Li^+^, K^+^, and Cs^+^. Sequential voltage steps between −100 and +80 mV allowed determination of the macroscopic current reversal potential for each bi-ionic condition, which is the voltage at which inward X^+^ ion currents reverse to outward Na^+^ currents, determined by the pore's preference for X^+^ ion over Na^+^. Not surprisingly, equimolar concentrations of Na^+^ across the cell membrane produced a reversal potential close to 0 mV (−5.6 ± 0.5 mV, *n* = 8), with a slight outward rectification at voltages more positive than +20 mV (Fig. 5*B*). In these experiments, a rightward shift in reversal potential relative to external Na^+^ reflects an increased permeability for the X^+^ ion relative to Na^+^, whereas a leftward shift indicates decreased permeability. Despite external Li^+^ producing smaller amplitude inward and outward macroscopic currents compared with external Na^+^, the reversal potential for Li^+^ is about 7 mV shifted toward the right, similar to what was reported for the vertebrate ASIC1 channel (39). Comparison of the Na^+^ and Li^+^ reversal potentials with the Goldman–Hodgkin–Katz equation reveals a slight preference for Li^+^ over Na^+^ (*P*_Li_/*P*_Na_ = 1.46 ± 0.07, *n* = 6; Fig. 5*C*). However, the smaller amplitude macroscopic currents with Li^+^ in the external environment suggest that although this smaller radius ion is better at occupying the pore, its conductance through the pore is not as efficient as it is for Na^+^, similar to ASIC1 (39). In summary, *Tad*NaC6 is similar to other DEG/ENaC channels, exhibiting a roughly 7-fold preference for Na^+^ over K^+^ (*P*_K_/*P*_Na_ = 0.14 ± 0.02, *n* = 5), and a 29-fold preference for Na^+^ over the large radius Cs^+^ ion (*P*_Cs_/*P*_Na_ = 0.03 ± 0.00, *n* = 4).

### Ca^2+^ block experiments uncover an interplay between proton and Ca^2+^ sensitivity for TadNaC6 and a unique residue involved in proton block

**Figure 6.**
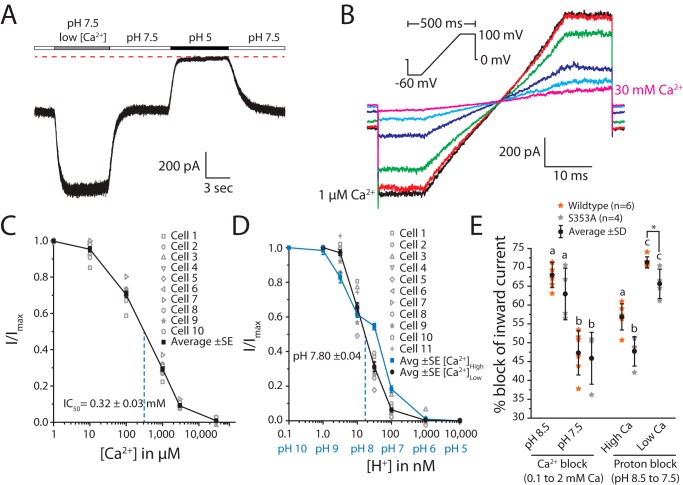
*A,* sample macroscopic current recording at −60 mV illustrating that *Tad*NaC6 is blocked by external Ca^2+^ ions, where perfusion from a pH 7.5 solution with 2 mm Ca^2+^ (*white bars*) to one with 0.1 mm Ca^2+^ (*gray bar*) roughly doubled the leak inward current amplitude. Instead, inward currents were completely blocked by a pH 5 solution in 2 mm Ca^2+^. *B,* sample currents elicited using a voltage ramp protocol (*inset*) recorded while perfusing different external solutions bearing 1 μm to 30 mm external Ca^2+^. *C,* average maximal inward current at −60 mV was plotted to generate an external Ca^2+^ dose-response curve for *Tad*NaC6. Ca^2+^ ions block the channel with an IC_50_ of 0.32 ± 0.03 mm. *D,* proton block for *Tad*NaC6 with low (0.1 mm) external Ca^2+^ produced a monophasic dose-response curve (*black line*), quite distinct from the biphasic curve observed with 2 mm external Ca^2+^ (*blue line*), with an IC_50_ of pH 7.8 ± 0.04. *E,* plot of average percent block of peak inward current plus/minus standard error for the WT and S353A mutant *Tad*NaC6 channel, upon transitioning from 0.1 to 2 mm [Ca^2+^]_ext_ and from pH 8.5 to 7.5. *Letters above bars* denote statistical differences between means, determined by one-way ANOVA and Bonferroni means comparison tests, with *p* values < 0.005. The *asterisk* denotes a detected statistically significant difference in a two-sample *t* test, with a *p* value < 0.05.

Many DEG/ENaC channels, including ASIC and BASIC channels and nematode ACD-1, are blocked by external Ca^2+^ ions. Notably, the highly homologous BASIC channels from mouse and rat differ in their sensitivity to Ca^2+^ when expressed in *Xenopus* oocytes, where physiological external [Ca^2+^] of ∼2 mm almost completely blocks rat BASIC, but has little effect on the mouse channel (5). However, this difference was not observed when the channels were expressed in the human cell line HEK293 (4). Nevertheless, given the similar functional properties of *Tad*NaC6 compared with BASIC channels, we thought it instructive to assess its sensitivity to external [Ca^2+^]. Increasing external [Ca^2+^] from 0.1 to 2 mm roughly halved leak inward current amplitude for *Tad*NaC6 at −60 mV, which could be completely blocked by perfusion of a pH 5 solution (Fig. 6*A*). Thus, Ca^2+^ is an external blocker of *Tad*NaC6. To generate a dose-response curve, we ran a voltage ramp protocol on patched cells while perfusing external solutions containing increasing [Ca^2+^] from 1 μm to 30 mm (Fig. 6*B*). Ca^2+^ produced a maximal block at 30 mm [Ca^2+^]_ext_ and an IC_50_ of 0.32 ± 0.03 mm (*n* = 10). This value contrasts the much more sensitive IC_50_ values of vertebrate BASIC channels expressed in HEK293 cells, with reported [Ca^2+^]_ext_ IC_50_ values of 38 ± 2 μm for rat, 6 ± 0.01 μm for mouse, and 12 ± 0.5 μm for human BASIC (4). Instead, Ca^2+^ block for *Tad*NaC6 appears more similar to that of mouse BASIC expressed in *Xenopus* oocytes, with an IC_50_ of 2.3 ± 0.2 mm, and *C. elegans* ACD-1, also in oocytes, for which macroscopic current amplitudes declined by only half when external Ca^2+^ was increased from nominal levels to 1 mm (11).

Next, we sought to assess the effect of external [Ca^2+^] on proton block of *Tad*NaC6 Na^+^ leak currents. Reducing external [Ca^2+^] from 2 to 0.1 mm caused the biphasic dose-response curve for proton block to resolve into a monophasic curve, with an IC_50_ of 15.8 nm [H^+^] (*i.e.* pH 7.8 ± 0.04, *n* = 11; Fig. 6*D*). Interestingly, the proton block was considerably diminished in low external [Ca^2+^] at pH 8.5, where block of maximal leak inward current at pH 9 declined from 17% in 2 mm [Ca^2+^] to 3% 0.1 mm [Ca^2+^]. Instead, a reversal in proton sensitivity is apparent at pH 7.5, where in 2 mm [Ca^2+^]_out_ only 46% of the maximal current is blocked by protons, and 69% is blocked in 0.1 mm [Ca^2+^]_out_. Clearly, the biphasic proton sensitivity of *Tad*NaC6 depends on external Ca^2+^ ions, possibly attributable to conformational changes induced by Ca^2+^ binding that alters the affinities/and or exposure of proton-binding sites along the extracellular channel surface. In this regard, *Tad*NaC6 is different than ACD-1, for which proton block was shown to be insensitive to [Ca^2+^]_ext_ (11).

Notably, the differences in external Ca^2+^ sensitivity between rat and mouse BASIC channels expressed in oocytes was attributed to a single amino acid, where an alanine to serine mutation at this position caused the rat channel to lose its micromolar sensitivity to calcium. Indeed, all *Tad*NaC channels, including *Tad*NaC6 and *Tad*NaC5, bear a serine at this position similar to the less calcium-sensitive mouse ASIC5 (Fig. 4). We therefore reasoned that the reverse mutation, serine to alanine, might render *Tad*NaC6 more sensitive to Ca^2+^ and perhaps alter the differences in proton block observed in high versus low [Ca^2+^]_ext_. At pH 8.5, increasing [Ca^2+^]_ext_ from 0.1 to 2 mm blocked peak inward current at −60 mV by 67.94 ± 1.36% S.E. for the WT channel (*n* = 6) and 62.93 ± 3.42% S.E. for the S353A mutant (*n* = 4) (Fig. 6*E*). At the more acidic pH of 7.5, both channel variants were less sensitive to Ca^2+^, with respective average block in response to increased calcium of 47.29 ± 2.39% S.E. and 45.87 ± 3.44% S.E. Thus, not only do external Ca^2+^ ions affect proton block for *Tad*NaC6 but, inversely, external protons affect the Ca^2+^ block in an antagonistic manner. Nevertheless, comparing averages for Ca^2+^ block between the WT and S353A channel variants by one-way ANOVA and Bonferroni mean comparison failed to show significant differences at either pH, indicating that the Ser-353 residue does not affect Ca^2+^ sensitivity of the channel, at least at these concentrations in CHO cells. Instead, this residue appears to be involved in proton sensitivity, where in 2 mm [Ca^2+^]_ext_ an increase in [H^+^]_ext_ via perfusion from pH 8.5 to 7.5 caused a 56.85 ± 1.42% block for the WT channel, compared with only 47.70 ± 1.95% for the S353A mutant. Thus, within this pH range, the mutant channel appears to be less sensitive to proton block compared with WT. Similarly in low [Ca^2+^]_ext_ (0.1 mm), proton block was less sensitive for the mutant channel with only 65.63 ± 1.94% block compared with 71.32 ± 0.59% for WT; however, a statistically significant difference was only observed using a two-sample *t* test (*p* < 0.05) and not one-way ANOVA. Also notable is that proton block is more pronounced in low *versus* high [Ca^2+^]_ext_ for both channel variants, at least in the pH range examined here, a feature similarly reported for the ASIC1 channel (40) that once again reveals an antagonistic interplay between proton block and Ca^2+^ block for *Tad*NaC6.

### Amiloride, a general DEG/ENaC blocker, is a strong agonist of TadNaC6

DEG/ENaC ion channels are often referred to as amiloride-sensitive sodium channels, attributable to their near-ubiquitous sensitivity to blocking by this drug. Interestingly, the rat ASIC3 channel was found to be directly activated by amiloride, where application of the drug produced large, nondesensitizing inward currents at a physiological pH of 7.4, quite distinct from the rapidly activating and desensitizing proton-activated currents observed at pH 5 (23). This atypical sensitivity to amiloride was attributed to two glutamate amino acids, one adjacent to transmembrane helix 1, and the second preceding transmembrane helix 2 (Fig. 4), since mutation of either of these two residues to alanine nearly abolished amiloride activation. At these same positions, *Tad*NaC6 bears a lysine and threonine, respectively. The human ASIC2 channel, which is normally blocked by amiloride, also becomes amiloride-activated upon mutation of two glycines within transmembrane helix 2, G430V and G437C (24). Notably, although Gly-437 appears to be conserved even in the *Trichoplax* channels and ACD-1, the Gly-430 position bears a serine for *Tad*NaC6, *Tad*NaC5, and ACD-1 and an alanine for vertebrate BASIC channels (Fig. 4).

**Figure 7.**
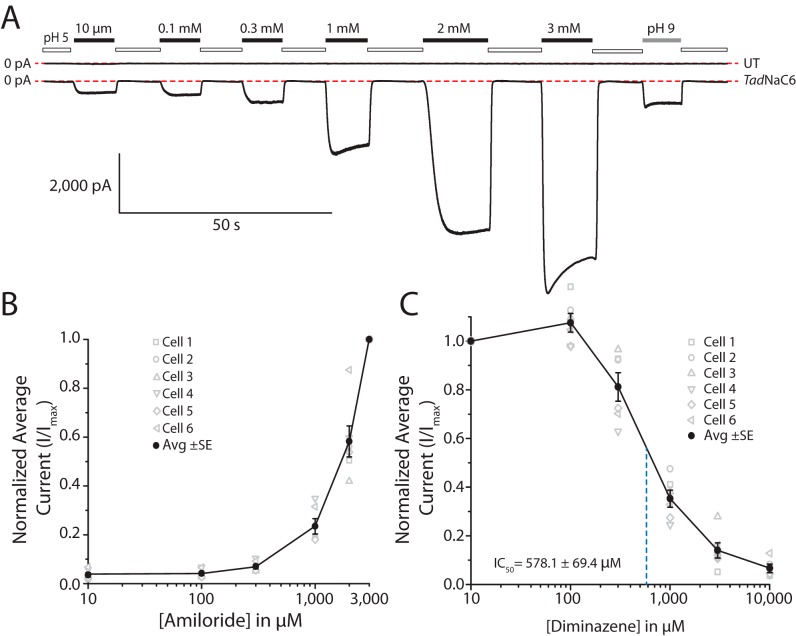
*A,* amiloride, a general blocker of DEG/ENaC ion channels, is a nonproton agonist of *Tad*NaC6. Perfusion from a pH 5 solution (*white bars*) to pH 7.4 solutions bearing increasing concentrations of amiloride (10 μm to 3 mm; *black bars*) produced increasingly larger macroscopic currents, considerably larger than those elicited by a pH 9 solution (*gray bar*). *UT* refers to untransfected cells. *B,* average peak inward currents elicited by perfusion of increasing concentrations of amiloride at pH 7.5, relative to a pH 5 solution. *C,* diminazene, another general blocker of DEG/ENaC channels, blocked average *Tad*NaC6 macroscopic currents with an average IC_50_ of 578.1 ± 69.4 μm.

Perfusion from a pH 5 solution (where all channels would be closed) to pH 7.5 solutions bearing 10–300 μm amiloride produced sustained nondesensitizing inward currents that increased slightly with increasing concentrations of the drug (Fig. 7). However, transition from pH 5 to 7.5 would activate *Tad*NaC6 currents such that the effect of amiloride is masked by channel activation elicited by relief from the proton block. Nevertheless, perfusion of 1 mm amiloride at pH 7.5 produced large amplitude inward currents, considerably larger than those elicited by pH 9 solution without amiloride. Furthermore, 3 mm amiloride produced even larger *Tad*NaC6 currents, roughly 12-fold greater than those elicited by pH 9. Thus, similar to ASIC3, amiloride is a nonproton ligand of *Tad*NaC6. Unlike amiloride-activated currents for ASIC3, *Tad*NaC6 currents were marked with a moderate desensitization component (Fig. 7*A*), resembling amiloride-activated currents of the mutant human ASIC2 channel (24). Instead, *Tad*NaC6 currents were weakly blocked by the phenylhydrazine compound diminazene (Fig. 7*C*), another general blocker of DEG/ENaC channels, with an IC_50_ of 578.1 ± 69.4 μm. The HyNaC channels from *Hyd*ra were found to be much more sensitive to diminazene, with IC_50_ values ranging from 0.05 to 31.4 μm (13). Similarly, vertebrate ASIC and BASIC channels are also blocked by micromolar concentrations of diminazene, with reported IC_50_ values ranging from 2 to 16.8 μm (4, 33). Thus, whereas diminazene blocks *Tad*NaC6 (albeit with a low affinity), amiloride acts as a potent agonist, indicating that the two compounds elicit different allosteric changes in the channel's structure. Furthermore, the lack of conserved amino acid determinants associated with amiloride activation suggests that different mechanisms are responsible for this phenomenon in *Tad*NaC6.

## Discussion

### On the phylogenetic relationships of DEG/ENaC channels

Here, we describe the *in vitro* characteristics of *Tad*NaC6, one of 11 DEG/ENaC ion channel subunit genes identified for the early-diverging animal *T. adhaerens*. Our phylogenetic analysis corroborates previous reports that all but one of the *Trichoplax* channels form a sister clade relationship with the acid-activated ASIC channels from chordates (Fig. 1*A*). Instead, the vertebrate sodium leak channels BASIC and ENaC are each phylogenetically separate, whereas the remaining *Trichoplax* channel, *Tad*NaC10, forms a clade with ENaCs, mollusc FaNaCs, and nematode DEG/MEC/DEL channels. Two prominent features of phylogenetic trees inferred from DEG/ENaC channel protein alignments are as follows: 1) extensive incomplete lineage sorting, where phylogenetic relationships of channel homologues do not correspond with the species phylogeny, and 2) frequent and independent expansion of DEG/ENaC channels within distinct animal groups. With respect to feature 1, it is striking that the major clade of *Trichoplax* channels is not separated from vertebrate ASIC channels by homologues from protostome invertebrates such as arthropods, nematodes, or molluscs, although they appear broadly in the deuterostomes such as early-diverging chordates (*i.e. Branchiostoma belcheri* and *Oikopleura dioica*), hemichordates (*Ptychodera flava*), and echinoderms (*Strongylocentrotus purpuratus* and *Acanthaster planci*) (Fig. 1*A*) (36). Thus, either the ancestor of all protostome invertebrates lost an ASIC-like homologue, there was lateral gene transfer, or the *Trichoplax* and chordate channels converged at the amino acid sequence level. With respect to feature 2, it is notable that the majority of clades bearing strong bootstrap support consists of channels from a single phylum/species, consistent with the notion that the DEG/ENaC channel family has undergone extensive independent expansion within different animal lineages (12). Interestingly, similar expansion events are evident for transient receptor potential channels (41) and ionotropic glutamate receptors (42, 43), which like DEG/ENaC channels were frequently adapted for sensory functions. Extensive gene gain/loss is also evident for chemosensory G-protein–coupled receptors, where it has been proposed that a combination of adaptation to different sensory environments, coupled with random gene gain/loss, accounts for observed phylogenetic differences (44).

*In situ* hybridization of *Tad*NaC6 revealed mRNA expression enriched along the periphery of the animal (Fig. 1*B*), a region occupied by cells that secrete mucus and label for the endomorphin-like peptide (*Ta*ELP) that causes *Trichoplax* ciliary locomotion to cease (19, 45). *Tad*NaC5 was expressed near the outer edge of the animal; however, its expression reached deeper into the center, whereas *Tad*NaC2 appeared absent along the outer rim but more prevalent in the interior, a region enriched with lipophil cells (45). The localization of *Tad*NaC2 is consistent with the single-cell transcriptome study revealing co-expression of *Tad*NaC2, *Tad*NaC3, and *Tad*NaC8 in digestive/lipophil cells (31). Indeed, the sensitivity of *Tad*NaC6 to external protons begs the following question: when might the animal experience change in extracellular pH along its periphery? One obvious possibility is during feeding, where lipophil cells in the ventral epithelium are proposed to secrete large acidic vesicles that contain digestive enzymes into the extracellular environment upon detection of food microalgae, for the purpose of hydrolytic breakdown and digestion (16). Given the small volume between the ventral epithelium and the underlying substrate, it is conceivable that the contents of these secreted vesicles temporarily but significantly lower extracellular pH. By extension, cells expressing *Tad*NaC6 would undergo membrane hyperpolarization due to proton block of the channel. Alternatively, protons could serve modulatory functions for yet undiscovered ligands for *Tad*NaC6, such as secreted peptides, bile-like compounds, mechanical stress, and/or chemicals in the environment. Clearly, additional studies on this and other *Trichoplax* DEG/ENaC channels are needed to further characterize their intrinsic functional properties and furthermore to elucidate their roles in physiology and behavior.

### Structural and functional properties of TadNaC6

Modeling the tertiary structures of the nine cloned *Trichoplax* DEG/ENaC channels revealed predicted extracellular “ball-in-hand” structures common to all DEG/ENaC channels (Fig. 3 and Fig. S1). Although these predictions are to be interpreted lightly, it is interesting that the *Trichoplax* channels distinguish themselves from the vertebrate ASIC and BASIC channels, as well as the nematode ACD-1 channel, with notable insertions between the two α-helices that make up the knuckle region (Figs. 3 and 4 and Fig. S1). Based on the crystal structure of the functional trimeric chick ASIC1 channel, the knuckle domains from three separate subunits come together at the extracellular face to give rise to an upper vestibule of the channel pore (46). For ENaC channels, the knuckle domain has been implicated in sodium self-inhibition (47), indicating that structural changes in this region have the capacity to regulate DEG/ENaC channel gating. Another region with notable variability is the finger domain, where one of the three expected α-helices was not predicted for *Tad*NaC3, *Tad*NaC4, *Tad*NaC6, and *Tad*NaC7 (*i.e.* helix 2). It is interesting that the vertebrate ASIC and BASIC channels also differ in this region, with ASIC channels bearing strong predictions for all three helices, while the BASIC channels lack predictions for helix 2. Indeed, the finger domain has been flagged as the most divergent extracellular region for DEG/ENaC channels and hence a proposed locus for functional variability between different channel classes (35).

Functionally, *Tad*NaC6 most resembles mouse BASIC and *C. elegans* ACD-1, conducting constitutive Na^+^ leak currents at resting membrane voltages that are blocked by external H^+^ and Ca^2+^ ions (Figs. 2 and 6) (4, 5, 11). Its ion preference is similar to that of most other DEG/ENaC channels, being roughly 7-fold selective for Na^+^ over K^+^ (Fig. 5). Accordingly, *Tad*NaC6 bears a GAS selectivity filter motif in transmembrane helix 2 and a key glutamate just downstream of that, both important for Na^+^ selectivity (Fig. 4) (38, 48). Other reported DEG/ENaC Na^+^ leak channels include ENaC and the human/rat BASIC channels, which differ in that extracellular protons either potentiate (ENaC) or do not affect (BASICs) their macroscopic currents (49–51). ENaC is also considerably more selective for Na^+^ compared with other channels, with a *P*_Na_/*P*_K_ ratio of roughly 100 (52). In *Drosophila*, the DEG/ENaC channels ripped pocket (RPK) and pickpocket (PPK), associated with sensory functions are Na^+^ leak channels, of which PPK1 was shown to be activated by extracellular protons (53, 54). Phylogenetically, it would appear as though *Tad*NaC6, ENaC, BASIC, ACD-1, and PPK/RPK each evolved the capacity to conduct constitutive Na^+^ leak currents independently, because they are separated by clades of channels that exhibit transient activation in response to various stimuli (Fig. 1*A*). For example, although *Tad*NaC6 and BASIC channels sit relatively close to each other on the phylogenetic tree, they are separated by the proton-activated ASIC channels, as well as several acid-activated *Trichoplax* channels that we are currently characterizing in the laboratory. Thus, assuming that the apparent phylogenetic relationships within this group are true, the ancestral channel was either (i) a transiently-activatable channel, such that constitutively open leak channels evolved independently in BASICs and *Tad*NaC6, or alternatively, (ii) the ancestral channel was a leak channel, such that proton activation evolved independently in ASICs and the *Trichoplax* acid-activated channels. More phylogenetically distant to *Tad*NaC6 are the vertebrate ENaC channels, which are nestled within a group of transiently activated mechanosensitive and neuropeptide-gated channels, and unlike *Tad*NaC6, BASICs, and ACD-1, ENaCs do not form functional homotrimers but instead operate as obligate heterotrimers of α, β, and γ subunits (55). Finally, ACD-1 and RPK/PPK channels are located within separate clades with no clear phylogenetic link to *Tad*NaC6 and other Na^+^ leak channels. Interestingly, ASIC channels have recently been proposed to exhibit two distinct conductance states (56), a fast-desensitizing conductance triggered by extracellular protons, and a slow-nondesensitizing conductance triggered by nonproton ligands such as the coral snake venom MitTx (57), neuropeptides (58, 59), and the polyamine 2-guanidine-4-methylquinazoline (GMQ) (60). Similarly, GMQ potently activates the mollusc FaNaC channels through a distinct mechanism than their natural ligand FMRF-amide (9), pointing to a conserved and ancient duality in DEG/ENaC channel gating. Considering this, the apparent homoplasy in Na^+^ leak current activity for phylogenetically distant members of the superfamily might instead be attributable to adaptive changes that independently enhanced sustained conductance properties among distinct channel lineages.

### General absence of determinant residues associated with proton sensitivity

Initially, it was thought that proton-activated ASIC channels evolved strictly in the bony fish (61). However, a subsequent *in vitro* study on the shark homologue ASIC1b revealed proton activation, attributed to a conserved set of histidine residues adjacent to transmembrane helix 1 (Fig. 4), where similar to rat ASIC1a, mutation completely disrupted proton sensitivity (62). Nevertheless, acid activation was not observed for the cloned ASIC1 channel from lamprey (61) nor a tunicate ASIC-like homologue (63), suggesting proton activation is unique to vertebrates. More recently, a comprehensive study determined that acid-activated channels exist more broadly, where specifically cloned homologues from various basal deuterostome animals conducted proton-activated currents *in vitro* (36). Indeed, numerous amino acids have been implicated in proton activation of ASIC channels (Fig. 4); however, this recent study perhaps identified the most crucial and conserved: 1) one of the histidines adjacent to transmembrane 1 mentioned above (*i.e.* His-73 in mouse ASIC1a), and 2) a lysine located in a linker between the β-ball and palm domains (Lys-211). In accordance, mutation of either of these two residues in the divergent deuterostome channels severely disrupted or completely abolished proton sensitivity (36). The question whether the mechanisms for proton activation of ASIC channels relates to proton block of other channels was addressed for ACD-1. Notably, the proton block was found to involve three amino acids (11), only one of which, an aspartate located in the thumb domain, is conserved with proton-sensitive acidic residues in ASIC channels (Fig. 4). Indeed, outside of an additional conserved tryptophan at the base of the thumb domain that is thought to interact with a tyrosine in transmembrane helix 1 of ASIC1 channels (64), ACD-1 lacks most amino acids implicated in proton sensitivity for ASIC channels. Hence, the mechanisms by which protons affect the gating of this particular channel are quite distinct and likely independently evolved from those of ASIC channels. Similarly, *Tad*NaC6, which is considerably sensitive to protons, lacks all but two amino acids associated with proton sensitivity in other channels, sharing none with ACD-1. As mentioned above, the mouse BASIC channel is also blocked by protons; however, it is much less sensitive than ACD-1 and *Tad*NaC6, where the highly-acidic extracellular pH of 4.0 only reduces the amplitude of macroscopic current by about 40% (5). To our knowledge, the amino acids associated with proton block of mouse BASIC have not yet been identified. Nevertheless, given the general lack of conservation of amino acid residues associated with proton sensitivity among these phylogenetically distant Na^+^ leak channels, it seems likely that they evolved proton sensitivity independently from each other, as well as ASIC channels.

### Interplay between Ca^2+^ block and proton block

An early study into the mechanisms for proton activation of ASIC channels (*i.e.* rat ASIC3) identified an interplay between external protons and Ca^2+^, where reducing external Ca^2+^ allowed protons to more potently activate the channel (65). Furthermore, removal of external Ca^2+^, at a constant pH, directly activated ASIC3, indicative of the block of ASIC currents by external Ca^2+^ ions at physiological concentrations. These observations led to the proposal that proton activation of ASIC channels involves displacement of blocking Ca^2+^ ions by protons that effectively reduce the binding affinity of Ca^2+^ to a site on the extracellular surface of the channel. However, unlike ASIC3, Ca^2+^ depletion does not significantly activate rat ASIC1a (66). Furthermore, the mechanisms for proton and Ca^2+^ sensitivity for this channel appear to be functionally distinct, because mutation of two amino acids in transmembrane helix 2, Glu-425 and Asp-432 (Fig. 4), completely abolished Ca^2+^ block but did not disrupt proton activation nor create constitutively open channels, as would be expected based on the proposed Ca^2+^ displacement model of ASIC channel gating (66). A subsequent study identified an additional glutamate residue in transmembrane helix 2, unique to ASIC3, that accounts for its unique activation in response to Ca^2+^ depletion (Fig. 4), where a corresponding mutation in the rat ASIC1a channel (G429E) rendered it activatable by Ca^2+^ depletion similar to ASIC3 (67). Thus, it appears as though ASIC3 is unique among ASIC channels in its ability to become activated by external Ca^2+^ depletion. Nevertheless, it is interesting to note that currents elicited by Ca^2+^ depletion at a fixed pH of 8.0 where sustained and nondesensitizing (65), indicating that at basic pH, ASIC3 conducts constitutive Na^+^ leak currents that are sensitive to Ca^2+^ block, similar to those of *Tad*NaC6, ACD-1, and BASIC channels. Also interesting is that the aforementioned glutamate in transmembrane helix 2 of ASIC3 is also present in BASIC channels (Fig. 4); however, whether this residue is involved in Ca^2+^ block for BASIC channels has not been explored, to our knowledge.

As mentioned above, *in vitro* studies on rodent BASIC channels expressed in *Xenopus* oocytes revealed a differential sensitivity to Ca^2+^, where the rat channel was almost completely blocked by 1.8 mm [Ca^2+^], while the mouse channel was minimally blocked at this same concentration (5). Instead, in a more recent study where the rat, mouse, and human BASIC channels were expressed in HEK293 cells, all three homologues were found to be highly sensitive to Ca^2+^ block, indicating that Ca^2+^ sensitivity might depend on the properties of the membrane environment that are likely different between mammalian cells and frog oocytes (4). In accordance, exploration into the mechanisms by which bile acids activate BASIC channels has revealed that the mode of action of these and other membrane-active compounds is likely through alterations in membrane environment, and not necessarily binding to channels *per se* (68). Nevertheless, we sought to determine whether the single serine amino acid associated with reduced Ca^2+^ sensitivity in mouse BASIC was involved in the low Ca^2+^ sensitivity observed for *Tad*NaC6. Our hypothesis was that mutation of this residue to alanine (S353A), making it similar to rat BASIC, would increase its sensitivity to Ca^2+^ block. We saw no change in Ca^2+^ block at pH 7.5 and 8.5 for the S353A mutant compared with WT, but instead, we observed a reduced sensitivity to proton block in both low and near physiological Ca^2+^ (Fig. 6*E*). Indeed, this study was not exhaustive, and the mechanisms by which Ca^2+^ and H^+^ ions bind to the *Tad*NaC6 channel to regulate its gating need to be explored further. However, it can be stated that similar to proton sensitivity, it is likely that the mechanisms for Ca^2+^ sensitivity of *Tad*NaC6 are highly divergent from other channels, because it lacks key amino acid determinants identified for other channels (Fig. 4).

Comparing the Ca^2+^ block of various Ca^2+^-sensitive Na^+^ leak DEG/ENaC channels reveals that *Tad*NaC6 is the less sensitive to Ca^2+^ than BASIC channels expressed in mammalian cells (4), and it is similar to mouse BASIC and nematode ACD-1 expressed in oocytes (11). Although *Tad*NaC6 was expressed strictly in mammalian cells, making it difficult to derive conclusions based on these comparisons, it is worth noting that the *Trichoplax* channel is adapted to a marine extracellular saline environment, in which [Ca^2+^] is roughly 10 mm. At this concentration, Ca^2+^ ions might therefore cause *Tad*NaC6 to remain predominantly in a closed/blocked state under physiological conditions, similar to what might be expected for BASIC channels. Something yet to be determined is whether Ca^2+^ modulation of *Tad*NaC6, BASIC, and ACD-1 channels serves a function *in vivo*. For ASIC channels, lactate released during muscle stress reduces the concentration of divalent cations in the extracellular environment and potentiates ASIC channel sensitivity to protons in ischemia-sensing neurons (69). Mechanistically, the interplay between Ca^2+^ block and proton block for *Tad*NaC6 appears to be quite unique. At 2 mm external Ca^2+^, proton block was biphasic, suggesting the existence of two distinct proton-binding sites (Fig. 2*E*), which to our knowledge has never been reported for a WT DEG/ENaC channel. Furthermore, the activation kinetics of the channel are slower than expected at pH 8 compared with pH 7.5 and 8.5, where, possibly, the biphasic dose-response observed for steady-state currents might be due in part to slowed activation of the macroscopic current when transitioning from pH 5 to 8 and/or accelerated activation from pH 5 to 7. Previously, several mutations of acidic residues in ASIC1a (E79K/E79Q, D345K/D345R, and E416K; Fig. 4) were found to cause the channel's normally monophasic proton activation curve to become biphasic, which the authors proposed reflects the existence of two distinct proton-binding sites that become apparent through mutagenesis (70). What is particularly notable is that one of these mutants, with a negatively-charged aspartate changed to a positively-charged lysine (E79K), resembles *Tad*NaC6 bearing an arginine in this position. For *Tad*NaC6, a reduction of external Ca^2+^ caused proton block to resolve to a monophasic response (Fig. 6*D*); however, whether this occurs for the mutant ASIC channels was not addressed. Regardless, as noted above *Tad*NaC6 lacks most residues associated with proton sensitivity in ASIC channels, and thus it is likely that the occurrence of two distinct proton-binding sites in ASIC and *Tad*NaC6 channels emerged through convergence. Similarly, the proton and Ca^2+^-gating dynamics of *Tad*NaC6 are likely fundamentally distinct from those of ACD-1, because proton block of the nematode channel was found to be completely independent of external [Ca^2+^] (11).

### Atypical pharmacology of the TadNaC6 channel

The drug amiloride blocks ENaC channels with high affinity (IC_50_ of ∼100 nm), hence its clinical use for high blood pressure where it causes decreased Na^+^ reabsorption by renal epithelial cells and increased excretion in the urine (71, 72). Amiloride also blocks ASIC channels and, in fact, most DEG/ENaC channels (73), such that the DEG/ENaC channel family is often alternatively referred to as the amiloride-sensitive ion channels. Interestingly, a study on a “degenerin” mutant of the human ASIC2a channel (*i.e.* G430V), a locus discovered in nematodes that when mutated renders DEG/ENaC channels constitutively open (Fig. 4) (74), revealed that introducing a second mutation adjacent to the selectivity filter (G437C) rendered the channel activated by amiloride rather than blocked (24). The authors proposed that ASIC channels might bear two distinct amiloride-binding sites, one in the pore where the drug blocks and another within an undiscovered location. The proposed model was thus that the G430V/G437C mutations disrupted amiloride binding in the pore and hence blocked and unmasked the capacity of amiloride to bind the second locus to trigger channel activation. A subsequent study on the WT rat ASIC3 channel revealed that it could be potently activated by amiloride, attributed to two glutamate residues in the so-called “nonproton ligand-sensing domain” (Glu-79 and Glu-423), located between the palm and wrist domains (60). Nevertheless, ASIC1 channels possess these same amino acids (Fig. 4) but are not activated by amiloride, indicating that additional structural features are involved. To our knowledge, whether there is an interplay between the G430V/G437C mutations in ASIC2a, and the Glu-79/Glu-432 residues in ASIC3, all conserved between ASIC1, ASIC2, and ASIC3 channels, remains unexplored. Notably, the Glu-79/Glu-432 residues were also shown to be involved in activation of ASIC3 by the nonproton agonist GMQ, which, like amiloride, generates nondesensitizing currents (60). Furthermore, GMQ was found to activate molluscan neuropeptide-gated FaNaC channels through amino acids within the nonproton ligand-sensing domain, independent of FMRF-amide activation (9), pointing to a deeply-conserved gating mechanism for DEG/ENaC channels that can be acted upon by GMQ and amiloride. Here, we provide a second report of a WT DEG/ENaC channel being activated by amiloride. *Tad*NaC6 currents elicited by 1–3 mm amiloride were far larger in amplitude that those elicited by increasing pH (Fig. 7, *A* and *B*). We note that the channel lacks the two glutamate residues in the nonproton-sensing domain of ASIC3 associated with amiloride activation. However, the channel bears a serine in the degenerin position corresponding to the G430V mutation in ASIC2a, and furthermore, a cysteine just two amino acids downstream from the Gly-437 position, which as mentioned above, caused the human channel to become amiloride-activated (Fig. 4).

Finally, it is interesting to note that the Glu-79/Glu-432 residues required for amiloride activation of ASIC3 were also found to be important for block of ASIC1a by diminazene, another general DEG/ENaC channel blocker. Single glutamine mutations at either of these glutamate residues reduced the channel sensitivity to diminazene by roughly 7-fold. Moreover, a glycine to alanine mutation in the Gly-437 position, associated with amiloride sensitivity of ASIC2a, increased diminazene sensitivity of ASIC1a by 1 order of magnitude (75). In addition, this same locus bears serine and glycine residues in the three ENaC channel subunits that are critical for amiloride block (76), where altogether, it appears as though amiloride and diminazene block DEG/ENaC channels by binding similar regions of the pore. Here, we found *Tad*NaC6 to be sensitive to block by diminazene, albeit with a relatively low-affinity IC_50_ value of ∼578 μm (Fig. 7*C*), which is fundamentally different from the effect of amiloride on this channel. Considering the model for paradoxical activation of ASIC3 by amiloride, it seems plausible that *Tad*NaC6 similarly possesses a secondary binding site for amiloride within the nonproton ligand-sensing domain, and poor affinity for the drug within the pore. Instead, diminazene might effectively bind within the pore to block the channel and/or lack the ability to bind the nonproton ligand-sensing domain. Clearly, future studies will be required to make sense of how these drugs regulate the gating of *Tad*NaC6, which might provide some unique perspectives on how these drugs modulate DEG/ENaC channel gating in general.

## Experimental procedures

### Fluorescence in situ hybridization

Animals were prepared for FISH by freezing in tetrahydrofuran overnight on dry ice followed by fixation in 3% acetic acid in methanol (MeOH) for 30 min at −20 °C and then 4% paraformaldehyde in methanol for 30 min at room temperature, as described (45). *In situ* hybridization was performed with RNAscope probes for ASIC6, ASIC5, and ASIC2 (#572821, #572811-C2, and 572801-C3, respectively) and Multiplex Fluorescent Assay reagents (#320850) from Advanced Cell Diagnostics (Hayward, CA). Fluorescence images were collected ×63 NA 1.4 objective on an LSM880 laser-scanning confocal microscope (Carl Zeiss Microscopy LLC, Thornwood, NY).

### In silico analyses

The maximum likelihood phylogenetic tree was inferred from a MUSCLE protein alignment (77) trimmed with trimAl (78) (gap-threshold of 0.95), using IQTree (79) with the model WAG+F+I+G4. Brach support values were generated via 2000 ultrafast bootstrap replicates. GenBank^TM^ accession numbers for protein sequences are as follows: *Homo sapiens* ASICs, hASIC1:NP_064423.2, hASIC2:NP_001085.2, hASIC3:NP_004760.1, hASIC4:NP_878267.2, and hASIC5:NP_059115.1; *Rattus norvegicus* ASICs, rASIC1:NP_077068.1, rASIC2:NP_001029186.1, rASIC3:NP_775158.1, rASIC4:NP_071570.2, and rASIC5:NP_071563.1; *Mus musculus* ASICs, mASIC1:NP_033727.1, mASIC2:NP_001029185.1, mASIC3:NP_892045.2, mASIC4:NP_898843.1, and mASIC5:NP_067345.1; *Gallus* ASICs, gASIC1:NP_001035557.1, gASIC2:XP_418066.2, gASIC3:XP_025003153.1, gASIC4:XP_001232417.5, and gASIC5:XP_015140827.1; *B. belcheri* ASIC, BbASICa:XP_019621273.1; *O. dioica* ASIC, OdASICa:GSOIDP00000722001 (OikoBase); *S. purpuratus* ASIC, SpASICb:XP_780968.2; *A. planci* ASIC, ApASICb:gbr.173.6.t1 (OIST Marine Genomics); *P. flava* ASIC, PfASICb:pfl_40v0_9_20150316_1g14838.t1 (OIST Marine Genomics); *T. adhaerens* DEG/ENaC channels, TadNaC1:XP_002114386.1, TadNaC2:MK547543, TadNaC3:MK547544, TadNaC4:MK547545, TadNaC5:MK547546, TadNaC6:MK547547, TadNaC7:MK547548, TadNaC8:MK547549, TadNaC9:MK547550, TadNaC10:MK547551, and TadNaC11:XP_002114391.1; *Hydra vulgaris* peptide-gated channels, HyNaC9:NP_001296667.1, HyNaC6:CDG50527.1, HyNaC2:CAL36110.1, HyNaC7:CDG50528.1, HyNaC11:NP_001296594.1, HyNaC12:NP_001296710.1, HyNaC8:NP_001296668.1, HyNaC4:CAL36113.1, HyNaC5:CAX62741.1, HyNaC10:NP_001296656.1, and HyNaC3:CAL36112.1; human, rat, mouse, and chicken epithelial sodium channels, hENaCα:NP_001029.1, hENaCβ:NP_000327.2, hENaCγ:NP_001030.2, hENaCδ:AAI25075.1, rENaCα:NP_113736.1, rENaCβ:XP_008757904.1, rENaCγ:NP_058742.2, mENaCα:NP_035454.2, mENaCβ:NP_001258952.1, mENaCγ:NP_035456.1, gENaCα:NP_990476.2, gENaCβ:XP_015149983.1, gENaCγ:XP_015149986.1, and gENaCδ:XP_004947475.1; FRMFamide-gated sodium channels from molluscs *Helisoma trivolvis,* HtFaNaC:AAF80601.1; *Helix aspersa,* HaFaNaC:Q25011.1; *Lymnaea stagnalis,* LsFaNaC:AAK20896.1; *Aplysia californica,* AcFaNaC:XP_012938733.1; *C. elegans* DEG/ENaC channels ACD-1:NP_491295.2, ACD-2:NP_001309477.1, ACD-3:NP_001257250.1, ACD-4:NP_505230.1, ACD-5:NP_491196.3, DEL-1:Q19038.1, DEL-4:NP_492230.2, DEL-7:NP_501276.4, DEL-9:NP_508622.2, DEL-10:NP_495302.3, MEC-10:NP_509438.1, MEC-4:NP_510712.2, FLR-1:NP_510243.1, DEL-3:NP_492135.1, UNC-105:NP_001122595.1, and UNC-8:NP_501138.1; and *Drosophila melanogaster* DEG/ENaC channels, PPK4:NP_001334723.1, PPK5:NP_996138.2, PPK6:NP_611461.3, PPK7:NP_609016.2, PPK8:NP_001036260.3, PPK9:NP_611622.2, PPK10:NP_001033894.1, PPK11:NP_001334672.1, PPK12:NP_611672.1, PPK13:NP_001014495.1, PPK14:NP_609017.2, PPK15:NP_001097937.1, PPK16:NP_001334673.1, PPK17:NP_001285988.1, PPK18:NP_609308.3, PPK19:NP_651708.2, PPK20:NP_651705.2, PPK21:NP_651704.2, PPK22:NP_733051.2, PPK23:NP_001014749.1, PPK24:NP_651860.2, PPK25:NP_995766.1, PPK26:NP_648125.3, PPK27:NP_647826.2, and PPK28:NP_573169.2. Protein sequences for DEG/ENaC channels of *A. queensladica* were obtained via BLAST from EnsemblMetazoa with accession numbers as follows: AqNaC1:Aqu2.1.34376, AqNaC2:Aqu2.1.09805, AqNaC3:Aqu2.1.34363, AqNaC4:Aqu2.1.26218, AqNaC5:Aqu2.1.09804, AqNaC6:Aqu2.1.26214, AqNaC7:Aqu2.1.26220, AqNaC8:Aqu2.1.34361, AqNaC9:Aqu2.1.25935, AqNaC10:Aqu2.1.36244, AqNaC11:Aqu2.1.26219, AqNaC12:Aqu2.1.34365, AqNaC13:Aqu2.1.13200, and AqNaC14:Aqu2.1.20433. Protein sequences for DEG/ENaC channels of *Mnemiopsis leidyi* were obtained from a transcriptome assembly generated in-house, and those for *Beroe ovata* were obtained from Drs. Joseph Ryan and Mark Martindale (Whitney Laboratory, Florida) from gene model databases generated for genome assembly. All protein sequences are provided in FASTA format in File S1. Structural modeling of the tertiary structures of various DEG/ENaC channels homologues was achieved using the Phyre2 server intensive modeling mode (80). Confidence values for the resulting models are reported in the corresponding figure legends. All Protein Data Bank files for predicted tertiary structures are provided in the compressed File S2. Phyre2 was also used to predict secondary structures, depicted in a ClustalW protein alignment in Fig. 2 (81).

### In vitro expression and Western blotting

We previously reported the cloning of various *Tad*NaC ion channel cDNAs from whole-animal total RNA.^3^ For functional expression, the *Tad*NaC6 cDNA was PCR-amplified using primers *Ta*dNaC6-forward (5′-ATTATACTCGAGGCCGCCACCATGATGGCCAAGGGAAAAATCGATCACG) and *Ta*dNaC6-reverse (5′-ATTATAGGATCCTCTTTACATATCTGGATTTGTTGCATCAGC) bearing respective N- and C-terminal XhoI and BamHI restriction endonuclease sites, permitting direct cloning into the mammalian expression vectors pIRES2-EGFP and pEGFP-C1 (both from Clontech). The forward primer *Tad*NaC6-forward also contained the Kozak consensus sequence GCCGCCACC directly upstream of the start codon for efficient protein translation *in vitro*. The resulting expression vectors, named p*Tad*NaC6–IR–EGFP and pEGFP-*Tad*NaC6-C1, differ in that the former expresses the channel protein separate from soluble GFP (EGFP) through a bicistronic internal ribosome entry site, whereas the latter expresses the channel with an N-terminally fused EGFP. The S353A variant of *Tad*NaC6 was generated via site-directed mutagenesis using the p*Tad*NaC6–IR–EGFP vector as template and primers S353A-forward (5′-GCTATCCAACTGAAATCGCGCAATCATCACTTGGAAC) and S353A-reverse (5′-GTTCCAAGTGATGATTGCGCGATTTCAGTTGGATAGC). Chinese hamster ovary (CHO-K1) cells (Sigma) cells were cultured and transfected in 6-ml vented flasks incubated at 37 °C within a CO_2_ incubator, using Kaighn's modified Nutrient Mixture F-12 Ham's media (Sigma) supplemented with 10% fetal bovine serum (Wisent). For all *in vitro* expression experiments, 3 μg of plasmid DNA was transfected into CHO-K1 cells at 80–90% confluency with the transfection reagent PolyJet^TM^ (FroggaBio). Post-transfection, cells were washed two times with 4 ml of serum-free media and incubated for 24 h to ensure adequate channel protein expression.

To assess whether the *Tad*NaC6 channel protein was expressed as a full-length protein *in vitro*, Western blotting was performed on total protein lysates from CHO-K1 cells transfected with pEGFP-*Tad*NaC6-C1. Briefly, transfected and untransfected cells were lysed using Nonidet P-40 lysis buffer (150 mm NaCl, 1% w/v Nonidet P-40, 1 μg/ml protease inhibitor, 1 mm phenylmethylsulfonyl fluoride, 50 mm Tris-HCl, pH 8.0), and protein lysates were quantified using a Bradford assay. Aliquots containing 50 μg of total protein were combined with appropriate volumes of 4× NuPAGE LDS sample buffer (Invitrogen), loaded onto a denaturing 4–12% bis-tris polyacrylamide gel (Invitrogen), and electrophoresed at 150 mV in 1× NuPAGE MOPS running buffer (2.5 mm MOPS, 0.005% SDS, 0.05 mm EDTA, 2.5 mm Tris base, pH 7.7). Proteins were transferred onto 0.2-μm nitrocellulose membranes (GE Healthcare) under denaturing conditions using 1× NuPAGE transfer buffer (0.5 mm Bicine, 0.05 mm EDTA, 0.5 mm bis-tris, pH 7.2). The membrane was then incubated for 1 h at room temperature in 20 ml of TBST (137 mm NaCl, 2.7 mm KCl, 1% v/v Tween 20, 19 mm Tris base, pH 7.5) containing 5% w/v skim milk powder, and then for 6 h at 4 °C in TBST bearing 5% milk powder and monoclonal mouse anti-GFP IgG1κ antibody (Sigma) diluted to 1:1000. After three washes in TBST, the secondary antibody goat anti-mouse IgG peroxidase conjugate (Sigma) was applied at 1:4000 dilution for 2 h at room temperature, and the membrane was washed again three times in TBST and then imaged on an ImageQuant^TM^ LAS 500 imager using SuperSignal West Femto Maximum Sensitivity Substrate (Thermo Fisher Scientific).

### Patch-clamp electrophysiology

For electrophysiology, CHO cells transfected with the p*Tad*NaC6–IR–EGFP vector were trypsinized the following day and were plated onto glass coverslips in 35-mm culture dishes and incubated overnight at 37 °C in a CO_2_ incubator. On the day of recording, coverslips were transferred to new 35-mm culture dishes bearing ∼3 ml of appropriate extracellular recording solution. For the pH dose-response curve shown in Fig. 1, the external solution contained 140 mm NaCl, 4 mm KCl, 2 mm CaCl_2_, 1 mm MgCl_2_, 5 mm HEPES, 5 mm MES (pH 5–7.5 with HCl/NaOH), or 140 mm NaCl, 4 mm KCl, 2 mm CaCl_2_, 1 mm MgCl_2_, 5 mm HEPES, 5 mm CHES (pH 8 to 9 with HCl/NaOH). The intracellular solution consisted of 120 mm KCl, 2 mm MgCl_2_, 10 mm EGTA, 10 mm HEPES, pH 7.2, with KOH. All salts used in our experiments were purchased from Sigma and were of high purity (≥99%). Similar solutions were used for the low Ca^2+^ pH dose-response curve shown in Fig. 6, *D* and *E*; however, the external [Ca^2+^] was reduced to 0.1 mm. For the Ca^2+^ dose-response curve (Fig. 6, *B* and *C*), the external solution consisted of 60 mm NaCl, CaCl_2_ (1, 10, 100, 1000, 3000, and 30,000 μm), and 10 mm HEPES, pH 7.5, and the same internal solution as above was used. For the ion selectivity experiments (Fig. 5), the external contained 150 mm *X*Cl ion where *X* = Na, K, Li, or Cs, 10 mm TEA-Cl, and 10 mm HEPES, pH 7.4, with *X*OH, and the internal solution contained 150 mm NaCl, 10 mm EGTA, 10 mm TEA-Cl, and 10 mm HEPES, pH 7.2, with NaOH. Whole-cell patch-clamp recordings were obtained using an Axopatch 200B amplifier coupled to a Digidata 1550A digitizer, using the pClamp 10 software (Molecular Devices). Patch pipettes were pulled using a P-1000 micropipette puller (Sutter), from thick-walled borosilicate tubing (1.5 and 0.86 outer and inner diameter, respectively), to a resistance of between 2 and 5 megohms. Series resistance was not compensated, and we only used data for analysis where the patch had minimal access resistance and tight capacitive transients upon voltage steps. Recordings were sampled at 2000 Hz and filtered offline at 500 Hz using the pCLAMP software. For all experiments that required perfusion of external solutions, we employed a Valvelink8.2® gravity flow Teflon perfusion system (AutoMate Scientific, Berkeley, CA). IC_50_ values for proton, Ca^2+^, and diminazene dose-response curves were determined by fitting monophasic or biphasic dose-response curves over the data using the software package Origin 2016 (OriginLab). The relative permeability *P_X_*/*P*_Na_ determined for *Tad*NaC6 (Fig. 5), where *X* is a monovalent cation (Li^+^, Cs^+^, or K^+^), was determined using Equation 1,

(Eq. 1) $$\Delta {\text{E}}_{\text{Rev}}={\text{E}}_{\text{Rev},\text{X}}-{\text{E}}_{\text{Rev},\text{Na}}=\text{RT}/\text{zF}\times \ln{\text{P}}_{\text{x}}/{\text{P}}_{\text{Na}}$$

## Author contributions

W. E. and A. S. conceptualization; W. E. and A. S. data curation; W. E., C. L. S., and A. S. formal analysis; W. E., C. L. S., and A. S. investigation; W. E. and A. S. visualization; W. E., C. L. S., and A. S. writing-original draft; C. L. S. and A. S. resources; C. L. S. and A. S. funding acquisition; A. S. supervision; A. S. project administration.

## Supplementary Material

Supporting Information

## Footnotes

3 Y. Y. Wong, L. Phuong, W. Elkhatib, T. Piekut, and A. Senatore, manuscript under review.

## Footnotes

2 The abbreviations used are:

DEG/ENaC: degenerin/epithelial Na^+^ channel
ANOVA: analysis of variance
ASIC: acid-sensitive ion channel
EGFP: enhanced GFP
bis-tris: 2-[bis(2-hydroxyethyl)amino]-2-(hydroxymethyl)propane-1,3-diol
Bicine: *N*,*N*-bis(2-hydroxyethyl)glycine
CHES: 2-(cyclohexylamino)ethanesulfonic acid
RPK: ripped pocket
PPK: pickpocket
GMQ: 2-guanidine-4-methylquinazoline
MEC: mechanosensory protein
ACD-1: acid-sensitive channel, degenerin-like
TEA-Cl: tetraethylammonium chloride.
