# Supplementary material for: A Na+ leak channel cloned from Trichoplax adhaerens extends extracellular pH and Ca2+ sensing for the DEG/ENaC family close to the base of Metazoa
Source: J Biol Chem. 2019 Sep 15;294(44):16320–36. doi: 10.1074/jbc.RA119.010542 (PMC6827283; doi:10.1074/jbc.RA119.010542)
Supplement: Supporting Information [file supp_294_44_16320__index.html]

A Na+ leak channel cloned from Trichoplax adhaerens extends extracellular pH and Ca2+ sensing for the DEG/ENaC family close to the base of Metazoa — In vitro properties of a placozoan DEG/ENaC ion channel — A Na+ leak channel cloned from Trichoplax adhaerens extends extracellular pH and Ca2+ sensing for the DEG/ENaC family close to the base of Metazoa — In vitro properties of a placozoan DEG/ENaC ion channel — Supporting Information 

# A Na+ leak channel cloned from *Trichoplax adhaerens* extends extracellular pH and Ca2+ sensing for the DEG/ENaC family close to the base of Metazoa

## Supporting Information

- Supplementary Figures S1 and S2 - Supplementary figures S1 and S2
- Supplementary file 1 - Supplementary file 1 FASTA file of DEG/ENaC channel protein sequences
- Supplementary file 2 - Compressed file bearing PDB structures of DEG/ENaC channels described in the manuscript
