# Supplementary material for: A Na+ leak channel cloned from Trichoplax adhaerens extends extracellular pH and Ca2+ sensing for the DEG/ENaC family close to the base of Metazoa
Source: J Biol Chem. 2019 Sep 15;294(44):16320–36. doi: 10.1074/jbc.RA119.010542 (PMC6827283; doi:10.1074/jbc.RA119.010542)
Supplement: Supporting Information [file supp_RA119.010542_155050_1_supp_393504_pxmmx2.pdf]

A Na^+ leak channel cloned from *Trichoplax adhaerens* extends extracellular pH and Ca^{2+} sensing for the DEG/ENaC family close to the base of Metazoa

Wassim Elkhatib, Carolyn L. Smith and Adriano Senatore

Page S2: Supplementary Figure S1

Page S3: Supplementary Figure S2

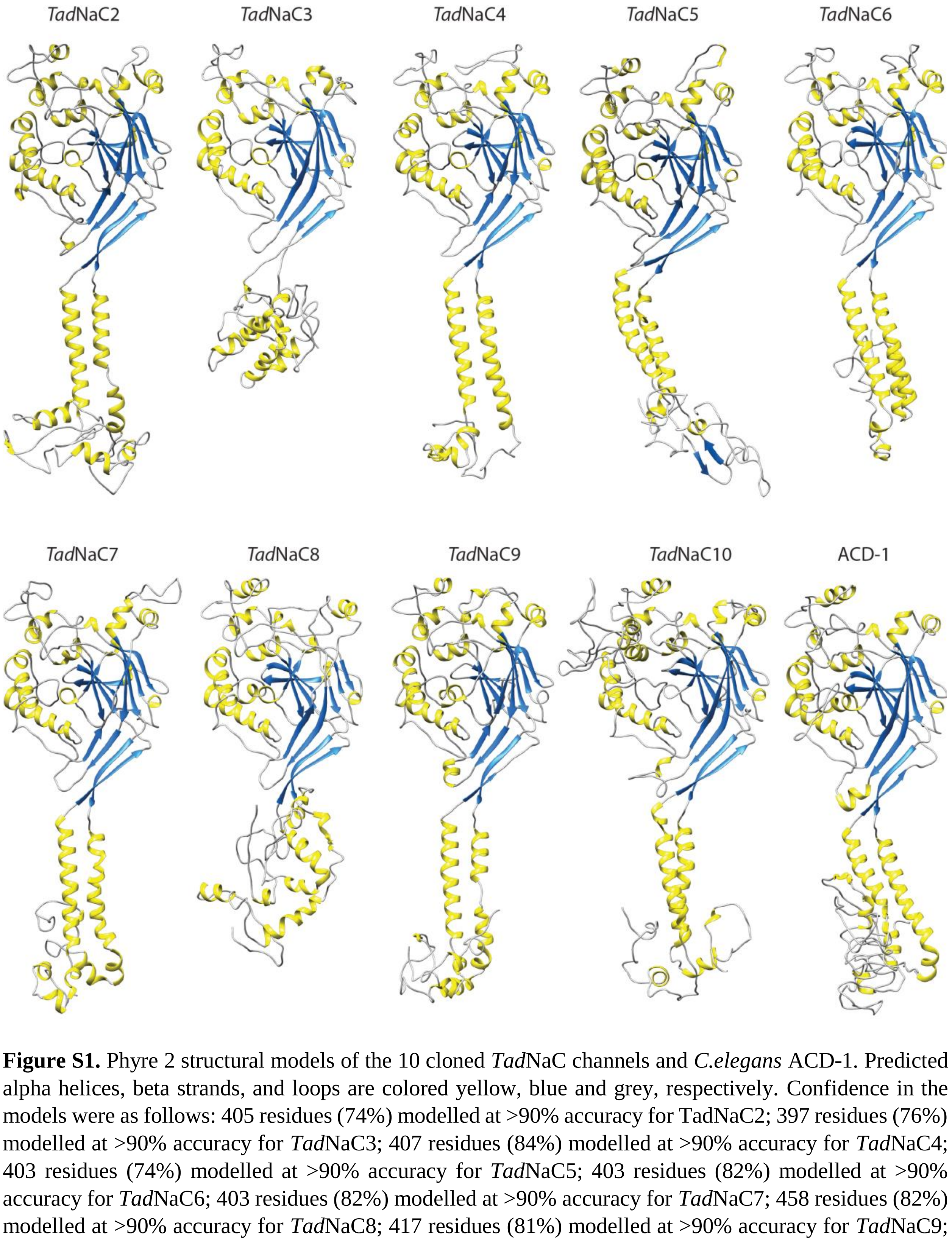

Figure S1. Phyre 2 structural models of the 10 cloned *Tad*NaC channels and *C.elegans* ACD-1. Predicted alpha helices, beta strands, and loops are colored yellow, blue and grey, respectively. Confidence in the models were as follows: 405 residues (74%) modelled at >90% accuracy for TadNaC2; 397 residues (76%) modelled at >90% accuracy for *Tad*NaC3; 407 residues (84%) modelled at >90% accuracy for *Tad*NaC4; 403 residues (74%) modelled at >90% accuracy for *Tad*NaC5; 403 residues (82%) modelled at >90% accuracy for *Tad*NaC6; 403 residues (82%) modelled at >90% accuracy for *Tad*NaC7; 458 residues (82%) modelled at >90% accuracy for *Tad*NaC8; 417 residues (81%) modelled at >90% accuracy for *Tad*NaC9; 566 residues (92%) modelled at >90% accuracy for *Tad*NaC10; and 438 residues (69%) modelled at >90% accuracy for ACD-1.

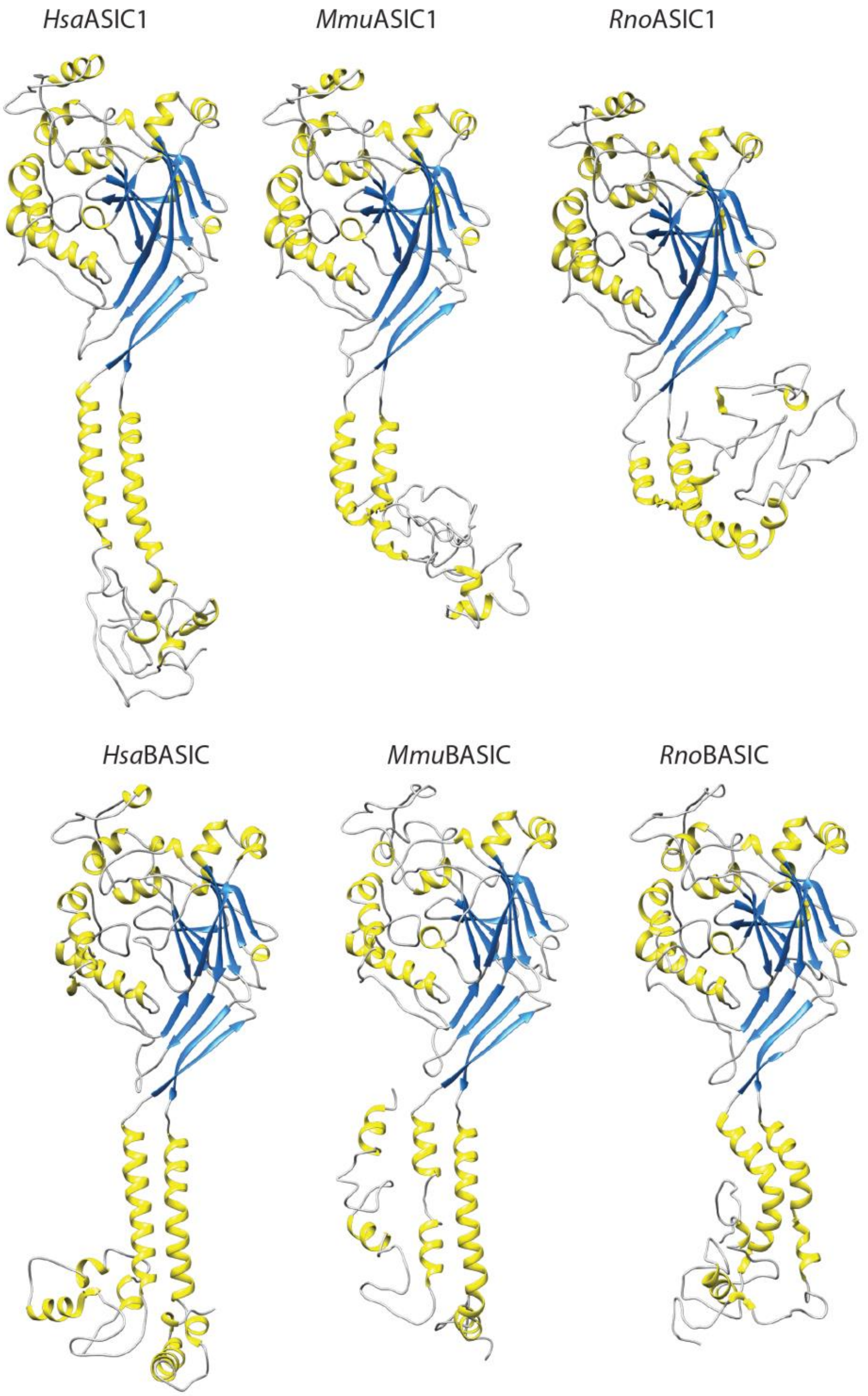

Figure S2. Phyre2 structural models of the human (*Hsa*), mouse (*Mmu*) and rat (*Rno*) ASIC1 and BASIC channels. Predicted alpha helices, beta strands, and loops are colored yellow, blue and grey, respectively. Confidence in the models were as follows: 417 residues (79%) modelled at >90% accuracy for *Hsa*ASIC1; 415 residues (79%) modelled at >90% accuracy for *Mmu*ASIC1; 415 residues (79%) modelled at >90% accuracy for *Rno*ASIC1; 409 residues (81%) modelled at >90% accuracy for HsaASIC5; 440 residues (89%) modelled at >90% accuracy for *Mmu*ASIC5; and 410 residues (83%) modelled at >90% accuracy for *Rno*ASIC5.
