# Supplementary material for: A Na+ leak channel cloned from Trichoplax adhaerens extends extracellular pH and Ca2+ sensing for the DEG/ENaC family close to the base of Metazoa
Source: J Biol Chem. 2019 Sep 15;294(44):16320–36. doi: 10.1074/jbc.RA119.010542 (PMC6827283; doi:10.1074/jbc.RA119.010542)
Supplement: Supporting Information [file supp_RA119.010542_155050_1_supp_391148_pxf4hm.pdf]

```
>hASIC1 NP_064423.2 acid-sensing ion channel 1 isoform a [Homo sapiens]
MELKAEEEEVGGVQPVSIQAFASSSTLHGLAHIFSYERLSLKRALWALCFLGSLAVLLCVCTERVQYYFH
YHHVTKLDEVAASQLTFPAVTLCNLNEFRFSQVSKNDLYHAGELLALLNNRYEIPDTQMADEKQLEILQD
KANFRSFKPKPFNMREFYDRAGHDIRDMLLSCHFRGEVCSAEDFKVVFTRYGKCYTFNSGRDGRPRLKTM
KGGTGNGLEIMLDIQQDEYLPVWGETDETSFEAGIKVQIHSQDEPPFIDQLGFGVAPGFQTFVACQEQRL
IYLPPPWGTCKAVTMDSDLDFFDSYSITACRIDCETRYLVENCNCRMVHMPGDAPYCTPEQYKECADPAL
DFLVEKDQEYCVCEMPCNLTRYGKELSMVKIPSKASAKYLAKKFNKSEQYIGENILVLDIFFEVLNYETI
EQKKAYEIAGLLGELLMTPVPFSCHGHGVAPYHPKAGCSLLSHEGPPPQRPFPKPCCLGDIGGQMGLFIG
ASILTVLELFDYAYEVIKHKLCRRGKCQKEAKRSSADKGVALSLDDVKRHNPCESLRGHPAGMTYAANIL
PHHPARGTFEDFTC

>hASIC2 NP_001085.2 acid-sensing ion channel 2 isoform MDEG1 [Homo sapiens]
MDLKESPSEGSLQPSSIQIFANTSTLHGIRHIFVYGPLTIRRVLWAVAFVGSLGLLLVESSERVSYYFSY
QHVTKVDEVVAQSLVFPAVTLCNLNGFRFSRLTTNDLYHAGELLALLDVNLQIPDPHLADPSVLEALRQK
ANFKHYKPKQFSMLEFLHRVGHDLKDMMLYCKFKGQECGHQDFTTVFTKYGKCYMFNSGEDGKPLLTTVK
GGTGNGLEIMLDIQQDEYLPIWGETEETTFEAGVKVQIHSQSEPPFIQELGFGVAPGFQTFVATQEQRLT
YLPPPWGECRSSEMGLDFFPVYSITACRIDCETRYIVENCNCRMVHMPGDAPFCTPEQHKECAEPALGLL
AEKDSNYCLCRTPCNLTRYNKELSMVKIPSKTSAKYLEKKFNKSEKYISENILVLDIFFEALNYETIEQK
KAYEVAALLGDIGGQMGLFIGASILTILELFDYIYELIKEKLLDLLGKEEDEGSHDENVSTCDTMPNHSE
TISHTVNVPLQTTLGTLEEIAC

>hASIC3 NP_004760.1 acid-sensing ion channel 3 isoform a [Homo sapiens]
MKPTSGPEEARRPASDIRVFASNCSMHGLGHVFGPGSLSLRRGMWAAAVVLSVATFLYQVAERVRYYREF
HHQTALDERESHRLIFPAVTLCNINPLRRSRLTPNDLHWAGSALLGLDPAEHAAFLRALGRPPAPPGFMP
SPTFDMAQLYARAGHSLDDMLLDCRFRGQPCGPENFTTIFTRMGKCYTFNSGADGAELLTTTRGGMGNGL
DIMLDVQQEEYLPVWRDNEETPFEVGIRVQIHSQEEPPIIDQLGLGVSPGYQTFVSCQQQQLSFLPPPWG
DCSSASLNPNYEPEPSDPLGSPSPSPSPPYTLMGCRLACETRYVARKCGCRMVYMPGDVPVCSPQQYKNC
AHPAIDAMLRKDSCACPNPCASTRYAKELSMVRIPSRAAARFLARKLNRSEAYIAENVLALDIFFEALNY
ETVEQKKAYEMSELLGDIGGQMGLFIGASLLTILEILDYLCEVFRDKVLGYFWNRQHSQRHSSTNLLQEG
LGSHRTQVPHLSLGPRPPTPPCAVTKTLSASHRTCYLVTQL

>hASIC4 NP_878267.2 acid-sensing ion channel 4 isoform 2 [Homo sapiens]
MLSGAAGAARRGGAALAPSLTRSLAGTHAGADSCAGADKGSHKETIEERDKRQQRQQRQRQHQGCGAAGS
GSDSPTSGPHPVPVLFPLALSLEEQPLPPLPLGRAPGLLAREGQGREALASPSSRGQMPIEIVCKIKFAE
EDAKPKEKEAGDEQSLLGAVAPGAAPRDLATFASTSTLHGLGRACGPGPHGLRRTLWALALLTSLAAFLY
QAAGLARGYLTRPHLVAMDPAAPAPVAGFPAVTLCNINRFRHSALSDADIFHLANLTGLPPKDRDGHRAA
GLRYPEPDMVDILNRTGHQLADMLKSCNFSGHHCSASNFSVVYTRYGKCYTFNADPRSSLPSRAGGMGSG
LEIMLDIQQEEYLPIWRETNETSFEAGIRVQIHSQEEPPYIHQLGFGVSPGFQTFVSCQEQRLTYLPQPW
GNCRAESELREPELQGYSAYSVSACRLRCEKEAVLQRCHCRMVHMPDSLGGGPEGPCFCPTPCNLTRYGK
EISMVRIPNRGSARYLARKYNRNETYIRENFLVLDVFFEALTSEAMEQRAAYGLSALLGDLGGQMGLFIG
ASILTLLEILDYIYEVSWDRLKRVWRRPKTPLRTSTGGISTLGLQELKEQSPCPSRGRVEGGGVSSLLPN
HHHPHGPPGGLFEDFAC

>hASIC5 NP_059115.1 acid-sensing ion channel 5 [Homo sapiens]
MEQTEKSKVYAENGLLEKIKLCLSKKPLPSPTERKKFDHDFAISTSFHGIHNIVQNRSKIRRVLWLVVVL
GSVSLVTWQIYIRLLNYFTWPTTTSIEVQYVEKMEFPAVTFCNLNRFQTDAVAKFGVIFFLWHIVSKVLH
LQEITANSTGSREATDFAASHQNFSIVEFIRNKGFYLNNSTLLDCEFFGKPCSPKDFAHVFTEYGNCFTF
NHGETLQAKRKVSVSGRGLSLLFNVNQEAFTDNPALGFVDAGIIFVIHSPKKVPQFDGLGLLSPVGMHAR
VTIRQVKTVHQEYPWGECNPNIKLQNFSSYSTSGCLKECKAQHIKKQCGCVPFLLPGYGIECDLQKYFSC
VSPVLDHIEFKDLCTVGTHNSSCPVSCEEIEYPATISYSSFPSQKALKYLSKKLNQSRKYIRENLVKIEI
NYSDLNYKITQQQKAVSVSELLADLGGQLGLFCGASLITIIEIIEYLFTNFYWICIFFLLKISEMTQWTP
PPQNHLGNKNRIEEC

>rASIC1 NP_077068.1 acid-sensing ion channel 1 [Rattus norvegicus]
MELKTEEEEVGGVQPVSIQAFASSSTLHGLAHIFSYERLSLKRALWALCFLGSLAVLLCVCTERVQYYFC
YHHVTKLDEVAASQLTFPAVTLCNLNEFRFSQVSKNDLYHAGELLALLNNRYEIPDTQMADEKQLEILQD
KANFRSFKPKPFNMREFYDRAGHDIRDMLLSCHFRGEACSAEDFKVVFTRYGKCYTFNSGQDGRPRLKTM
```

KGGTGNGLEIMLDIQQDEYLPVWGETDETSFEAGIKVQIHSQDEPPFIDQLGFGVAPGFQTFVSCQEQRL
IYLPSPWGTCNAVTMDSDFFDSYSITACRIDCETRYLVENCNCRMVHMPGDAPYCTPEQYKECADPALDF
LVEKDQEYCVCEMPCNLTRYGKELSMVKIPSKASAKYLAKKFNKSEQYIGENILVLDIFFEVLNYETIEQ
KKAYEIAGLLGDIGGQMGLFIGASILTVLELFDYAYEVIKHRLCRRGKCQKEAKRSSADKGVALSLDDVK
RHNPCESLRGHPAGMTYAANILPHHPARGTFEDFTC

>rASIC2 NP_001029186.1 acid-sensing ion channel 2 isoform MDEG1 [Rattus norvegicus]
MDLKESPSEGSLQPSSIQIFANTSTLHGIRHIFVYGPLTIRRVLWAVAFVGSLGLLLVESSERVSYYFSY
QHVTKVDEVVAQSLVFPAVTLCNLNGFRFSRLTTNDLYHAGELLALLDVNLQIPDPHLADPTVLEALRQK
ANFKHYKPKQFSMLEFLHRVGHDLKDMMLYCKFKGQECGHQDFTTVFTKYGKCYMFNSGEDGKPLLTTVK
GGTGNGLEIMLDIQQDEYLPIWGETEETTFEAGVKVQIHSQSEPPFIQELGFGVAPGFQTFVATQEQRLT
YLPPPWGECRSSEMGLDFFPVYSITACRIDCETRYIVENCNCRMVHMPGDAPFCTPEQHKECAEPALGLL
AEKDSNYCLCRTPCNLTRYNKELSMVKIPSKTSAKYLEKKFNKSEKYISENILVLDIFFEALNYETIEQK
KAYEVAALLGDIGGQMGLFIGASLLTILELFDYIYELIKEKLLDLLGKEEEEGSHDENMSTCDTMPNHSE
TISHTVNVPLQTALGTLEEIAC

>rASIC3 NP_775158.1 acid-sensing ion channel 3 [Rattus norvegicus]
MKPRSGLEEAQRRQASDIRVFASSCTMHGLGHIFGPGGLTLRRGLWATAVLLSLAAFLYQVAERVRYYGE
FHHKTTLDERESHQLTFPAVTLCNINPLRRSRLTPNDLHWAGTALLGLDPAEHAAYLRALGQPPAPPGFM
PSPTFDMAQLYARAGHSLEDMLLDCRYRGQPCGPENFTVIFTRMGQCYTFNSGAHGAELLTTPKGGAGNG
LEIMLDVQQEEYLPIWKDMEETPFEVGIRVQIHSQDEPPAIDQLGFGAAPGHQTFVSCQQQQLSFLPPPW
GDCNTASLDPDDFDPEPSDPLGSPRPRPSPPYSLIGCRLACESRYVARKCGCRMMHMPGNSPVCSPQQYK
DCASPALDAMLRKDTCVCPNPCATTRYAKELSMVRIPSRASARYLARKYNRSESYITENVLVLDIFFEAL
NYEAVEQKAAYEVSELLGDIGGQMGLFIGASLLTILEILDYLCEVFQDRVLGYFWNRRSAQKRSGNTLLQ
EELNGHRTHVPHLSLGPRPPTTPCAVTKTLSASHRTCYLVTRL

>rASIC4 NP_071570.2 acid-sensing ion channel 4 [Rattus norvegicus]
MPIEIVCKIKFAEEDAKPKEKEAGDEQSLLGAAQGPAAPRDLATFASTSTLHGLGRACGPGPHGLRRTLW
VLALLTSLAAFLYQAASLARGYLTRPHLVAMDPAAPAPVAGFPAVTLCNINRFRHSALSDADIFHLANLT
GLPPKDRDGHRAAGLRYPEPDMVDILNRTGHQLADMLKSCNFSGHHCSASNFSVVYTRYGKCYTFNADPQ
SSLPSRAGGMGSGLEIMLDIQQEEYLPIWRETNETSFEAGIRVQIHSQEEPPYIHQLGFGVSPGFQTFVS
CQEQRLTYLPQPWGNCRAESKLREPELQGYSAYSVSACRLRCEKEAVLQRCHCRMVHMPGNETICPPNIY
IECADHTLDSLGGGSEGPCFCPTPCNLTRYGKEISMVKIPNRGSARYLARKYNRNETYIRENFLVLDVFF
EALTSEAMEQRAAYGLSALLGDLGGQMGLFIGASILTLLEILDYIYEVSWDRLKRVWRRPKTPLRTSTGG
ISTLGLQELKEQSPCPNRGRAEGGGASNLLPNHHHPHGPPGSLFEDFAC

>rASIC5 NP_071563.1 acid-sensing ion channel 5 [Rattus norvegicus]
MEHTEKSKGPAEKGLLGKIRRYLSKRPLPSPTDRKKFDHDFAISTSFHGIHNIAQNQNKVRKVIWLSVVL
GSVSLLVWQIYSRLVNYFMWPTTTSIEVQYVEKIEFPAVTFCNLNRFQTEAVSRFGIIFFLWDIVSKVLR
LQEISGNNTGSPEALDFVASHRNFSITEFVKNNGFYLNHDTLVHCEFFGKTCDPKDFKHVFTEYGNCFTF
NYGENVQSKNKVSVSGRGLKLLLDVHQEEFTDNPVPGFADAGVIFVIHSPKKEPQFDGLGLSSPVGMHAR
VTIRQLKTIHQEYPWGECNPDIKLRNFTTYSTYGCLKECKAKHIQRLCGCLPFLLPGNGVECDLLKYYNC
VSPILDHIERKGLCTMGTHNSSCPVPCEETEYPATIAYSTFPSQRATKFLAKKLNQSQEYIRENLVNIEI
NYSDLNYKITQQQKAVSVPELLADVGGQLGLFCGASLITIIEIIEYLFTSFYWVFIFFLLKILEMIQRTS
PPQTV

>mASIC1 NP_033727.1 acid-sensing ion channel 1 isoform 1 [Mus musculus]
MELKTEEEEVGGVQPVSIQAFASSSTLHGLAHIFSYERLSLKRALWALCFLGSLAVLLCVCTERVQYYFC
YHHVTKLDEVAASQLTFPAVTLCNLNEFRFSQVSKNDLYHAGELLALLNNRYEIPDTQMADEKQLEILQD
KANFRSFKPKPFNMREFYDRAGHDIRDMLLSCHFRGEACSAEDFKVVFTRYGKCYTFNSGQDGRPRLKTM
KGGTGNGLEIMLDIQQDEYLPVWGETDETSFEAGIKVQIHSQDEPPFIDQLGFGVAPGFQTFVSCQEQRL
IYLPSPWGTCNAVTMDSDFFDSYSITACRIDCETRYLVENCNCRMVHMPGDAPYCTPEQYKECADPALDF
LVEKDQEYCVCEMPCNLTRYGKELSMVKIPSKASAKYLAKKFNKSEQYIGENILVLDIFFEVLNYETIEQ
KKAYEIAGLLGDIGGQMGLFIGASILTVLELFDYAYEVIKHRLCRRGKCQKEAKRNSADKGVALSLDDVK
RHNPCESLRGHPAGMTYAANILPHHPARGTFEDFTC

>mASIC2 NP_001029185.1 acid-sensing ion channel 2 isoform MDEG1 [Mus musculus]
MDLKESPSEGSLQPSSIQIFANTSTLHGIRHIFVYGPLTIRRVLWAVAFVGSLGLLLVESSERVSYYFSY
QHVTKVDEVVAQSLVFPAVTLCNLNGFRFSRLTTNDLYHAGELLALLDVNLQIPDPHLADPTVLEALRQK
ANFKHYKPKQFSMLEFLHRVGHDLKDMMLYCKFKGQECGHQDFTTVFTKYGKCYMFNSGEDGKPLLTTVK
GGTGNGLEIMLDIQQDEYLPIWGETEETTFEAGVKVQIHSQSEPPFIQELGFGVAPGFQTFVATQEQRLT
YLPPPWGECRSSEMGLDFFPVYSITACRIDCETRYIVENCNCRMVHMPGDAPFCTPEQHKECAEPALGLL
AEKDSNYCLCRTPCNLTRYNKELSMVKIPSKTSAKYLEKKFNKSEKYISENILVLDIFFEALNYETIEQK
KAYEVAALLGDIGGQMGLFIGASILTILELFDYIYELIKEKLLDLLGKEEEEGSHDENMSTCDTMPNHSE
TISHTVNVPLQTALGTLEEIAC

>mASIC3 NP_892045.2 acid-sensing ion channel 3 isoform 1 [Mus musculus]
MKPPSGLEEAQRRQASDIRVFANSCTMHGLGHIFGPGGLTLRRGLWATAVLLSLAAFLYQVAERVRYYGE
FHHKTTLDERESHQLTFPAVTLCNINPLRRSRLTPNDLHWAGTALLGLDPAEHAAYLRALGQPPAPPGFM
PSPTFDMAQLYARAGHSLEDMLLDCRYRGQPCGPENFTVIFTRMGQCYTFNSGAQGAELLTTPKGGAGNG
LEIMLDVQQEEYLPIWKDMEETPFEVGIRVQIHGQEEPPAIDQLGFGAAPGHQTFVSCQQQQLSFLPPPW
GDCNTASVDPDFDPEPSDPLGSPSSSPPYSLIGCRLACESRYVARKCGCRMMHMPGNSPVCSPQQYKDCA
SPALDAMLRKDTCVCPNPCATTRYAKELSMVRIPSRASARYLARKYNRSETYITENVLVLDIFFEALNYE
AVEQKAAYEVSELLGDIGGQMGLFIGASLLTILEILDYLCEVFQDRVLGYFWNRRSSQRRSGNTLLQEEL
NGHRTHVPHLSLGPRPPTAPSAVTKTLAASHRTCYLVTRL

>mASIC4 NP_898843.1 acid-sensing ion channel 4 [Mus musculus]
MPIEIVCKIKFAEEDAKPKEKEAGDEQSLLGAAQGPAAPRDLATFASTSTLHGLGRACGPGPHGLRRTLW
ALALLTSLAAFLYQAASLARGYLTRPHLVAMDPAAPAPVAGFPAVTLCNINRFRHSALSDADIFHLANLT
GLPPKDRDGHRAAGLRYPEPDMVDILNRTGHQLADMLKSCNFSGHHCSASNFSVVYTRYGKCYTFNADPQ
SSLPSRAGGMGSGLEIMLDIQQEEYLPIWRETNETSFEAGIRVQIHSQEEPPYIHQLGFGVSPGFQTFVS
CQEQRLTYLPQPWGNCRAESELREPELQGYSAYSVSACRLRCEKEAVLQRCHCRMVHMPGNETICPPNIY
IECADHTLDSLGGGSEGPCFCPTPCNLTRYGKEISMVKIPNRGSARYLARKYNRNETYIRENFLVLDVFF
EALTSEAMEQQAAYGLSALLGDLGGQMGLFIGASILTLLEILDYIYEVSWDRLKRVWRRPKTPLRTSTGG
ISTLGLQELKEQSPCPSRGRAEGGGASSLLPNHHHPHGPPGSLFEDFAC

>mASIC5 NP_067345.1 acid-sensing ion channel 5 [Mus musculus]
MEHTEKSQVHAEKGLLGKIKRYLSKRPLPSPTDRKKFDQDFAMSTSFHGIHNIAQNQNKVRKVIWLAVVL
GSVSLLVWQIYSRLVNYFTWPTTTSIEVQYVEKIEFPAVTLCNLNRFQTEAVSRFGIIFFLWDIVSKVLR
LQEISANNTGSPETLDFVTNHQNFSITEFVKNNGFYLNNDTLVHCEFFGKTCSPKDFKHVFTEYGNCFTF
NYGENIQNKNKVSVSGRGLKLLLDVHQEEFTDNPVPGFADAGVIFVIHSPKKEPQFDGLGLSSPVGMHAR
VTIRQLKTVHQEYPWGECNPNIKLRNFITYSTYGCLKECKARHIQRLCGCLPFLLPGNGVECDLLEYYNC
VSPILDHIERKGLCTMGTHNSSCPVSCEETEYPATVSYSTFPSQRATRFLAKKLNQSQEYIRENLVNIEI
NYSDLNYKITQQQKAVSVPELLADVGGQLGLFCGASLITIIEIIEYFFTNFYWVLIFFLLKILETIQRTS
PPQAV

>gASIC1 NP_001035557.1 acid-sensing ion channel 1 [Gallus gallus]
MMDLKVDEEEVDSGQPVSIQAFASSSTLHGISHIFSYERLSLKRVVWALCFMGSLALLALVCTNRIQYYF
LYPHVTKLDEVAATRLTFPAVTFCNLNEFRFSRVTKNDLYHAGELLALLNNRYEIPDTQTADEKQLEILQ
DKANFRNFKPKPFNMLEFYDRAGHDIREMLLSCFFRGEQCSPEDFKVVFTRYGKCYTFNAGQDGKPRLIT
MKGGTGNGLEIMLDIQQDEYLPVWGETDETSFEAGIKVQIHSQDEPPLIDQLGFGVAPGFQTFVSCQEQR
LIYLPPPWGDCKATTGDSEFYDTYSITACRIDCETRYLVENCNCRMVHMPGDAPYCTPEQYKECADPALD
FLVEKDNEYCVCEMPCNVTRYGKELSMVKIPSKASAKYLAKKYNKSEQYIGENILVLDIFFEALNYETIE
QKKAYEVAGLLGDIGGQMGLFIGASILTVLELFDYAYEVIKHRLCRRGKCRKNHKRNNTDKGVALSMDDV
KRHNPCESLRGHPAGMTYAANILPHHPARGTFEDFTC

>gASIC2 XP_418066.2 acid-sensing ion channel 2 [Gallus gallus]
MDLKDSTSEGSMGSLQPSSIQIFANTSTLHGIRHVFVYGPVTIRRLLWTLAFVGSLGLLLVESSDRVAFY
FSYQHVTKVDEVVANSLVFPAVTICNLNEFRFSRLTTNDLYHAGELLALLDVNLQIPNPHLADPAVLAIL
QEKANFKQYKPKVFNMQEFLARVGHDLKDMMLYCKFKGQECNHEDFKTVFTKYGKCYMFNSGEEGRPLLT
TVKGGTGNGLEIMLDIQQDEYLPIWGETEETTFEAGVKVQIHSQSEPPFVQELGFGVAPGFQTFVATQEQ
RLTYLPPPWGECRSSDMGLDFFPVYSITACRIDCETRYIVENCNCKMVHMPGDAPFCTPEQYKECAEPAL

GLLAEKDSNYCICRTPCNLTRYNKELSMVKIPSKTSAKYLEKKFNKSEKYISENILVLDIFFEALNYETI
EQKKAYEVAALLGDIGGQMGLFIGASILTILELFDYIYELIKEKLLDLLGKEEEEGSHDENVSTCDPMPN
HSETISHTVNVPLQATLGTLEEIAC

>gASIC3 XP_025003153.1 acid-sensing ion channel 3 [Gallus gallus]
MGADGDGGVPRRFPAAPIWGSRRVPRSPASRPRAALPSSGEDRRPQRGSRGRYAIRCRIRLRPGPLPAGH
SGSGAFRAPRAGMRAAVRRAEAPGPRRPRGGTPPGSGLCGCGTPRGAEGCGTPGPPHLRSRGGRGRSLCG
APGSPSAPRPGMGAEGRSCAPTPPVIAARGEGGRGRSPARPLPELPPRTPGGRRRTGEGWVGGGAPRPRC
GKAAGSAAAAASRWPRALRGRVWGRGRSLPRRWGGGGRQHEGRRGGRGGRGAAVAAAGVASSSSLHGISH
IFAYGAALRRALWGAFFLGALGLLLLVCAERVAYFLTYPHVTKLDEVAAHNLTFPAITICNLNEFRFSKI
TRNDMYHVGELLALLNERYEISNPQLAEPAVLAALRDKANFKNFKAKPFSMAEFYNRTGHDLADMLLQCS
FRGAGCSARNFSVIFTRLGKCYTFNSGQPGTELLTTLQGGAGNGLELMLNIQQEEYLPVWGDTDETSYEA
GVKVQIHSQQEPPFIDQLGFGVAPGFQTFVSCQQQRLVYLPPPWGDCKATPIESDFFTNYSLTACRLDCE
TRYLAENCNCRMVHMPGNANVCTPEQYKECADPALDFLVKKDSEYCACRTPCDTVRYGKELSMVKIPSKA
SARYLARKFNKTEQYIADNVLVLDIFFEALNYEMIEQKKAYEVAGLLGDIGGQMGLFIGASLLTILEIFD
YLYEVFRDKLIGFYKDKKRMRRGSSTTLEHPAVPGSPAATLPPRTPIGPCAATRTVSPSPRTCYLVTRL

>gASIC4 XP_001232417.5 acid-sensing ion channel 4 isoform X1 [Gallus gallus]
MPGQPAWGSVTLPQPPAGLGEEFSIPKSRLGKACVGTSPLLSLPSPVTAQEGACHIRLWEGAEQKAPGRE
TASGARSEAGHAGSVLTTAMPLPLSCCPDGEALAPAQGGFLRATRIPGLHYMGTRPQSCLRRLLWGLAFL
ASAGLLATGATDRLHHLLSRPVLTRARLTRVPQLRFPAVTLCNPNRARFLQLTKPDLYSVGQWLGLSRED
RSLVPELLAMLGDEQRRWLTRLANYSRFLPPRRSERTMQSFFHRLSHQIEDMLVECRFQGKRCGPQHFTP
VYTRYGKCYTFNGDRRNPRVTRQGGMGNGLEIMLDIQQEEYLPIWRETNETSFEAGIRVQIHSQDEPPYI
HQLGFGVSPGFQTFVSCQEQRLTYLPQPWGNCRASVQGEQMLPGYDTYSIAACRLQCEKEAVVRSCHCRM
VHMPGNESICSPNVYIECADHTLDAAVEDSQERCSCPTPCNLTRYGKEISMVRIPNKGSARYLARKYNKN
ETYIRENFLVLDIFFEALNYEAIEQKKAYDLAGLLGDIGGQMGLFIGASILTILEILDYIYEVIRDRVSR
VLRHSKPPLKKPSGSIATLGLEELKDQSPCETLGRHVEGTYNAGILPNHHHRHHYPHQGVFEDFAC

>gASIC5 XP_015140827.1 acid-sensing ion channel 5 isoform X1 [Gallus gallus]
MDQLGRFTQLDAKGKLEQIRLYLKKMLPRLEDRRKYRQEFASSTSFHGVYNIVQTQTRTRRVLWLLVVTG
CLGIVIWQICSRFNYYFSWPTTTTVVVQHVENVKFPAVTFCNLNRFQAHAVSNLQIIFFLWNIVSGIVQK
FAMEDKYFHELNGFLLGNQNFSIKEFTRENGFYLNGSTLLECEFFGKTCHPEDFEHIFTEYGNCFTFNYN
DLPARRVSLSGRGLHLLFDVQQEQFTDDPALGYTDAGITFVIHSPKEIPRFDGLGLLTPVGMHAQVSIQQ
LKSIIQEYPWGECKPDIKLQYQDTYSTNGCLKECKAWYIQDWCGCLPFILPGNGIECDLMKYYNCVYPAI
HDIEVKGLCTVGTHNSTCPAPCEETHYPTTVTYSSFGGENAIKYISAKLKKSPEYIRQNLVIIDIKYDDL
NYRITQQQKALTISELLADVGGQLGLFCGASMITIIEVLEYIFTNFFWMCLFLLLKAPEIPRWNNPSHDQ
PTHVEKNKGIQEC

>BbASICa XP_019621273.1 PREDICTED: acid-sensing ion channel 2-like isoform X1
[Branchiostoma belcheri]
MHVKLTCSVELDCCPCCDCGQCCCGSSTAGSEPRDAGSELGDGTDAGEQTYYRKQAQTISDFAAGSTLHG
LPHIFPDAPLSIRQVAWALAFIGSLSVLLYQCSDRVKFYFQYPHITKLDMVLAERLDFPAITICNMNMFR
WKQFTQNDLWHMGKGLNILDENNNLRCSEYATPGDMEALKNKANFDSFKPSPFSIMEFANRTGHQIESFL
LDCKWKNFTCGPEYFTPVFTRYGKCYTFNSGSPNQPVLKTLKGGIGNGVEFFLDVQQEDYMPAWGESDEV
TFEVGFKIQLHTQEEPPFIHELGFGVGPGMQYYVSTQEQRITYLPAPWGQCKAENDLTFDFAKYTTSACR
IDCETKFVVSQCGCKMVHMPGNFPICTPDIYVECADQALDFLVKSDNKKCVCDTPCNTTRYNLFMSHVKF
PSEQAVKYLARKYGKPEDYFRKNTLVLNVFFEALNYETIEQQKAYEVASLLGDIGGQMGLFIGASILTIL
ELFDYLYEVLKDKCTERRHQPRSESNAVSVNLEDCKRDNSRAPLS

>OdASICa GSOIDP00000722001
[http://oikoarrays.biology.uiowa.edu/Oiko/Downloads.html]
MSHKKKVLFRDEFKDESEIYIHDSIAKINSAEKSCVLRNGRKTTATASSLDSFRPASIHV
FASNVTMHGFYHIFARHHSLIRRFAWSLAFLASLTLLILQSSNRVNYYLERPHVTKLDEI
SAINLRFPVVTICNLNEFRFSQIRRNDMYHAGEFLGFLNEQRKLHPAAIPKEDIENPEQY
QAVYDRLAELSDFSGSFKPTADFSLFEFYNRTGIQLEELLLDCFYRGEKCGYKDFKTVFT
RYGKCYSFNHPDEEKNIRKTLKGGVDNGLELLLDVQQDEYMPMWKESDEITYEAGLKVQI

HSQDQPPFIHELGFGISPGRQTLVSVKEQRVYFLPPPWGLCNDGSNKTQHANFDKYSISA
CRITCETRTIVKECNCRMIHMPNLETDKDIPYCLPKQYQCADSQLNFLVAEDNVACVCDT
PCDVVRYSKEQSSLLLPSEQAIDYMSWKYNKTPPYVKRNFAKLNVYFEALNYETVEQKKA
YEVPGLLGDIGGQMGLFIGASILTILELFDYVYEVIKEKSWGKTLRKRKKEKEIATARAI
IEEYRQKNGKLLTDIIGTNRTFSISGVE

>SpASICb XP_780968.2 PREDICTED: acid-sensing ion channel 1
[Strongylocentrotus purpuratus]
MGCRSGRCHLRKWAADTSDLHGLKHIAGSGNLFRRLVWLCLFLTALGFCIYECYLVFVGFLSFNHVTQVD
VLYSTEVEFPAVTVCNINKYRESAFTEDDIKNVGVHLGIIDEDHNLLLPELYTDEFRDFIDSVNWTVVEE
DPDYNMTDFTIRTGHQKEDMILSCLWKEEPCEESDFQHKLTHLGNCYSFNLQGANSDEADWKHSYAAGAA
NGLQLILNMETPEYTPTNDLDGGAMDAGLRIMFHYPTEPPYPKELGFAVAAGDHSFISMRHEKITSLPDP
YSECESDGADIITHFDHYSLQACRIECETLVVVAECGCRLPEMPGDDDVCGPADLHECAHPTLVEFITGN
LDSAESCECFSPCEVENYPFTLSTSRLRLTYLEALFANTSTNFTAAYIGENIAVVSMYYEALNFETIDML
PEYTVATLLAVLGGNLGLFLGASFLTLAQLGEYCFDEVVGWCICTPKKEDDDKNDGGNKVGHVPTVTAMD
AWGAQKL

>ApASICb gbr.173.6.t1 [Acanthaster planci;
https://marinegenomics.oist.jp/acornworm/gallery]
MGCYKRCYLRQWALTDTDLHGVKHIAGEGGILRRLIWAACFLAALVAFLHQATLTLIHFVEKHHVTKVDISYRKKLD
FPAVTVCNFNKYRESALTDRDIRNVGYHLGIVDEDHNLINPYLYTEEFRRKMAAVDWSVHDIDDEYNMTEFTNRTGH
QLDEMIVECSWRDEPCSPDDFHHIFSHLGNCYTFNHVALATERHSSISAGAANGLKLTLNIQEEEYTPSNDLHGAAE
DAGIKWMLHHPSEPPYVKELGFGAGPGDHTFVAVRHEEVESLPSPYTPCMETSAGFLDHFDHYSLQACRIECETEVV
VQRCGCRLVEQPGNAPVCNPAETHECAQAALVHAVAGHGDQACDCSSPCSVESYPFTTTNVRLRAKYIERIYSNTTH
NFSADYIQNNLVLLSIYYEALNAEVIEQLPEMTVPSLLAALGGNFGLFLGASVLTIVELLEYVFDELTASCTRRKTS
GRISNAREILTVRAAPISVIDTEKELEHGRQWNR

>PfASICb pfl_40v0_9_20150316_1g14838.t1 [Ptychodera flava;
https://marinegenomics.oist.jp/acornworm/gallery]
MEMKDNNYDDANSRKNNNVSPDSISAAIARQSYIDSPETPGVDIDSTPSGRIRIWAAQISDIHGVKHIVGERSRLRK
LLWALVVLASFGILLQQCIKAAINYGEFHHVTKVDVEYVQHMPFPAITICNFNKYRKSAITPADMVHVGKPLGLMDE
DGNLNSDLFSEEFVRQMKDLDWDSEEKKRFNYTEFTYRVGSQVKETIVECTWNGHKCTEHDFVKVFTHYGICFAFNK
YHRDTEARHAGKPGADNGLRVVLNAQTSEHLPTADLEDSFINVGFKLMIHPPTEPPYPKELGFAVGPGSHIFLAITR
QEIKRLSKPYGECDMKSVGSKYFDHYSMSACRIECETALLLEMCGCRLVEQPGNGPVCTPKIVKECAHVKLLEYIEG
HIEFDCPCHIPCDSEVYSVTPSSSRLKPDRSGKSPAMSNYTQEYIDSNVLVLTIFYEELNFETITQLPETSLVGLLG
QLGGNMGLFLGASILTLIQIIEYFVDECIHCFRPMAPKKPKRTYRGEDKDVNTPLSVQHWQGSHPVRNTTLMIVQHD
CEIVSVEHFIPLYEILTGSANTGLFVFPRCLRTRAANMLRFTGPVWRQRLSFELLRNAENQFITFLRHDAWYSCLTY
KMSSKLVR

>TadNaC1 XP_002114386.1 hypothetical protein TRIADDRAFT_58138 [Trichoplax
adhaerens]
MNEELNEGSEKPITSSLYTKNFKVKTGKEFKDDLLTPKDQEDYSVFEKIELTTPSSPNAKDSKFEIEKEY
QYHQEDRSVFKAVKQPIPSLSCIESSKVKRDKVFENVLLTPGDDQEDYPAVEKVKHPMLSLACAESSKVK
ADKEFEDIILTPYDDNDDSSVFEEANQTTVSASYAENLHISTKDKFEFSDDSNYDDRFALQTSCNGIIRI
FGRGGRVRHAVWFLLTLTMTILCIITCVQRYDYLFTYPTNTAINYTVSKKLKFPAVSVCNFNRFRFSSLE
YGDWHRIGYLVNLFTTTDNNQIFTGLNGKTGKEWNDYLKNISFELYDNITFDITQFLNVKSNQAEVFIKH
CTWNDGRQPCSIENFTRIYTDYGSCFTFNAGVDAPILYQKRPGSRYGLKLILNIEEEEYTHLNPDPDIGI
KFRVHNQFEPPDINAEGIAVPPGYHAYTKLLYTESDFLKPPWGNCGQKKLKYFKSYSRASCQLECLADSY
RRRCDCRTPYMPGSSPICSPEHIKKCVTKYLGLSTPENFTCNCPNDCRIKSFNPHVTYAEIPLRQTSRFA
AHRYGLNELEIDFLKYNNISMGEYIRDNYVFLDLFYDDLSYTTFKEKKAYDVNQFISDIGGQLGLFLGGS
FLTFWEIFEWSQIKSFLVIRKIIHEYKKGRRRTRRRFNSTPEDTERLL

>TadNaC2 MK547543
MDTHPWQRPRSKSINLEKKFAERTTCHGLGHIVDQDVPKVRRYLWSIVTLAASVGCMIGCIQLLQYVLSF
PTNIDIEIIHQDSLIFPAVTICNFNQLTKTALSEEDNRHLQTFLRIYHRKGAINKKDLELATSYFRNKTG
VDFHLQNLTQDLGHRKDDIIVSCLWDDQVCGPENFTTIFTIYGNCYTFNSGAKKEGMLSQRGKGSAHGLR

LVLNIEQYKYSGKLSYGSPDAGIRFAVHSTADLPEMDAEGMSIPPGMHAYASIPGADVIEGLPKPWGQCG
SQKLKYFDHYSVSSCRREKEIDFILQRCGCVEPHHARNLTPCSPEIMLECVLPLMSSSNPVSVGINASVC
PVACVQTEYNVEVSYALIPSQVVVNDISDQYNVTKIIENARNQRLNLTMSKLEFIRENFAFLDVYYKDLY
LFKTIQKQASGFVAFLSDIGGQLGLFVGGSFLTMFEFFEYIYDKCFQQTKRSKREISRRMQSIREKRRPE
SMASSTSVRGNLSTNVHNFKVNESISSHNVRSNGIKSKFRRSNSEHLANIRISPSSID

>TadNaC3 MK547544
MDRNFQQRPRKPSVDFSTITTITTSTKDQSQDSDFFSQRQSPLNISYQHEEDFAKRTSCHGISHVFDDET
PKLRRLSWVFLTLAMATICIIQCADRMIYLISNPTKIVIKNEIPPRIRFPTVTICNFNRFRNSTINESNH
YRLQYLLRLMNAPQAPSKRSEFSFRNDLVNYSRNTNDDLDLLSKVGIQRQILIRYCNWDNQPHSCSAENF
TLTYTKYGNCFLFNGQSADNEALYQKRIGRSHGLRLAFNVEADQYTTMNPEPDVGIKFRIHEPDEPADIE
AQGVAIPPGYHAYVRLKYNEAKFLRKPIGKCDSRPLQYYDRYTRSGCKLECKTNKSIEVCGCRALYMPGS
APFCTAHQINQCMTNNIEEINNSSCYCPTACLWTSYDPAVSYSMTPTETITSEEAPLFGLNLDEVDANRV
LNSSISRYMRKNYIFLDIFFERLRYNTAIQELGYTFNAFLSDIGGQLGLFIGASVLTMLEIGEYFLTKLK
NLCKIIKRPTSQNEYEIAPASPNTTFEENPIPV

>TadNaC4 MK547545
MAKEDLSMEEKFASTTSCHGISHVYQNDSGRLPRMIWLILTIAATAVCISQCIIIIYDASQLPTRITFSD
VSANSTVFPSITICNTDNSPRDALPESDLKYLSILLHTQSTENAYNETQVQEAARYFIKKYGDKFDYENY
IRTSSAKLENMLLRCQWMNRPCSLSDFTSIVTDYGNCFTFNPGTKDLPLKNQTVPGEVYGLRLAFNIGQY
KYYPDLLDRNRPDAGIRFTIHYHKEPPNLLAKSIIAPVGSHTYVSFTRTYHKKLEKPWGECGSRKLLFHE
FYSHVACIDEVSAIYASTLCNCSIIGAFGPYGVCDSVKFITCIVPILAKARVQANENIAACPVACETYTY
PTVISYGNLALIPISSLITNYLNVSGIIRHAQSLDWIKDPNYSTANFVRDNILYLDIYYSDLHTNAIEQK
RATGFAEVLSNIGGQMGLFIGASIITIAEILQYLIRIFYNKSFNKPADKSKEDLNLEQNRKSSSV

>TadNaC5 MK547546
MTTNPSDDSDDSNSLASFDLEEHFANVTSCHGMIHIFDHKTSFLRRYVWGIATFAAFTACIIGCINLLHN
LLSHPTNISVKIHHTNKMLFPTVTLCNFNQFSRTLISHKDIRHLDTVLSAYNHDHTIPKEDLKEAEDYFK
SKIHGHFDFSQYNQELGLQKEDLILSCTWNGEKCGSKNFSRVFTSYGNCYTFNGGSANHPLLNQHGRGAA
HGLSLILNIEQYKYTPELMVGAPNVGIRCSIHYYKSLPRMESQGIAIPPGAHAYAAIPGTEVTNYQKKPW
GQCGEKKLKYYKYYSMSACLHEDETLFAETTCQCRDPRLPGNEKACTPIQMLECLIPAMSRYREQDRNLS
SCPEVCERVEYVPQVSYAKIPAKIMAEEIASKYNLHQVHKKAIIEGGIVDNSTSVKQFIRDNLVFLDIFF
KNLYNTTTTQARDASFSQFLSNVGGQIGLFIGGSFLTLLEIVEYIFDKLAQQRSRYRHKTSQIEKAEWKK
IYASSMIVPKTSYHNGNANHPPPYERTLVQMDEYDSTEGFLNGPKYVHVHKQCKE

>TadNaC6 MK547547
MMAKGKIDHDERFATTTSYHGVAHIYDSNNSKKTKSIWIILVILATAICISQCVIIIYNASLLPTRMVIR
KRLMNSSVFPSVTICNTNDFDYTGLPANDLNHLSSIVNAIYGFTPASAADDAIQYFVRKHGDDFQIDNYT
RMAGHKLKNMLLSCTWMGEPCTVNDFTNIISNGGSCFTFNPGTNAIPLKNQTVSGNINGLRLILNVEQYK
YYSPLFSPQAPDAGIRFTINYYKQPPNFISKPYYAPTGFHTYVPITLHRDKRLTKPWGECGELLLKDHSY
YSRDACLTEYASELAALTCNCSSSGQTATNSCNGAKFLTCIIPSSFVIRMSLSQNLSVCPIACETYSYPT
EISQSSLGTFAFSRVLDSVINISTILGKAKDEHWIPPSLPYSVSEFIRDNIVYLDIYYSDLRVTETEQQE
DTGFSKVLSEIGGQLGLCIGASVITLCEIIQYLIGKFFTSEKKTNLNKRQNTTSPLFYNRNSPDADATNP
DM

>TadNaC7 MK547548
MDEEKISEEERFATTTSYHGWAHIYDGNNGRMTKIIWMILVILATAACISQCIIIIYNASKLPTRMVIRK
QLMNTSIFPSVTLCNTNDYDRSALPAADLYHQSALVKAIYGSGDPNKTNAAINYFDKKFGGNFQFENLTR
VAGHKLSNMLLSCTWMEQPCYATDFVNIITDGGSCFTFNPGTGNLSLKNETVSGNSNGLRLILNVEQYKY
YSGIFALEQPDAGIRFTTHYYKQTPNFISKSYYAPTGFHTYVPITLQHDKRLKKPWGQCGEEILVYNSFY
SRDACLSEYAGRLASYICNCTFEPQIASNPCNGTQFLTCITPLASKFRQDLSQNLSICPIACETYSYPTE
ISQSALAALAFASSLDPLINVSGIIANAKANNWIAPNQSYFAADFIRDNIVYLDIYYSDLHLTQTLQEED

TGFSKIISELGGQLGICIGASALTICEIVQYLIKKYFASNKKSKSDKSSTNTISLGHDKKPFETTTVTNI
DL

>TadNaC8 MK547549
MSQSSDHDSNKTASDESSTDAHPNSQKVPILSHQDEVDQDNPEPRNRFDLDFRWRPSIEHDENFPFSASF
HGIEHIYEGRYGTRKILWILLVAATMIACFVFIFIQIAHYSAFHTTTKSTLVYEKQLAFPAVTICNYNSF
RRSAVTANDLIHMAYLVKAYRLNQGVVSDFIGEKERQKLINYWKKYDATHAKKFNYQLFVERVGYHASQM
IKSCHFRGLKCGPKNFSNVLTSYGNCITFNGPKLESNPKLYQKNPGAHQGLELLINIQEYEYTGSWHSDR
PDIGIKFVIHERHYPPDVTSQGKAVGPGSHAYASVKYKTISNLPSPYGHCGSKKLAFYKKYTYAGCQISC
KTEYVQKKCGCRAPDMPGQNIIPVCSPQKMIECVSPNLEKLITINDKVCICPIPCHIVHFDTTISYAKIP
NPQMAKDLTEKINKTAFQIETHGLVDPDIDPTLYISQNYILLNVFFDDLYYEKTVSTPVYTFTSLLGNIG
GQLGLFVGASVLTLVEIIEFGFYRSRGVIRRSDWKQNLRKSISRSREVTATEEKEPLCSVENGDTQLSK

>TadNaC9 MK547550
METANNDIQSTNLEKDFANATSYHGFGQFYKPQTTHYRRYFWMLLTVVATSACILQSSRIVIAAFQYPTK
ITSKVRYSNSSVFPAVTICNSNYLDKTKMDPDILAVVNSVFSNYNRSALDLVNLGLTPEIIAARFGQHAD
YGFFARAFGFKLKDIILECTYHNFNCLNSFTEVVEPNFGLCYQFNAGILQRGGNGNGGNNSSFHRQVGRG
PSFGLQLVLDAEQYKYSTLDFFYRYQAGFIIAIHDQFETSSMTINEISVGVGRYTSISLHQTLIEKYLKP
RWGECDDQELAFYRKYTREACKQEKESLYVMNLCKCRFKEVSESTVNYTICTTFDTITCVIPQLDLASRN
YRISNSCKIACARKLFPKTISSSSIGTLAYGNILNKRLDLSTKLADMQGLGLLPSSYTVQDYIRDNIVQL
DVFFSDLAHTTVQQEQDITPEEVLSNIGGQLGLFIGVSMLTVCEIVFYIFDKMQYWPQRRNRDNRQTTPV
DFEMKKSKVNNNANTIGVMGYGAET

>TadNaC10 MK547551
MPTKQLKVMSQHNGGISVRKKRLLAITVSRGFEDCGAQGISNMARAQTTQARILWAILTIAAISLCTVMA
VDLIQKYYRFEYDVTLKISFKPQLDFPVITICNLNPMLRSEMIKRPIFKPLYGRDFESVNTSQTSPLVNN
SSLSNNTVGQSDSNSSTPLPTNEVPQDATAAAAATTVAANSQGGTAAGSTKESGKTATPTGTKTSLPRVK
KSLSNDVNENIDRPTPPSGVQFENLDKSHENYHLFSEISAELFNRLTDQRMYELGTQASNFIAKCTFKQK
PCAANNFSISFNYLYGNCFSFNTGNSRGKRIESVNYPGPLFGLQLYLDINKNEYISREVPTAGVRIAVTP
QGVRPNPEDEGFDVPPGALTSIGLKMRNISRLSRPYNKEGCLKHPPNNKTLNIYENGSDRYSYKGCIKSC
IASLQNKTCGCIAARYSFTSSTKGLKVCSLKNETEINCQSRLQNRFIAGKINCGCVRACNEQSFEATTSQ
AQFPSEVNLDNNNDLLEYFKRDLTKFQLTRKTVTPFLRENLVAVNIYYEELNFETIEQTPRYSEIDLASD
IGGVLGLWIGISVLTVFEFMEILVDSLLIIFGKEKIASRRNTITEFKAQLQASGLA

>TadNaC11 XP_002114391.1 hypothetical protein TRIADDRAFT_58144 [Trichoplax adhaerens]
MGSKSENPRKVSNGQTDIVSFDLEENFATSTTCHGVAHIFETKGSRRSMWFGITLVSTIACLVQCCIIVYDASLYPT
RVNIKVKYNTQSLFPSVTLCNTNLIASIDNTREELKYAMLLYRHYYAKNQITHQELQSAENYFNRVYGKNFSLEEYV
ARYRFKAEDMIVSCQWGEEPCGSANFSQVITDYGTCYTFNSGQKGYPLLYQTISGSSHGLRLAINVQQYVYPTLPLS
FLTPDAGIRLSVHHYNEIANMGSRGVFVPPGMHGYIAITDTHVLSNLGPPWGECGQKNLQYFRYYSRSACRREFEAN
LAAQQCNCSYQQATVNTENKKTLNSFCPYPCQQTEYPIILSYAGIATNAIIDSSSHNGKLANLLDSVKNDPSKYPNY
TSEAFIRENLIYLDVYFRELITSTTTESKATGYAQVLSDVGGQLGLFIGASIITLCEIITYLCDRCERKKREKREMR
IHRQSQMIRDIMQVGVEKKDLQTETKPNKNNDFVFNTSEDGQETSSTNPNV

>HyNaC9 NP_001296667.1 acid-sensing ion channel 1-like [Hydra vulgaris]
MSEEDEKKIKSPHEQRNDRVKEHVAHLIKNVSFHGLSYVADKRNNYFRRAIWFLITVGAFIYAVEKVYES
TVNYFSYPFKTARMKIYVNELNFPAVSFCNLNDFLFSKLNGTKLDESILYPDDPEKNNVSEIEISNITSD
ATIRLDQMLVDCEFEGKKCTHENFTDFWTMQGELCFTFNSGKNSHSLLKVSGVGVLRSLKLTINVQHYEY
YRDEMAGGIHLMIHGQDEEPVKMQGQIVSPGYSFYVKVEKKTIMNLEKPYKTECGTVKLKYFDRYSMHTC
WLEQLTDYVNKMCHCKDFFMPGNIPYCSLPELQNCTWIEWAKFNKDKMYKCPLPCKIDLYGVSLSRALFP
TTQYSSILAEQFRKQPHVLSIVHNITDELLFMRDNLLRFIIYYDDLSYEVLEQKPSYETLVWLGDIGGQI
GLFIGAGVMSYFEFLDCLAIVIYTRFFQKFTSS

```
>HyNaC6 CDG50527.1 Hydra sodium channel 6 [Hydra vulgaris]
MRVNLKARMRKIVQERLTVKQIFKRYVESSTLHGFCYVCGDTFLVRRVLWALLMILGAIYFIIKLRYGIE
EYLNYPFSTLSTVDYVHELFFPAMSLCATNSYLASMVSKNQLKLLYDEGRLPLDNNQTNPSYNMSGNELV
KAIQESSLSIESMLAFCDWIQQDTNNPDIPPNPCGPQNFTTYLNYKGEQCYTLNSGLEGHKLLKVDTVGL
TYGYELIFDLQTNKVIKNNQFSGMRVVIHNQDVPPQLADGFIIPPGFKTFVKMGTVQSKSLPPPYSTECG
TKKLKYYNTYSQRFCLLETLTDFTGKLCGCRDVFMPENGLPFCSLKELYSCMYPAKESFSEFTMRKECPA
DCEERTYPYELSEARFIHNPPIGLSIQGLANLQHKKHLEEELYLSKLAQSMTSEELDAYVEDNIVSVIFF
FGDTRIDYNEQEATNDFFQFLGNMGGEFGLMLGASLLTFVEFVDLFIFLIYHQMLRLHTLKKVPDIFGRK
RSRINEYIKKREKTRV

>HyNaC2 CAL36110.1 hydra Na channel 2 [Hydra vulgaris]
MKARFARRMEKIVEERMTVKQIFNRYVQTSTLHGFRFIFMDTFIVRRVLWTILTLTMATIFFKELRNSIN
LFYEYPFTTTSTIQYEPSLTFPAISVCNLNHFLLSKIKKSKLKPLYDQGRLPFDNNLENPGFDIQGEELY
SILKTSSQSIDEIFLSCEWKSRDTAKNGVPNPCKPNNFTVYSGLYGQSCYTFNPGVSGYPLLSLSETGVN
MGFKLELDLKSKDSLQGIQEIGAIVIVHHQQETPVLQAGFVVSPGFQTFVEIKVRQTENLPPPYATKCGS
KPLKNYQIYRQSSCFLEQLGDAIETKCKCKSSFMAGRNIPYCSLRQTVTCLMPTIYDFDRKTNNNCPVDC
ETIQYLSSLSYARFISNVTYLSKNAEQSSYIRKLKNSMSPKKLQKYIEENIVAVQFFYQEMKKEKVKQEP
SYDFYKLIGDVGGQLGLLLGASVLTLVEFVDLFIFTLYHQLLRLSKKKS

>HyNaC7 CDG50528.1 Hydra sodium channel 7 [Hydra vulgaris]
MRIRLKERMRKIVQERLTVKQIFKRYIESSTLHGFCYVCMDTFLGRRLIWAVLMILGAIYFIFKLRYGIK
EYFDYPFSTLSTVEYVDDLLFPAVSVCATNSYIASQVYTNQLNTMYKEGRLPLDNNQSIPEYNIPGDELV
KTLKNSSLTIESLLKYCDWIMQDTSHPLVTPNNCGALNFTSYFNYKGEQCHTLNSGAKGHELLKVSDVGI
SHGYELVFDLQTNEVIKNYQLSGMRIVIHDQVFPPQLVDGFFISPGFKTYIKLGITQSQSLPPPYSTECG
QKKLKYYAIYSQRLCLLETLTDFTGDLCGCRDVFMPENGLPFCSLQELYSCMYPAKESFSEFTMRKECPS
DCEERTFSYELSEARYLHNPPIGLSLSRLDKLQLDKSLPSEAHLSKLAKSLTPKELDAYIESNIISVILF
FGDTRIDYHEQEATNDFFQFLGNMGGEFGLMLGASLLTFVEFIDLFIVLLYHQMLRLYNLRKIPDIFASK
RNRNKEKTKKCENV

>HyNaC11 NP_001296594.1 acid-sensing ion channel 1-like [Hydra vulgaris]
MLNFKDIAQITVEAIQETNEVTNKDEKNIQTIQDLRNKKIREHISYMIDNSSFHGLSYIFDKRHSVRRTI
WFFITIAAFAYAMQKVYESTMNYFSYPFYTVRMRMYVNQIDFPAISFCNLNDIKFSAMNGTIVDDAVVTQ
NHEANITGEEYRSYNQAARHTLNEMLVDCDFEGKKCSHKNFTEFSWMQGESCFTFNSGKPPHTLLKVKGA
GINRSLKLTINVQHYDYYRDKMDSGIRLILHGQDETPVKMSGLTVPPGFTTYIQIEKKTIINLEAPYKTK
CGSVKLKYFDSYSMHTCWLEQLTDYVYKTCNCKDYFMPGDIPICSFDLLYNCAWPEWETFDKQKLYQCPL
PCKIDSYEVSLSRALFPTGLYASSLANDLRKYQQVPIALKSKTDELIFMRENLLRLVIYYDDLAYELVEQ
KPSYNTLLWLGDVGGQIGLFIGAGVMSYFEFIDCLAMVIYTRFFEKISLKNPTTV

>HyNaC12 NP_001296710.1 amiloride-sensitive sodium channel subunit alpha-like
[Hydra vulgaris]
MDYLNLLERIRSSKVRRINKTSTDKEFSVYDNLSIRENFQDKMKTIRTKLHSSVTSAHGCLMIFKPKTKL
GKLMWFFVILCCFFFCIVNLVKITRDYSSENPEVKILELYEKSPTFPVVVICNENALNSSIKEHIISLTG
KKLDQFDSNLKKYITHKNFNSRLFNEFGNRLNSTLVSCTIGVDDCSTLLSWYQIWHLEYGLCFAFNSGFN
IEGNEVTTRRVFLPGYSHGLNLNLKLEKNEYNDKSKTTALRVFITHQGEYLFPPNEDLLLTSGFYYSISF
QKRLMNREYGSNKMCRKDKIIKLSNFSLSQPLITKYTQNFCNLNCLSEEIARECMCVEYDRPVLEQHAKI
PKCNVDASTQSCIKQQYSYWKSGSSECLQKCKLECFEVKYEPRIHLQKYCSNDLCEGDLKLSVNFRSFNY
FFYQVHINHVLAEFLGHAGGIITFLTGFSIISFIEASFITMQLVITILYMRCKSFKKSCDKTITSRKSFV
TCQASLEV

>HyNaC8 NP_001296668.1 acid-sensing ion channel 5-like [Hydra vulgaris]
MLELKCEISKRRRKRYQNAISYLPGANHDTVDELRNQSINDYLSDMAVNSSFHGINYICDSTYKVRRIIW
IVVTLTAMLYAMREVYESTRKYLNYPVSTVRMKIYVDDLEFPAVSLCNLNDVRMSIVNGTSFDNALIDQN
AQNISADDALMIAREARHNLEDMLLECKFNGRSCSAKNFSEFNWMQGDRCFTINSGKPGHSRLSVKGTGI
KRNLELILNLQHYEYYRDEMESGIHFILHSQEETPVRMRGPIVSPGFTTYFRINKIKTKNLKYPYKTRCG
SLNLKFFKGYSKQLCWLDQLTDYVNSKCGCKDFFMPGNISICTFSTAFGCMWPAWEEFEKKKISNCPLPC
DADTYFGQTVSRALFPSNEYKKSFIKNLMHIQQFRDVYGNKKKELQFMRDNLLRIVLYYDDLSYELLEQK
PSYDLLSWLGDIGGQIGLFVGSSAMSYFEFLDCLIMIIYAKYFKQYK
```

```
>HyNaC4 CAL36113.1 hydra Na channel 4 [Hydra vulgaris]
MNFEELAKIVVEAVQENNNQPINTNKIKTPRELRNNKTKEYVSEMIDNSSFHGISYIAGKENHFIRRTIW
LLITMTAFGYAAQKVYESTVNYFSFPISTTQMRIYVNEIDFPAVSFCNFNEFRLSKMDGTKVDQAILNPK
LQGLVTAEEYRNVTFGAMFDLKEMLVDCEFNGIPCSDENFTMFSWMQGERCFTFNSGKFPHKLLKIGGAG
MKRSLKITINIIHYEYYKDEMDAAIHMIVHGQQDTPLKMRGPTLSPGFTTYIQLEKKMIINLEAPYKTKC
GSLKLKYFDSYSLDTCWLEQLTDHVHSVCKCKDFFMPGDIPICSLNDAMSCMWPEWARFDQLKMQNCPLP
CVIDSYKLSLSRALFPSPQYADSLTNSLQQPPHIINSIKNISDKVQFARDNLLRFVIYYDDLSYELIEQK
PSYDTLVWLGDIGGQIGLLIGAGAMSYFEFIDCLFLVIYNQFFKTYR

>HyNaC5 CAX62741.1 hydra Na channel subunit 5 [Hydra vulgaris]
MPQKKKLKEIAEERLNAKEIFSRYIEASTLHGYAYVCSDTFYIRRVLWAVLMLLGGIYFVFKLKVGIIEY
FQYPFSTLSTEDFPEVLTFPAISICPINNFNISRVQNSILKQLYDADRLPLDKNWIDPGFDIPGNELADI
LKRSSLQIDKMHFYCDWISRNTTHPYIGPNPCGIENFTEYFNFKDQLCFTLNSGKKGFNLLSVDHEGLTY
GYELGLDVHTNEIISSYPYNGIKLIIHNQQESPVMSDGFVISPGDRTFIKMATVEKKSLQPPYSTECGSV
ELKYHSVYNRRACLLELLTEYLGLKCGCRSLYMPDNGMQYCSLKETFTCMFPAQESFNEYEMGKRCPVEC
NSITFKHELTYSRYQPQSVSVPLMETLSTKNITRPVLKLMKEKNMGYNEIINYIRENIIFVIFFFGDMKK
EVHEQEATFDFYQFLGDMGGEIGLMLGASLLTFVEFMDLLIFFVYHQLLRIQS

>HyNaC10 NP_001296656.1 acid-sensing ion channel 1-like [Hydra vulgaris]
MAKVEDKIKSPQEQKRRTIKQYLAYAGENISIHGLSHVFDKRENFICRTVWLLITIAAFGYAVQKVYEST
LNYFSYPFSTARMKIHVNQMDFPAVSFCNLNDFRYSATKGTKLDKAILSNDKKSISGEEYLNITLKARHK
IEEMLVDCEFDGNKCSVHNFTEFNWNQGELCFTFNSGKFPHSLLKVNGVGMKRSLVLTINVQHYEYYGDE
LDAGIHLILHDQEETPIKKRGPVIPPGFTTYIQVEKKTILNLESPYKTKCGSIKLKYFDSYSMHTCWLEQ
LTDHVYKICKCKDFFMPGEIKVCSFEVLENCTWKEWAKFDNYKLHTCPLPCKIDSYEMSLSRALFPTARY
ASSLANRFRKLPHVLNTVKSISNDLLFMRENLLRVVIYYNDLSYELLEQKPNYDTLVWLGDVGGQIGLFV
GAGVMSYFEIIDCLVLIIYTRFFQKFKTINSIRNHSL

>HyNaC3 CAL36112.1 hydra Na channel 3 [Hydra vulgaris]
MLNFQDIAQITVEAIQEKNELPNEEEKNTQHPRDLRNKKIKEHISYMIDNSSFHGLSYIFDKRHSIRRTI
WFFITIAAFVYAMQKVYESTMNYFSYPFYTARMRMYVNQINFPAVSFCNLNDIRFSVMNGTIVDDAIINN
NQEANITGEEMRTFIQAARHTLKEMLVDCDFEGKKCSYENFTEFSWMQGESCFTFNSGKPPHTLLKVNGA
GINRSLKLTINVQHYEYYRDKMDAGIHLILHGQDETPVKMRGLHLPPGFTSYIQIEKKTIINLEAPYKTK
CGSVELKYFDSYSMHTCWLEQLTDHVYKVCKCKDVFMPGDIPICSFDEAFNCMWPQWETFDKLKLYHCPL
PCKIDSYAVSLSRALFPTARYASSLANELRKHQHVAMNLKSNTDELAFMRENLLRLVIYYDDLSYELLEQ
KPNYDTLVWLGDVGGQIGLFIGAGVMSYFEFIDCLAMILYTRFFEKFTTK

>hENaCalpha NP_001029.1 amiloride-sensitive sodium channel subunit alpha
isoform 1 [Homo sapiens]
MEGNKLEEQDSSPPQSTPGLMKGNKREEQGLGPEPAAPQQPTAEEEALIEFHRSYRELFEFFCNNTTIHG
AIRLVCSQHNRMKTAFWAVLWLCTFGMMYWQFGLLFGEYFSYPVSLNINLNSDKLVFPAVTICTLNPYRY
PEIKEELEELDRITEQTLFDLYKYSSFTTLVAGSRSRRDLRGTLPHPLQRLRVPPPPHGARRARSVASSL
RDNNPQVDWKDWKIGFQLCNQNKSDCFYQTYSSGVDAVREWYRFHYINILSRLPETLPSLEEDTLGNFIF
ACRFNQVSCNQANYSHFHHPMYGNCYTFNDKNNSNLWMSSMPGINNGLSLMLRAEQNDFIPLLSTVTGAR
VMVHGQDEPAFMDDGGFNLRPGVETSISMRKETLDRLGGDYGDCTKNGSDVPVENLYPSKYTQQVCIHSC
FQESMIKECGCAYIFYPRPQNVEYCDYRKHSSWGYCYYKLQVDFSSDHLGCFTKCRKPCSVTSYQLSAGY
SRWPSVTSQEWVFQMLSRQNNYTVNNKRNGVAKVNIFFKELNYKTNSESPSVTMVTLLSNLGSQWSLWFG
SSVLSVVEMAELVFDLLVIMFLMLLRRFRSRYWSPGRGGRGAQEVASTLASSPPSHFCPHPMSLSLSQPG
PAPSPALTAPPPAYATLGPRPSPGGSAGASSSTCPLGGP

>hENaCbeta NP_000327.2 amiloride-sensitive sodium channel subunit beta [Homo
sapiens]
MHVKKYLLKGLHRLQKGPGYTYKELLVWYCDNTNTHGPKRIICEGPKKKAMWFLLTLLFAALVCWQWGIF
IRTYLSWEVSVSLSVGFKTMDFPAVTICNASPFKYSKIKHLLKDLDELMEAVLERILAPELSHANATRNL
NFSIWNHTPLVLIDERNPHHPMVLDLFGDNHNGLTSSSASEKICNAHGCKMAMRLCSLNRTQCTFRNFTS
ATQALTEWYILQATNIFAQVPQQELVEMSYPGEQMILACLFGAEPCNYRNFTSIFYPHYGNCYIFNWGMT
EKALPSANPGTEFGLKLILDIGQEDYVPFLASTAGVRLMLHEQRSYPFIRDEGIYAMSGTETSIGVLVDK
```

LQRMGEPYSPCTVNGSEVPVQNFYSDYNTTYSIQACLRSCFQDHMIRNCNCGHYLYPLPRGEKYCNNRDF
PDWAHCYSDLQMSVAQRETCIGMCKESCNDTQYKMTISMADWPSEASEDWIFHVLSQERDQSTNITLSRK
GIVKLNIYFQEFNYRTIEESAANNIVWLLSNLGGQFGFWMGGSVLCLIEFGEIIIDFVWITIIKLVALAK
SLRQRRAQASYAGPPPTVAELVEAHTNFGFQPDTAPRSPNTGPYPSEQALPIPGTPPPNYDSLRLQPLDV
IESDSEGDAI

>hENaCgamma NP_001030.2 amiloride-sensitive sodium channel subunit gamma
[Homo sapiens]
MAPGEKIKAKIKKNLPVTGPQAPTIKELMRWYCLNTNTHGCRRIVVSRGRLRRLLWIGFTLTAVALILWQ
CALLVFSFYTVSVSIKVHFRKLDFPAVTICNINPYKYSTVRHLLADLEQETREALKSLYGFPESRKRREA
ESWNSVSEGKQPRFSHRIPLLIFDQDEKGKARDFFTGRKRKVGGSIIHKASNVMHIESKQVVGFQLCSND
TSDCATYTFSSGINAIQEWYKLHYMNIMAQVPLEKKINMSYSAEELLVTCFFDGVSCDARNFTLFHHPMH
GNCYTFNNRENETILSTSMGGSEYGLQVILYINEEEYNPFLVSSTGAKVIIHRQDEYPFVEDVGTEIETA
MVTSIGMHLTESFKLSEPYSQCTEDGSDVPIRNIYNAAYSLQICLHSCFQTKMVEKCGCAQYSQPLPPAA
NYCNYQQHPNWMYCYYQLHRAFVQEELGCQSVCKEACSFKEWTLTTSLAQWPSVVSEKWLLPVLTWDQGR
QVNKKLNKTDLAKLLIFYKDLNQRSIMESPANSIEMLLSNFGGQLGLWMSCSVVCVIEIIEVFFIDFFSI
IARRQWQKAKEWWAWKQAPPCPEAPRSPQGQDNPALDIDDDLPTFNSALHLPPALGTQVPGTPPPKYNTL
RLERAFSNQLTDTQMLDEL

>hENaCdelta AAI25075.1 Sodium channel, nonvoltage-gated 1, delta [Homo
sapiens]
MAEHRSMDGRMEAATRGGSHLQAAAQTPPRPGPPSAPPPPPKEGHQEGLVELPASFRELLTFFCTNATIH
GAIRLVCSRGNRLKTTSWGLLSLGALVALCWQLGLLFERHWHRPVLMAVSVHSERKLLPLVTLCDGNPRR
PSPVLRHLELLDEFARENIDSLYNVNLSKGRAALSATVPRHEPPFHLDREIRLQRLSHSGSRVRVGFRLC
NSTGGDCFYRGYTSGVAAVQDWYHFHYVDILALLPAAWEDSHGSQDGHFVLSCSYDGLDCQARQFRTFHH
PTYGSCYTVDGVWTAQRPGITHGVGLVLRVEQQPHLPLLSTLAGIRVMVHGRNHTPFLGHHSFSVRPGTE
ATISIREDEVHRLGSPYGHCTAGGEGVEVELLHNTSYTRQACLVSCFQQLMVETCSCGYYLHPLPAGAEY
CSSARHPAWGHCFYRLYQDLETHRLPCTSRCPRPCRESAFKLSTGTSRWPSAKSAGWTLATLGEQGLPHQ
SHRQRSSLAKINIVYQELNYRSVEEAPVYSVPQLLSAMGSLCSLWFGASVLSLLELLELLLDASALTLVL
GGRRLRRAWFSWPRASPASGASSIKPEASQMPPPAGGTSDDPEPSGPHLPRVMLPGVLAGVSAEESWAGP
QPLETLDT

>rENaCalpha NP_113736.1 amiloride-sensitive sodium channel subunit alpha
[Rattus norvegicus]
MMLDHTRAPELNIDLDLHASNSPKGSMKGNQFKEQDPCPPQPMQGLGKGDKREEQGLGPEPSAPRQPTEE
EEALIEFHRSYRELFQFFCNNTTIHGAIRLVCSKHNRMKTAFWAVLWLCTFGMMYWQFALLFEEYLSYPV
SLNINLNSDKLVFPAVTVCTLNPYRYTEIKEELEELDRITEQTLFDLYKYNSSYTRQAGARRRSSRDLLG
AFPHPLQRLRTPPPPYSGRTARSGSSSVRDNNPQVDRKDWKIGFQLCNQNKSDCFYQTYSSGVDAVREWY
RFHYINILSRLSDTSPALEEEALGNFIFTCRFNQAPCNQANYSKFHHPMYGNCYTFNDKNNSNLWMSSMP
GVNNGLSLTLRTEQNDFIPLLSTVTGARVMVHGQDEPAFMDDGGFNLRPGVETSISMRKEALDSLGGNYG
DCTENGSDVPVKNLYPSKYTQQVCIHSCFQENMIKKCGCAYIFYPKPKGVEFCDYRKQSSWGYCYYKLQG
AFSLDSLGCFSKCRKPCSVINYKLSAGYSRWPSVKSQDWIFEMLSLQNNYTINNKRNGVAKLNIFFKELN
YKTNSESPSVTMVSLLSNLGSQWSLWFGSSVLSVVEMAELIFDLLVITLLMLLRRFRSRYWSPGRGARGA
REVASTPASSFPSRFCPHPTSPPPSLPQQGMTPPLALTAPPPAYATLGPSAPPLDSAAPDCSACALAAL

>rENaCbeta XP_008757904.1 PREDICTED: amiloride-sensitive sodium channel
subunit beta isoform X1 [Rattus norvegicus]
MPVKKYLLKCLHRLQKGPGYTYKELLVWYCNNTNTHGPKRIICEGPKKKAMWFLLTLLFACLVCWQWGVF
IQTYLSWEVSVSLSMGFKTMNFPAVTVCNSSPFQYSKVKHLLKDLDKLMEAVLDKILAPESSHTNTTSTL
NFTIWNHTPLVLIDERNPDHPVVLNLFGDSHNSSNPAPGSTCNAQGCKVAMRLCSANGTVCTFRNFTSAT
QAVTEWYILQATNIFSQVLPQDLVGMGYAPDRIILACLFGTEPCSHRNFTPIFYPDYGNCYIFNWGMTEK
ALPSANPGTEFGLKLILDIGQEDYVPFLASTAGARLMLHEQRTYPFIREEGIYAMAGTETSIGVLVDKLQ
RKGEPYSPCTMNGSDVAIQNLYSDYNTTYSIQACLHSCFQDHMIHNCSCGHYLYPLPAGEKYCNNRDFPD
WAYCYLSLQMSVVQRETCLSMCKESCNDTQYKMTISMADWPSEASEDWILHVLSQERDQSSNITLSRKGI
VKLNIYFQEFNYRTIEESPANNIVWLLSNLGGQFGFWMGGSVLCLIEFGEIIIDFIWITVIKLVASCKGL
RRRRPQAPYTGPPPTVAELVEAHTNFGFQPDTTSCRPNAEVYPDQQTLPIPGTPPPNYDSLRLQPLDTME
SDSEVEAI

>rENaCgamma NP_058742.2 amiloride-sensitive sodium channel subunit gamma [Rattus norvegicus]
MAPGEKIKAKIKKNLPVRGPQAPTIKDLMHWYCMNTNTHGCRRIVVSRGRLRRLLWIAFTLTAVALIIWQ
CALLVFSFYTVSVSIKVHFQKLDFPAVTICNINPYKYSAVSDLLTDLDSETKQALLSLYGVKESRKRREA
GSMPSTLEGTPPRFFKLIPLLVFNENEKGKARDFFTGRKRKISGKIIHKASNVMHVHESKKLVGFQLCSN
DTSDCATYTFSSGINAIQEWYKLHYMNIMAQVPLEKKINMSYSAKELLVTCFFDGMSCDARNFTLFHHPM
YGNCYTFNNKENATILSTSMGGSEYGLQVILYINEDEYNPFLVSSTGAKVLIHQQNEYPFIEDVGMEIET
AMSTSIGMHLTESFKLSEPYSQCTEDGSDVPVTNIYNAAYSLQICLYSCFQTKMVEKCGCAQYSQPLPPA
ANYCNYQQHPNWMYCYYQLYQAFVREELGCQSVCKQSCSFKEWTLTTSLAQWPSEASEKWLLNVLTWDQS
QQINKKLNKTDLAKLLIFYKDLNQRSIMESPANSIEMLLSNFGGQLGLWMSCSVVCVIEIIEVFFIDFFS
IIARRQWHKAKDWWARRQTPPSTETPSSRQGQDNPALDTDDDLPTFTSAMRLPPAPGSTVPGTPPPRYNT
LRLDRAFSSQLTDTQLTNEL

>mENaCalpha NP_035454.2 amiloride-sensitive sodium channel subunit alpha [Mus musculus]
MMLDHTRAPELNLDLDLDVSNSPKGSMKGNNFKEQDLCPPLPMQGLGKGDKREEQALGPEPSEPRQPTEE
EEALIEFHRSYRELFQFFCNNTTIHGAIRLVCSKHNRMKTAFWAVLWLCTFGMMYWQFALLFEEYFSYPV
SLNINLNSDKLVFPAVTVCTLNPYRYTEIKEDLEELDRITEQTLFDLYKYNSSYTRQAGGRRRSTRDLRG
ALPHPLQRLRTPPPPNPARSARSASSSVRDNNPQVDRKDWKIGFQLCNQNKSDCFYQTYSSGVDAVREWY
RFHYINILSRLPDTSPALEEEALGSFIFTCRFNQAPCNQANYSQFHHPMYGNCYTFNNKNNSNLWMSSMP
GVNNGLSLTLRTEQNDFIPLLSTVTGARVMVHGQDEPAFMDDGGFNVRPGVETSISMRKEALDSLGGNYG
DCTENGSDVPVKNLYPSKYTQQVCIHSCFQENMIKKCGCAYIFYPKPKGVEFCDYLKQSSWGYCYYKLQA
AFSLDSLGCFSKCRKPCSVTNYKLSAGYSRWPSVKSQDWIFEMLSLQNNYTINNKRNGVAKLNIFFKELN
YKTNSESPSVTMVSLLSNLGSQWSLWFGSSVLSVVEMAELIFDLLVITLIMLLHRFRSRYWSPGRGARGA
REVASTPASSFPSRFCPHPTSPPPSLPQQGTTPPLALTAPPPAYATLGPSASPLDSAVPGSSACAPAMAL

>mENaCbeta NP_001258952.1 amiloride-sensitive sodium channel subunit beta [Mus musculus]
MPVKKYLLKCLHRLQKGPGYTYKELLVWYCNNTNTHGPKRIICEGPKKKAMWFLLTLLFACLVCWQWGVF
IQTYLSWEVSVSLSMGFKTMNFPAVTVCNSSPFQYSKVKHLLKDLDELMEAVLEKILAPEASHSNTTRTL
NFTIWNHTPLVLIDERNPDHPVVLNLFGDSHNSSNPAPGSTCNAQGCKVAMRLCSANGTVCTLRNFTSAT
QAVTEWYILQATNIFSQVLPQDLVGMGYAPDRIILACLFGTEPCSHRNFTPIFYPDYGNCYIFNWGMTEE
TLPSANPGTEFGLKLILDIGQEDYVPFLASTAGARLMLHEQRTYPFIREEGIYAMAGTETSIGVLVDKLQ
RKGEPYSPCTMNGSDVAIKNLYSVYNTTYSIQACLHSCFQDHMIRNCSCGHYLYPLPEGEKYCNNRDFPD
WAYCYLNLQMSVTQRETCLSMCKESCNDTQYKMTISMADWPSEASEDWILHVLSQERDQSSNITLSRKGI
VKLNIYFQEFNYRTIEESPANNIVWLLSNLGGQFGFWMGGSVLCLIEFGEIIIDFIWITIIKLVASCKGL
RRRRPQAPYTGPPPTVAELVEAHTNFGFQPDTTSCRPHGEVYPDQQTLPIPGTPPPNYDSLRLQPLDTME
SDSEVEAI

>mENaCgamma NP_035456.1 amiloride-sensitive sodium channel subunit gamma [Mus musculus]
MAPGEKIKAKIKKNLPVRGPQAPTIKDLMHWYCLNTNTHGCRRIVVSRGRLRRLLWIAFTLTAVALIIWQ
CALLVFSFYTVSVSIKVHFQKLDFPAVTICNINPYKYSAVSDLLTDLDSETKQALLSLYGVKDVLDSTPR
KRREAGSMRSTWEGTPPRFLNLIPLLVFNENEKGKARDFFTGRKRKISGKIIHKASNVMHVHESKKLVGF
QLCSNDTSDCATYTFSSGINAIQEWYKLHYMNIMAQVPLEKKINMSYSAEELLVTCFFDGMSCDARNFTL
FHHPMYGNCYTFNNRENATILSTSMGGSEYGLQVILYINEDEYNPFLVSSTGAKVLVHQQNEYPFIEDVG
TEIETAMSTSIGMHLTESFKLSEPYSQCTEDGSDVPVTNIYNAAYSLQICLYSCFQTKMVEKCGCAQYSQ
PLPPAANYCNYQQHPNWMYCYYQLYQAFVREELGCQSVCKQSCSFKEWTLTTSLAQWPSEASEKWLLNVL
TWDQSQQINKKLNKTDLAKLLIFYKDLNQRSIMESPANSIEMLLSNFGGQLGLWMSCSVVCVIEIIEVFF
IDFFSIIARRQWQKAKDWWARRRTPPSTETPSSQQGQDNPALDTDDDLPTFTSAMRLPPAPEAPVPGTPP
PRYNTLRLDSAFSSQLTDTQLTNEF

>gENaCalpha NP_990476.2 amiloride-sensitive sodium channel subunit alpha [Gallus gallus]
MGTASRGGSVKAEKMPEGEKTRQCKQETEQQQKEDEREGLIEFYGSYQDVFQFFCSNTTIHGAIRLVCSK
KNKMKTAFWSVLFILTFGLMYWQFGILYREYFSYPVNLNLNLNLNSDRLTFPAVTLCTLNPYRYSAIRKKLD

ELDQITHQTLLDLYDYNMSLARSDGSAQFSHRRTSRSLLHHVQRHPLRRQKRDNLVSLPENSPSVDKNDW
KIGFVLCSENNEDCFHQTYSSGVDAVREWYSFHYINILAQMPDAKDLDESDFENFIYACRFNEATCDKAN
YTHFHHPLYGNCYTFNDNSSSLWTSSLPGINNGLSLVVRTEQNDFIPLLSTVTGARVMVHDQNEPAFMDD
GGFNVRPGIETSISMRKEMTERLGGSYSDCTEDGSDVPVQNLYSSRYTEQVCIRSCFQLNMVKRCSCAYY
FYPLPDGAEYCDYTKHVAWGYCYYKLLAEFKADVLGCFHKCRKPCKMTEYQLSAGYSRWPSAVSEDWVFY
MLSQQNKYNITSKRNGVAKVNIFFEEWNYKTNGESPAFTVVTLLSQLGNQWSLWFGSSVLSVMELAELIL
DFTVITFILAFRWFRSKQWHSSPAPPPNSHDNTAFQDEASGLDAPHRFTVEAVVTTLPSYNSLEPCGPSK
DGETGLE

>gENaCbeta XP_015149983.1 amiloride-sensitive sodium channel subunit beta isoform X2 [Gallus gallus]
MNLKRYFVRALHRLQKGPGYTYKELLVWYCDNTNTHGPKRIIKEGPKKKVMWFFLTLLFASLVFWQWGIL
INTYLSYNVTSSLSIGFKTMKFPAVTVCNANPFKYSEVRPLLKELDKLIEAALERILQPTHGDPISPLLL
NNSNATEGLDLDLWNQIPLVLIDEQDKDNPVIVEIFETNQSAAGNQTAAPPAPANVTSEEKKYKLAVKLC
SHQGSNNCTYRNFTSAAQAVTEWYILQSTSILSKVPLQERIRMGYQAEDMILACLYGAEPCNYKNFTQIY
HPDHGNCYIFNWGMDKEALNSSNPGAEFGLKLILDISQQDYIPYLSSAAGARLMLHQQKSFPFLKDQGIY
AMAGTETSIGVLVDELERMGYPYSDCTANGSDVPVKNLYSEYNTSYSIQACLRSCFQNHMTEICGCGHYM
FPLPEGVTYCNNEDNPGWAYCYSSLRSSIRHRQICIDSCKETCNDTQYKMTISMADWPSEASEDWIFHIL
SYERDMSTNVTLDRNGIIKLNIYFQEYNYRTISESAATTIVWLLSSLGGQFGFWMGGSVLCLIEFGEIII
DSLWITVINIISWCKGLKQKRVRARYPDTPPTVSELVEAHTNLGFQHEEAGTETQGEALPPEPGTPPPNY
DSLRVQPSHNPGTDSDIECEEQRPAANHHGDASVWAE

>gENaCgamma XP_015149986.1 amiloride-sensitive sodium channel subunit gamma [Gallus gallus]
MAPGKITARIKKTLPVRGPQAPTLRELMRWYCLNTNTHGCRRIVVSRGRLRRFIWILLTLSAVGLILWQC
AELLLNYYSASVSVTVQFQKLPFPAVTICNINPYKYSSMKDYLSELDKETKKALETFYGFSEGKTKVRRA
AGDWNGTESLFFRHVPLLRFENSFRAATDLRSGRKRKVEGSVFHKDSSIVNSGDSNDIIGFQLCDANNSS
ECALYTFSSGVNAIQEWYKLHYMNIMAQIPLETKEELSYSADDLLLTCFFDGLSCDKRHFTRFHHPLHGN
CYTFNSGENGTVLSTSTGGSEYGLQVVLYIDEADYNPFLVTSTGAKIIVHDQDEYPFIEDIGTEIETAAA
TSIGMHFTRSRKLSKPYSDCTETGADIPVENLYNKSYSLQICLHSCFQKAMVESCGCAQYAQPLPNGAEY
CNYKKNPNWMYCYYRLHEKFVKEQLGCQQICKDACSFKEWALTTSIAQWPSTVSEDWMLRVLSWDKGQKI
NKKLNKTDLANLMVFYKDLNERFISENPANTLVILLSNFGGQLGLWMSCSVVCVIEIIEVFFIDSFSIVM
RRQWQKAKKWWNHRKRDETGKPPEVGDAEQQGHDNPACSDEDLPTFNTALRLPLPQEGHPPRTPPPNYST
LRLETAFTEQLPDTLEAGQH

>gENaCdelta XP_004947475.1 amiloride-sensitive sodium channel subunit delta [Gallus gallus]
MEQEAAREEEERKEGLIEFYDSFKDMFEFFCKNTTIHGTIRLVCSSSNKMKTAFWTLLLLASFGMLYWQF
ALMFSQYWDYPVVLTMSMHSEPKMFPAITICNLDPYRFDLVSEHLAQLDRMAEKSVTVLYGINTSASLFH
VNEKSIHVRDLPSTGNHNGSSFKLSQKFSLLRTTEFNNRTGKRQSLVGFRLCNATGGNCFYKTYSSGMDA
ILEWYRFHYMNIMSQQPVIINISDHEEKIEDMVYSCQYDGEPCRPSDYVHFHHPVFGSCYTFNSKGTDPF
WTATKPGIPYGLSLILRAEQKDHIPLLSTVAGVKVMIHNHNQTPFLEHEGFDIRPGIATTIGIQQDKVNR
LGGNYGKCTTDGSDVKVKLLYNSYTLQACLHSCFQHIMVQKCGCGYYYYPLPPGAEYCNYNKQPAWGHCF
YQLYSRLRNHHLNCFDQCPKPCRESLYKVSAGTAKWPSRKSQDWIRQALRHQNGYNSTSNRKDIAKVTIY
YKQLNYQSVNESPLLSDNLLLSSMGSQWSLWFGSSVLSVVEMLELLIDTLVLSLLFCYQRFRSKTLNVAR
TPSIPSVSLTLESYRVVQEAGNGTAPAHGHTSGVPMAVANSSDPHPAQLSSKAIPEHCPDVVLNGFRYMK
DSSLGGEINH

>HtFaNaC AAF80601.1 FMRFamide-gated Na+ channel [Planorbella trivolvis]
MKYTGSEAKPSMSNSPHSYSSVKHRYQHTYSHSDQESDGHTVLGIIAELGSESNAHGLAKIVTSRDTKRK
VIWALLVIIGFTAATLQLSLLVRKYLQFQVVELSEIKDSMPVEYPSVTVCNIEPISITKILNLQNSTEGQ
KVINWLGFVTKFDFEQQKSFMDSFRAFYENLGDEAKMISHELADLLIHCRFNREICNLSNFTTSFDGNYF
NCFTFNGGQLADQLQMHATGPENGLSLIMSVEKAYPMPRFYGVYNFDNNILHSAGIRVVVHAPGSMPSPV
DHGIDIPPGYSSSVGLKALLHSRLPAPYGNCTMRSLQGMRTYRNTFFACLQLCKQRLIMSRCGCKSSALP
DLPTENVTFCGVIPNWEEIIKNVSGDVEPNMVIPTPALKCEERVQRELNNDRAYEMSCQCFQPCSETSYL
KSVSLSYWPLEFYQQLQLENILMTRNNTDKQHYMKKAYDILHTLSEEDRIRVMEKGVDVVPILMRRQKSL
AKDASDMVRQNLIRLNIYLEDLSVVEYRQLPAYGLADLFADIGGTLGLWMGISVLTIMELVELIIRLIGL

MFNVERKEPRAAVVPKKERAAPRRKSIGHYPTSNGDAKDVTYYPDTYGPSEFDFRRTAEAPV

>HaFaNaC sp|Q25011.1|FANA_HELAS RecName: Full=FMRFamide-activated amiloride-sensitive sodium channel; AltName: Full=FANACH
MKYTSAATKPGVFPEHHQHAMMRNRYHPHHCNYSDNRSAIDIIAELGSESNAHGLAKIVTSRDTKRKVIW
ALLVIAGFTAATLQLSLLVRKYLQFQVVELSEIKDSMPVQYPSVSICNIEPISLRTIRRMYFNNESQNLI
TWLRFIQKFRFEQDSFMNSIRAFYENLGQDAKKLSHNLEDMLMHCRFNRELCHVSNFSTFFDGNYFNCFT
FNSGQRLQMHATGPENGLSLIFSVEKDDPLPGTYGVYNFDNNILHSAGVRVVVHAPGSMPSPVDHGIDIP
PGYSSSVGLKAILHTRLPYPYGNCTNDMLNGIKQYKYTFFACLQLCKQRLIIQRCGCKSSALPEVPSYNA
TFCGVIKDWQEINRNHSNEDHNQSEEDRAFIPTPYLACEEREQKNLNNDRTYELSCGCFQPCSETSYLKS
VSLSYWPLEFYQLSAVERFFKQERQAGQNHFMKTAYEYLEKLAHPSQKHLARNDSHMDDILSKSYSLSEK
EMAKEASDLIRQNMLRLNIYLEDLSVVEYRQLPAYGLADLFADIGGTLGLWMGISVLTIMELIELVIRLT
GLVFNSEKGLPRGPTTVNNNNGSNNHSQSTSQHQLYNGYMDHDSHYSDSAGASVFDFRRGVESPV

>LsFaNaC AAK20896.1 FMRFamide-gated and pH-modulated sodium channel [Lymnaea stagnalis]
MYRSRVHSQNEAYARNGYISTRTRSETSSGHMKYTNIGSKPGMRPDQYSMGKPRGQPRHTALGIIAELGS
ESNAHGLAKIVASHDTKRKVIWALLVIIGFTAATLQLSLLVRKYLQFQVVELSEIKDSMPVEYPSVTICN
TDPISLRKIRRSVFSNESLLLRSWLTFIETFKFEQSAIMQSIRAFYENLGTEAKKISHDLQDLLIHCRFN
REECSVSNFTYSFDGNYFNCFTFNGGQLKEKLQMHATGPDNGLSLIISVEKDDPLPGTYGIYNFENNILH
SAGIRVVVHAPGSLPSPVDQGIDIPPGYSSSVGLKALLHSRLSEPYGNCTEGTLQGMHTYRNTFFACLQL
CKQNLIIRRCGCKSSALPDLPKENVTFCGVIPNWEKILRNESGDAFHGMTIPTPNLECEEKVHRELNNDR
AYESECQCYQPCSETSYLKSVSLSYWPVEFYQLCVLQKFFNATDPDHFMKVAFYNLDKVAREGQAEMASD
DNRKFNKSYSDEQKKMTKEASDLIRQTMLRLNIYLEDLSVVEYRQLPAYGLADLFADIGGTLGLWMGISV
LTIMELMELIIRLVGLVFSERDEPRRQTNETFTADNRRTHPKTSNAPQESNYPDPFGAPEFNFRRGVEAP
PI

>AcFaNaC XP_012938733.1 PREDICTED: FMRFamide-activated amiloride-sensitive sodium channel isoform X1 [Aplysia californica]
MWGRGKRQRNKNYPSGSGGGGGAFRSPAMRNDNELEGFVSILHTSGDNYVPIRDSSADHMKYTSVSAKSG
MVPEHRYTMVRSRHHGRHHHHHSYQEYNTQRSAISLIAELGSESNAHGLAKIVTSRDTKRKVIWALMVII
GFTAATLQLSLLVRKYLQFQVVELSEIKDSMPVEYPSVTICNIEPISLRKIRKAYNKNESQNLKDWLNFT
QTFHFKDMSFMNSIRAFYENLGSDAKKISHDLRDLLIHCRFNREECTTENFTSSFDGNYFNCFTFNGGQL
RDQLQMHATGPENGLSLIISIEKDEPLPGTYGVYNFENNILHSAGVRVVVHAPGSMPSPVDHGFDIPPGY
SSSVGLKALLHTRLSEPYGNCTEDSLEGIQTYRNTFFACLQLCKQRRLIRECKCKSSALPDLSVENITFC
GVIPDWKDIRRNVTGEYKMNQTIPTISLACEARVQKQLNNDRSYETECGCYQPCSETSYLKSVSLSYWPL
EFYQLSALERFFSQKNPTDQQHFMKIAQDFLSRLAHPQQQALARNNSHDKDILTTSYSLSEKEMAKEASD
LIRQNLLRLNIYLEDLSVVEYRQLPAYGLADLFADIGGTLGLWMGISVLTIMELMELIIRLFALIFNAER
EVPKAPVHSSNNGGGGGGDGQHNFANGDVEHERDTHFPDLGSSDFDFRRGGGIGAESPV

>ACD-1 NP_491295.2 Uncharacterized protein CELE_C24G7.2 [Caenorhabditis elegans]
MEPTLSPNYRNEAFEHDDSYLVNFVAGSSSGESSTPPPSFVPKCNFRYNQSRSQMIIEVPVAQLKKLRKL
EGTVSIKRETQHFCETTTMHGPKRIFQGKRWATLFWLIMVSCSLGLLITQVFILASEYLSKPTVSDVSFL
INEDGMDFPLITICNLNPIRKTYVNEINKTGEVSPPMINYMMKWFTEIPTLIGGADRPTLHEGNEELKLY
MKNHLNFTVDSFFMNSGFSCPDIFKLCSFQGEIFDCCTLSTEVLTPLGKCFTLDLSSSTKASMHKQTEPG
IQAGLAITLDAHLEEQFDGSNGMDALFTNSFVNGFRYFVHPPNTIPHLSSDEFTVSPNTVAFSAISSDRY
VLLPTHQWGNCTENFPDGIQSNLSYSSGNCLSLCKAKFYMENCGCTPALYNIENNLKECTPYETTTCLDN
ILAKPNKETGKIEFQTPNCKACAQQCNSLVYRAYNSYGSQFSAGAFHYLKSINPEWTDGHMRANFQMINI
FYRDMSYTEYNQVQDASVTQLLSDIGGNMGMFLGMSVITITEICLFFSKMFWLGFSKKRRDYMYSKRVNE
KTHEREVCETVEKMKAIASQGNLSSIAGTTSAKNSIPNDNVEFRINLKDLADQLDSDSGYSQNQPDSRNA
SFQKY

>ACD-2 NP_001309477.1 Uncharacterized protein CELE_C24G7.4 [Caenorhabditis elegans]
MHLEDGPSTKPPDFENEKTQETSLSGEEFENNSTLGTMRDAKSWAAANKQFANQLVIQVPVNSFKNGKKI
KGVGSAFRETKHFSSTTTMHGPKRIFYGKGVARAFWMLIVGLALAMLCFQIFILLQMYFSKPTLSQVSFI

VNEGGMDFPAVTVCNFNPIKKSYVRELNVSGDLTGETLEYLLQTNMDAMFLFSNLDRHNLKETHDEAETY
FQNHTDFQIIKFLRTAGYDCGEMFMTCYFGGRRFDCCKYMKQKVTSLGKCWELDLRNLAPEWMRKQISPG
SEAGLQIVVDAQLEEELKGENDDAKAIFSDIYENGFRYFIHPPGTNAQLTSEGISVSPSRTVYSAIKTVT
HNLLNRGNWGNCSENWPEGYNTFLSYSASACRALCIAQFFNDTCGCAPFTYNVDGRKKICAPYESITCMD
NHMLKKVNGTDYLELPDCEECHMECQSTSYTSYNSYGDGFNRGSLEWLKKISNKSETHIKNNVAVINIFF
LEMFYTSYSQVQATSLTEILSDIGGNMGMFLGMSVITITELSLFFSKIFWIMVSKRRRQYMYSKKTHEKE
KEHQLDEAVKEFQERRSRRNSRENISALGHYSNRITPVDDFQTKFGYKNAFSEGNMSNSSLDSVMELKFD
INELRRQLNQPSTDGIARIRLPHQTSRQNSTENYYSSSPPIFTIEPMSRKQSKTSLPSSLSPR

>ACD-3 NP_001257250.1 Uncharacterized protein CELE_C27C12.5 [Caenorhabditis elegans]
MTETSNCSSSSSSEYEEEEERIVLHVYDDESKEFTSLTTYHGMIRIYTSETWPSRIFWGVVVVTCVTLFMI
QGGVLLEFYNSHPTATKIDEYRLPTSFLPSISICPYGFKTDDNLFYLITQGDKDVYIPPDYWKNSSKQLL
KRLSYKCEDVVESIMINPNQIIDFCANSRTQITEIGKCFTFENWREFETNTLKIKLKSDFTKMYTAHIHS
EYYEVSRSSTQAWLKPGSHAKLSFRIEEQHNLPQNNWGTCKVQTGEIYNHLGCLEQCLVAGYDQSCHCSP
FFNRFTRFHCSIDELLNCPKLKEVPCDCPMQCYSQNYVLQPVSLLKSRSNISTVTFHLNSNLLRSHQQYK
RFKQIDLMSYIGGVMGLFLGMSCVTLLEVFIYLFKTIFGTLNSTRHKAFIERLLSNEDGSIHGSHEEIII
TQKIEKTVQKETLEQPLEQVADTRPLAERRFSLMPNNQLGVKVQFHRPNHLLKRNSVYLGNCDF

>ACD-4 NP_505230.1 Uncharacterized protein CELE_F28A12.1 [Caenorhabditis elegans]
MNRKRKLSCFVSVKFPVDSVKKLRKTEGVGSVYQETQHFSTITTVNGPRRIFYGKRSAQIFWILVVISIL
AFLVYQIVILIQYFYSKPTLSQINFITNEGAVYFPSVTVCNLNPVKTSFIKKLNSSGDLSEELLNYLLAT
KTNSMYMFNNANIFELKRAHLNALVYLANHPDFEIVNFLNSAQFDCDELFETCFYGGKQFNCCKYMTQSI
TSLGRCWELNLRNETDAWLTKKGRSGTSPKTGLQIIANARQSEQFINFHYSSFQENGFRYFIHPPHVSPD
LAAEGITVSPSRVVNSAIKTVLHDLLNHENWGNCTSSWPEHYNTNLPYSSSACQALCVSNYFKKLCGCSP
YSYNIDNNTQVCLPYEEVICMMEKMTKSDSNGTVSLDFPFCAECHLACQKTSYSSYTSYGDGFNYNSMKW
LTRETNRSASYIRQNIAIINIHFMELFYTSYSQVKATTILNTFNKIFGLNGLWFGMSVVSLTELILYFTK
ISWIAVSSKRRQYLFEKKMSEKRKERNIEEAVQEAEISRSRRSSAANLKFLDIESLDDEDYWYRSSSQLS
DSHLDNVIQLAIDFEKPLQRPSAISLPRISECCEDFEEEDEDENNDLGIIIKL

>ACD-5 NP_491196.3 Uncharacterized protein CELE_T28F2.7 [Caenorhabditis elegans]
MRRVRNLSLLYNDGPMGRFADQENPVENRNKKETVHFQSGSYDDDMSNSPSSSCSTVGDMPNIKPSASKG
SFLSELKPFSKRASQLIVDVPVAHLRKIKNTEGVSSITRESEHFSNTTTLHGPKRIYNGKGWSCVFWVFI
WISSMIMLLTQVTSLISMYISKPTVSQVSFLLSEGGMQFPRVTVCSFNPIKRTTVEALNSTKDLSDDLLD
YLMMFNSDAMTLYGRADAASLHSGDNVFKHYVSSHPNFTADNFFMDAGFSCGDMFKMCSFGGRRFDCCKY
ATPIFSDLGKCFTLNLQGSDKSWMKMQTEPGIAAGLQIILDSHLEEQFDSETDGVTPVFSSAFENGFRFY
IHSSEEIPFLASEGIAVSPDSVVYSALSSSKYILLSSNAWGNCSDSWPRGYDYSFPYTSAMCSTMCKAQY
FQNLCGCSPSIYNHLNRFNDCTPYETFICMDTKMKKVVNQSFNIEMPTCEECKVECKSQVYHSFNSYGKG
LSRGALMWLTKQGKQETWTIPHMKLNFQVVNVFFRDMSYTEYIQKRGMSLTELLSDIGGNMGMFMGMSVF
TIIELFLFLSKIGWIGFSRKRRDYMYSKKKNEEMHEKELEDVVTGFKLFRHRKSGKDMSHLREKIKGLSM
HRVTSEQLNVCKLAWENEPDIERRLASVTRQNSALKEHKDYKQPTILPFDLKDIKDQITRGRAASMFRSR
RSRSETAPAVIHEA

>DEL-1 sp|Q19038.1|DEL1_CAEEL RecName: Full=Degenerin del-1
MARKYIDILKKSKMMLFQDVGKSFEDDSPCKEEAPKTQIQHSVRDFCEQTTFHGVNMIFTTSLYWVRFLW
VVVSLVCICLCMYSFSHVKDKYDRKEKIVNVELVFESAPFPAITVCNLNPFKNHLARSVPEISETLDAFH
QAVVYSNDATMDELSGRGRRSLNDGPSFKYLQYEPVYSDCSCVPGRQECIAQTSAPRTLENACICNYDRH
DGSAWPCYSAQTWEKSICPECNDIGFCNVPNTTGSGNIPCYCQLEMGYCVFQPESRVRRIWEFQGNKIPE
KGSPLRKEYMEQLTQLGYGNMTDQVAITTQAKEKMILKMSGLHPQRRAALGYGKSELIKMCSFNGQQCNI
DTEFKLHIDPSFGNCYTFNANPEKKLASSRAGPSYGLRLMMFVNSSDYLPTTEATGVRIAIHGKEECPFP
DTFGYSAPTGVISSFGISLRNINRLPQPYGNCLQKDNPQSRSIYKGYKYEPEGCFRSCYQYRIIAKCGCA
DPRYPKPWKRSAWCDSTNTTTLNCLTTEGAKLSTKENQKHCKCIQPCQQDQYTTTYSAAKWPSGSIQTSC
DNHSKDCNSYLREHAAMIEIYYEQMSYEILRESESYSWFNLMADMGGQAGLFLGASIMSVIEFLFFAVRT
LGIACKPRRWRQKTELLRAEELNDAEKGVSTNNN

>DEL-4 NP_492230.2 DEgenerin Like [Caenorhabditis elegans]
MGVFWTGLKYVFTDFSCWTSTHGVPHIGMANARWLRAFWILVVVVSIALFIWQFITLLTNYLSFSVNTET
TLQFAERTFPTVTICHLNPWKLSETKSVDPDMSALIDAYNSDSSSAQFGLPASLTADRQQQASKWTLMYS
ERLNEKQYNDAADIAYSYDDMVVSCTYNAKTCNITDFNDFYNPSYGNCLQFNTDGMYSSSRAGPLYGLRM
VMRTDQDTYLPWTEASGVIIDIHMQDEIPYPDVFGYFAPPGTASSLGVSYVQTTRLSKPYGSCTTKTKLK
TTHYTGTYTVEACFRSCMQEKIIASCGCYYPAYSHASNTTQYVSCDNGVQTLSNLNCVDLINSADSTEFD
VLTDCDCPQPCEIDSYGVTVSTAQWPSDSYVPTECNPGGPSGPWDASGESCLDWYKANTILIEIYYERMN
FQVLTESPAYTFVNFISDVGGQVGLFLGMSIISAIEYLVLIFLVFFYCCTHKSRRAEIEQLEMDIKKAKD
DVDQVAEKRKKHQKANAELYEMDTAHDIVPPKPHSND

>DEL-7 NP_501276.4 DEgenerin Like [Caenorhabditis elegans]
MNCSCGHQTDRERVTHAARNMYIMKPPPENEVFISPITLASVNTTQYEEALKIAQFHCNCKYLWLRELHG
LSAFMMSNSLMSKVFWAIVIMACAGWSIGNTISILKQYGDEATTTLLTILPTKQLKFPTMIFCPRNPDFL
NYYNVLEDMYNHLGYMENTTNFHILQYAMTGFGFDNANGDTFNETYREQIHIYYLKWRGERTQYEMFDFM
FNKNGYTCTDLFQTCYGGSQTYNCCDIFQPTYAMLRGRCFRLIDSYYQNDTDEVAKLSIFFNNMTSPILN
TGVLPQLVLYNGDSNVEIGIYPRYYLNSNDWNRVRFYQKSMILLPKSDGCSTDPIYQGKFTCFVYKWLMQ
LIEQYNCTVPYYKYTLSYLKDVPICEPDVIVNNFENISLTPSTIGYKCTSACSRIENTVTLATSIDTDPD
PSYMFRIEASFTYLEYEQYKEIRTTSTAGFISELGGQAGLFVGSSVMSFVQLFNSVFIQIYKILRNYCNK
KGIRVRIGLYQTHPSEPADKYYERDAALSDEPPYPLDTILEVEEPNTMDADGMLLEAGDPISEVDAIELE
TWSSVSEIESFDSSNYLPTPSFSSSEQHSTICEEPEDIRTAKLIWIRENREMDL

>DEL-9 NP_508622.2 DEgenerin Like [Caenorhabditis elegans]
MYMNGNFPETTVVRSHSLTQSQSLSDSAESAIRRLKKENHCLKKTLSRRWSGRSRASTRSHDPTDASSLS
DSSDDNDDSLYLLRETSTLHGLRDVMLSSSGRLRMVWVLIVILALFMTFQGCYQIMDEYSMRRIVVSYFI
QEAQSIWVPDVVVCPYNRLNRSFIEANNVSFELAQFLELSFPSMDLPFENIQQQQEEIISKIDTLDFQIE
ILLEQNNMTYAQFLRKASLNCEAFFEDSRRCANTTEIMTSAGKCFRMAGIKQEIAGFGNGDRYVIDLPEE
YYNPGINQMINSGVIIKLAERGQGIDNDLTFLPAGVHAIMPLLGTQFEFMNDPPRYECEEDPHGNYSRVH
CFEDCLTLDAQQTCQCSPAAAQNPAYPDKLCTATQLYHCFFTKLFPEDSNLSKAIVDACKKECKAPCHAW
NYNKQVSYSSIPSEASKKLLPREEWEKMKRKIILDIYYSELDYTIIKHVIAMPLSSLIAQIGGQFSLFAG
GSLISLCQIVIYSVRYVMHKFCGLKSQRRRCRETREAHEARQRRDMRRRRRNSTSHSRSTPKPHNGNGNG
KVVNVEMVTTHTSETTPPI

>DEL-10 NP_495302.3 Degenerin-like protein del-10 [Caenorhabditis elegans]
MVRMAERLAENFIPEANQRNENEPAYSRYKRVGQNRSRLNSRASLGSSMGRRITLIETDSGVIEVESDKQ
FLDAFKDANMDAVHHLNAASPVTRGLWCMIIIAFVILVLVQCYSQIKLYISEPVATNIEAEYPSKISFPT
VAICNNNQFRLTYLTGGRIMNRRSKSISGSLLSTGHDVESVFDTVLRKSWDMDAVKFLRSAAHWKSRMIL
GCTWPNGTSCKLSDFKAVWTTTGLCWAINTDPHNPYEVTGSGEGHGLRLLLNVESYERVDACTKHFRTKT
LPGLKILIYNQTDIPDSSMNGVNVPSGYSMDIPFKMQHRSKLTGVHCIEENDEQIEASTDFNNPENIRTC
TLRRYMTEVENSCHCTLRRAYTSNSTDVKMKACNVDQYFGCAQKAMQRIREEGTASTCLPPCKSIDYTAW
QDMNRLPQNLMPALIEEQEEDDEDDVEQEELDENVSFSTVSGGETFSCEDSAYLDDKQVMRIKRDAHRAY
EMQARHQEDIFLRSRRLIARLRNAINSIERYKWGWHYDTFSGVADRLSNLTCFSNFSERHRDIISILESR
PITSEEKKANQMFFLLDETAFNRNATRYMSVGDLKSRYGDKVDDVAEEIAVILRIMEKLWHVFMPDSYIR
TMTGDFSRMDRIIELMNQYELNKLQRRAWAEKMQSRQMKHFFEDDFYESYYQPLIKDLDTTLVKQIDEVE
ADWPKVEYYLQRGSAGKTGAIMFFGDGNKDNRQKFEKLIVEMHECASGKMRKEAGKMLSSFKKSYRELQA
AYGKLFKEELPDYLENFQFGNKFVGDNFAMVNIFLHRMNLEVWSQDRTYGFWSLACDIGGALGLFLGASL
LTIIEIVYLCIQYGLCGKRARNMKCIPMDALTRQMKKVATCSCCKKPIEKSREPIYKKKSQSYQRRFTAD
DEDQGDKFRSRASSEESKRRKNIWAQMNDPSGNSTLTPSEIKNFLDQVQRNSQPPSYHDDHHPEDHYYYN
DPNYLTISPPSDRPEDNREGSTSGYYSSPRAPPQANPRDESPIPDTEPISVSEFSDALAPPLFKTPPRER
RTRKEQKIDEEDDDKHSYV

>MEC-10 NP_509438.1 Degenerin mec-10 [Caenorhabditis elegans]
MNRNPRMSKFQPNPRSRSRFQDETDLRSLRSFKTDFSNYLASDTNFLNVAEIMTSYAYGESNNAHEKEIQ
CDLLTENGGIEIDPTRLSYRERIRWHLQQFCYKTSSHGIPMLGQAPNSLYRAAWVFLLLICAIQFINQAV
AVIQKYQKMDKITDIQLKFDTAPFPAITLCNLNPYKDSVIRSHDSISKILGVFKSVMKKAGDSSSEALEE
EEETEYDMNGITIQAKRKKRGAGEKGTFEPANSACECDEEDGSNECEERSTEKPSGDNDMCICAFDRQTN
DAWPCHRKEQWTNTTCQTCDEHYLCSKKAKKGTKRSELKKEPCICESKGLFCIKHEHAAMVLNLWEYFGD
SEDFSEISTEEREALGFGNMTDEVAIVTKAKENIIFAMSALSEEQRILMSQAKHNLIHKCSFNGKPCDID

QDFELVADPTFGNCFVFNHDREIFKSSVRAGPQYGLRVMLFVNASDYLPTSEAVGIRLTIHDKDDFPFPD
TFGYSAPTGYISSFGMRMKKMSRLPAPYGDCVEDGATSNYIYKGYAYSTEGCYRTCFQELIIDRCGCSDP
RFPSIGGVQPCQVFNKNHRECLEKHTHQIGEIHGSFKCRCQQPCNQTIYTTSYSEAIWPSQALNISLGQC
EKEAEECNEEYKENAAMLEVFYEALNFEVLSESEAYGIVKMMADFGGHLGLWSGVSVMTCCEFVCLAFEL
IYMAIAHHINQQRIRRRENAANEY

>MEC-4 NP_510712.2 Degenerin mec-4 [Caenorhabditis elegans]
MSWMQNLKNYQHLRDPSEYMSQVYGDPLAYLQETTKFVTEREYYEDFGYGECFNSTESEVQCELITGEFD
PKLLPYDKRLAWHFKEFCYKTSAHGIPMIGEAPNVYYRAVWVVLFLGCMIMLYLNAQSVLDKYNRNEKIV
DIQLKFDTAPFPAITLCNLNPYKASLATSVDLVKRTLSAFDGAMGKAGGNKDHEEEREVVTEPPTTPAPT
TKPARRRGKRDLSGAFFEPGFARCLCGSQGSSEQEDKDEEKEEELLETTTKKVFNINDADEEWDGMEEYD
NEHYENYDVEATTGMNMMEECQSERTKFDEPTGFDDRCICAFDRSTHDAWPCFLNGTWETTECDTCNEHA
FCTKDNKTAKGHRSPCICAPSRFCVAYNGKTPPIEIWTYLQGGTPTEDPNFLEAMGFQGMTDEVAIVTKA
KENIMFAMATLSMQDRERLSTTKRELVHKCSFNGKACDIEADFLTHIDPAFGSCFTFNHNRTVNLTSIRA
GPMYGLRMLVYVNASDYMPTTEATGVRLTIHDKEDFPFPDTFGYSAPTGYVSSFGLRLRKMSRLPAPYGD
CVPDGKTSDYIYSNYEYSVEGCYRSCFQQLVLKECRCGDPRFPVPENARHCDAADPIARKCLDARMNDLG
GLHGSFRCRCQQPCRQSIYSVTYSPAKWPSLSLQIQLGSCNGTAVECNKHYKENGAMVEVFYEQLNFEML
TESEAYGFVNLLADFGGQLGLWCGISFLTCCEFVFLFLETAYMSAEHNYSLYKKKKAEKAKKIASGSF

>FLR-1 NP_510243.1 Uncharacterized protein CELE_F02D10.5 [Caenorhabditis elegans]
METETESERIYLQLYDYETKEFSGLTTYHGLVRIYNSNTWPSRIFWVVVVLSCLSLFMIHSGYLLLGYHA
KPTLFQTNTIVPMNGLLFPEVTICNLNPLNTTKLEELNISKSTWTYIFGYFDEITTSEHKSTKLGEEQFL
EIMNNYQELTKQEFNVKNFLKSVSKSCEETFISCSFGREKLHNCCEHVTTEMTEVGVCFRLSNVNKKYRQ
WYSGNGFGWEFVLNGNNEIDDHADSLDFEPDRGFLIMVHESEKYPKINSYGVAVSPDSQLHAAISMKNIS
LLDKANWGSCSKGWNRNDTDVPYTATHCEIDCKLRKVRNLCGCSPLAYSARESGSNDTICTPYQIQQCFR
KVRGLDNRWEDECDCPSECNMLEFDVTNSYSDLDGRSRGLSSSKVESDISHVSLYFSHVAYERIEQQKQL
QTADLLSNIAGSMGLFLGMSTVTLLEIFIYLFKSVWGTVNSTRQQQFVDAVAEEEKERSESIVIIQNGRN
DDMDDQKPSRFPGADRKLSGNSIHIHLDRRNSRMIRGGDLSAASRGSVSIPSQLLSPLSRHNRQSISYGQ
LGRKVSAIGIPLQPNHDTVESGTSLMPPKSPIRRCSTSTTPSMLTRKLSFASQQSDPAQPAHQSRKVSTS
SIFKSQLI

>DEL-3 NP_492135.1 DEgenerin Like [Caenorhabditis elegans]
MWLRGLFGGLFFLFLLCSCVALFVLHSLYFIFRTASQTEKTESKTVHDDQLFIPALVICNRMPFSQDGLN
NVNVNLRQDSALRYLLEWTNPSLREAADYVAPSADLMNQGQNTVLQYITQSTRNQTIQNMQYQCQSVINS
CTYQGIQLSSFDCCRNLLSMIPSTNGLCWVWKDSTMWQNSTGINRQFSITFQMTRNSWFSTYVPTHPGVD
IYLREDGNDVMRMATELENPIRLLDKRGVRLQMRKTKKADIRRTSCGYALGDARRSDEHAFKNNHTNYLM
CNMLVAMRYCSCHPLMAELIHYDPSSYRDFLLRVTQTSVCSVDAYDNCARRYIDLTRIENWEEDIPKDLP
GYEDAKKCRNDNQRSCLITSYPGTIEGYDLPEEYRTTQDYVSRLVLEYSTMRTTEILVSKDPNIYELLSF
IGYNMALWFTVGHILWSMFWYATGLCCPKRQSSNRISPEIRRKRSVSSPEPIVVEHRPSQDVASGDT

>UNC-105 NP_001122595.1 Degenerin-like protein unc-105 [Caenorhabditis elegans]
MAEDRIKSKLRRPASIESTMSSRTKPRHKPSPMSILMPHLMVGESFRKYRPHGLRNIRMNGHLDWNQLRK
SFEKQSTFHGISHAATADGKWRWFWYTAFTICLLALLIQIFFLISKYRQYGKTVDLDLKFENAPFPSITI
CNLNPYKKSAIQSNPNTKAMMEAYSRRIGSGDKTEGIAAALSATGGLHAKVRRAKRKAKGKPRLRDRRYH
QAFAQCLCDIEQLTGDRKGSCFAAFKGKIEIDTNNTAGFMNLHTSRCLCQLDTVSKALWPCFPYSSWKEK
LCSECVDNTGHCPMRFYKGNELYENIKEQVDLCLCHKEYNHCVSTRDDGIILEISPNDELNDLDIGKKIA
SQLSAQQEKQAEVTTTEAPTVTQALGFEELTDDIAITSQAQENLMFAVGEMSEKAKESMSYELDELVLKC
SFNQKDCQMDRDFTLHYDNTFGNCYTFNYNRTAEVASHRAGANYGLRVLLYANVSEYLPTTEAVGFRITV
HDKHIVPFPDAFGYSAPTGFMSSFGVRMKQFIRLEPPYGHCRHGGEDAATFVYTGFQYSVEACHRSCAQK
VIVEACGCADPMYPVAEMFGNNTKPCQAVNMDQRECLRNTTLWLGELYSKGKEAIIPDCYCHQPCQETNY
EVTYSSARWPSGSAKVMECLPGDFLCLEKYRKNAAMVQIFYEELNYETMQESPAYTLTSVLADLGGLTGL
WIGASVVSLLEIVTLIVFATQAYVRKRKGSISAQSHHSVPVHRASRVSLNTLHKSSTTQSVKLSVMDIRS
IKSIHSNHSSKSKQSILIEDLPPAIQEQSDDEEETTESSRTNGSCRYLAPGEDLPCLCKYHPDGSIRIMK
ALCPVHGYMVRRNYDYSVSNSEEEDAEDEVHREPEPFYSAPYEHRKK

>UNC-8 NP_501138.1 Degenerin unc-8 [Caenorhabditis elegans]
MSPLLTWNLICVSSRWYTILCLKNKVKFWLGTRLVHEPESMESRSSPYIRPSPYAGGVHPHFEEEDDRSK
LHASALYSERRTSSRKSLRSQKIDYHTTTIKSLWFDFCARTSSHGIPYVATSSFFGRYVWAALFMCMLMA
FLLQTYWTMSEYLQYRTIIEMQLQFEAAAFPAATVCNLNAFKYSELTQYEEIKEGFDYWERVINARMMSD
SMKPGGDILEAISVRKKRSKSRDQLLFPIDDEDLEGAVYQPVFVRCTCMNMEQCVPNRNPLEVNASICMC
FEDVTRGLIWPCYPTSVWTVKKCSGCSISNTCPDPDGPNASKQIAKHNSPLPCLCQSISHHCMVHPKDEI
RWWNPNNYTVYSVTEPPTTEITETEEAFGLSDLKDAGAITTQTKENLIFLVAALPRETRRNLSYTLNEFV
LRCSFNSKDCSMERDFKLHVDPEYGNCYTFNFNDSVELKNSRAGPMYGLRLLLNVHQSDYMPTTEAAGVR
LVVHEQDQEPFPDTFGYSAPTGFISSFGLKTKELHRLSAPWGNCSDTFRPVPYIYNEHYSPEGCHRNCFQ
LKVLEICGCGDPRFPLPSEEHRHCNAKSKIDRQCLSNLTSDSGGYHHLHEQCECRQPCHEKVFETAYSAS
AWPSQNFKIGTDCPAVSDIFNDTEACTEYYRQNTAYIEIYYEQLNFESLKETAGYTLVNLFSDFGGNIGL
WIGFSVITFAEFAELFCEICKLMYFKGIVYVQKKMQGKEYTSSSLMHIDFLQRSPKKSQPGEDEVSTNES
TKELMSK

>PPK4 NP_001334723.1 nach, isoform D [Drosophila melanogaster]
MGHQEELKPEQVDLKVTPFVGYLRRTWSDFCATSSIHGLKYTRDEDTNKIVHLVWLLISVVMFICAVVMA
RTFYMDYRSSPTRMNVESDNTPVNRLYFPPVTICPDVLFNMQKSEAFLNTLRLPKGAELRGILRKLHIFY
GFMLDDERYSAEDIEQMEALLFLNNLTIPEFVEHLRWNCDEILYRCRFNGEIMDCSKIFQLSKTFFGHCC
SFNLRQKGWVNNKLNNLESFKVFHLNSLNFTAQRAIGGLRYGLSVVVRYKDDNYDPLQSYSYGVKLLIQE
ADAFPSAHSAAKFIAFNSETFAAVRPQETFCSSAVKALIIEERNCVFQNEFPMRYFSDYVYPNCELNCRV
TNMVKFCGCHTYFFDFNRTSDRICTFRDIPCLVDNFANIITRKKSTQCYCPLTCEHIDYDVQLTNFPLEL
NMPVADKFYSGLAKNDGVLHVFINSFSYRRLRRDLLSNMVTLVSNLGSAFSLFVGMSMLSVVEIIYYFSV
ILRKNYKLECETRSQMLHKKPKFAWPKANDTHSKEQKSVFIIHKS

>PPK5 NP_996138.2 pickpocket 5, isoform B [Drosophila melanogaster]
MEMLKYVISDNNYSWWIKLYFAIIFALVLFVAVNLAVGIYNKWDSTPVIIGISSKMTPIDQIPFPTITVC
NMNQAKKSKVEHLMPGSIRYAMLQKTCYKESNFSQYMDTQHRNETFSNFILDVSEKCADLIVSCIFHQQR
IPCTDIFRETFVDEGLCCIFNVLHPYYLYKFKSPYIRDFTSSDRFADIAVDWDPISGYPQRLPSSYYPRP
GVGVGTSMGLQIVLNGHVDDYFCSSTNGQGFKILLYNPIDQPRMKESGLPVMIGHQTSFRIIARNVEATP
SIRNIHRTKRQCIFSDEQELLFYRYYTRRNCEAECDSMFFLRLCSCIPYYLPLIYPNASVCDVFHFECLN
RAESQIFDLQSSQCKEFCLTSCHDLIFFPDAFSTPFSQKDVKAQTNYLTNFSSEYMRKNLAVVNFFHTDN
YFRSSVRTSYTGPTEYMASTGGIMSLMIGFSVIFLAEIIYIILFVSTLLLILIF

>PPK6 NP_611461.3 pickpocket 6 [Drosophila melanogaster]
MIESGKWPTPGKKNSKGDGSRKRGKDPSLIRTAILATFWEYTERTKVSGMWLMRRNRTYGLSRFIWSSVL
LQLLLLSIYLTLLLWLKFYSYPILNTISNDLSITDVAFPGVTICSPKVVNSERVDRYVKTLKIPKEYDMA
EVIAGFDFLNAFTDQSFEPPGHDSYRATDAVLRLNNVSIWEAAMAVSPGCFDYVKRCFWGHTEFQCNQSH
EYLSFIPTTAYLGPCCSFNYNPRNASFVPFSANIFGMDGGLTFVGAEGSERNLNTGLIVLVHHPMDYVTE
AAASVTITAQSESFVEVSPTVQSSSVEVLELSERKRDCLISGDLQLSNYRQAACLLACQTEAIVKKCGCH
PYLLPIVGNKFKECNLNDTFCYSANYDNFKSVRCDQCLPNCYDVTYSTLSYKTDLNQHKYSVSRFYSPEL
LNNDSFVLRVYLAKQVVPVIRKVTVMSWIGLLSDLGGIFNLCLGLSMISVVEFFYYCTYRLYINYQLQKV
QQPRKAWQ

>PPK7 NP_609016.2 pickpocket 7 [Drosophila melanogaster]
MTLVYFPPSKLQQQQQPSRSSRLAQQLAQSSWQLALRFGKRTTIHGLDRLLSAKASRWERFVWLCTFVSA
FLGAVYYVCLILSARYNAAHFQTVVDSTRFPVYRIPFPVITICNRNRLNWQRLAEAKSRFLANGSNSAQQE
LFELIVGTYDDAYFGHFQSFERLRNQPTELLNYVNFSQVVDFMTWRCNELLAECLWRHHAYDCCEIFSKR
RSKNGLCWAFNSLETEEGRRMQLLDPMWPWRTGSAGPMSALSVRVLIQPAKHWPGHRETNAMKGIDVMVT
EPFVWHNNPFFVAANTETTMEIEPVIYFYDNDTRGVRSDQRQCVFDDEHNSKDFKSLQGYVVYMIENCQSE
CHQEYLVRYCNCTMDLLFPPGQYRSCRAQDLLCLAEHNDLLIYSHNPGEKEFVRNQFQGMSCKCFRNCYS
LNYISDVRPAFLPPDVYANNSYVDLDVHFRFETIMVYRTSLVFGWVDLMVSFGGIAGLFLGCSLISGMEL
AYFLCIEVPAFGLDGLRRRWKARRQMDLGVTVPTPTLNFQQTTPSQLMENYIMQLKAEKAQQQKANFQNW
HRITFAQKHVIGK

>PPK8 NP_001036260.3 pickpocket 8, isoform H [Drosophila melanogaster]
MRVPRNRQRAFFASQQKRSRPQHLQITLSTWRRILSRNTDEFCRNTTIHGLKYINNSKLRSSDRLFFGIA
LLVVLSLAIYLIQDAFDKWNTNPVIVGIDPELTSIANEPFPAVTICNLNQALAERVEHFSNDSVEFAMLQ

LLCRRKVDVELVKTNDSRWEEFILNISQPCNSMVIHCRFGADDYECARLFHPIVTDEGLCCVFNMLHPRF
MYRKRVPYSHRNISLPEGFHAVNWHAELGYRKRGFQPDGDNPLYPRRAQGTGESLGLSLTLDVQADAYYC
SSSSSIGFKIALHSPNESPNVRETGVLLAPGMETKLRIDPSKILTEKHLRNVDRRSRRCLFHNELKLRWF
AHYTQRNCVAECLSGWLIRHCGCVTFYMPRLNANDTICPLHKRECVELIRFRTIIAMESCLDECLPSCFD
LSFSAIAYSTRISLDGFRETPSNGGWNFTDAYVERSVAVVNMYFKDPTFRANKQTEFIGFSDFLSGVGGL
MGLFLGFSFLSIAECVYFALIRPCRTCSEIRQLNQPRVHQMNLKRSPTPGNIRYISAADWFQAELAHQQP
NNRQHFNPA

>PPK9 NP_611622.2 pickpocket 9, isoform A [Drosophila melanogaster]
MQPRIDDSSPSAAARTLTSSSSRSSWAENEGSASGSDSLLTSSTASLLPPQDNNRRLGPGPGLLIFPGTG
QRHQHNPNGSTGSKTIAKANDGNAAINQPGPARSIRSFRRPPLWQPPPQDHAQDGQEMEVLDRQKRKDCR
PACLVKGQAFIIEFLQGSSIHGFIYLAKLGLSFVERVLWLAFICVALFSIISLSKRTWHRFQTSPMVISM
DRNKLVWNTSFPSLTVCPHKRIDELKVEEYILAHPDQFLSEEDQEDFRDFIVKLASLTYDNLETLPLNKS
YGIPSTKYLELLYELKWAFEPEISSGAAVKMFIYETQTEFGICHSVNSMVARYNSFDYWRSGDWSLMDHG
DRVTVHPLDGEIYAQIINLSTAYDVYFHGAGDVPSISKQRYTFPESDYTTVELIALEIFTNEEARATKQR
SCRFNYEAEEMMTVPIYSFGLCLSECRMFFALRVCGCVPHFYRNRCGYNHIFGTQMRNGRRLPVCGLEGI
GCLVKIKREIISLKSDKYKINCNCLANCDDSNFFVQSYRSRVWFLGANLQWGILEHPKMQLRREVLFSFA
DVLVYIGGLVGFFLGCSALSFTEIIYYFTARFVRRMFFD

>PPK10 NP_001033894.1 pickpocket 10 [Drosophila melanogaster]
MVAKNSKYRNGLKIVINYFVLYFNNCCIHGFRYLTDSMLILFEKFLWLILLIASIYFCIIVCLSSIDRYY
TKSTHIGIERNYIFWNTTLPSVTVCPVDRLNITYFADYCRNNGIKGPQRDILWDFLENLANSTYINFQNI
PQNEQIDQIIEDIGLKPEHYTELIYNLTYDRTYEPNFNERIRCMDGAMFIHVRQVLTEWGLCYLGNSKLT
EEYSSRYFIFGKYPEYNKYEYENIRLPYQVGSFFQKDTQYALLGFKGPAIIAFAHSAFEVMKVDSNSDYA
YDGILYDLSTEEITAEDNLEQDTTVAMRRCRFPHESNLTHFPFYTRNICQQECRINLAYKICKCIPHFYP
NRIANPKPVCDYKTLRSCFPHHASFFLKLYEENGKHENPAICYCEQNCHDSVVTMKSMNPMSGAKQLLGG
IGSAVSVKTWPQSRLRRQVIFSLTDLLVSIGGTAGLFLGFSVLGFVEVIYFFTIRIVFQILGYTL

>PPK11 NP_001334672.1 pickpocket 11 [Drosophila melanogaster]
MSDVPGEDSPTHFYPVNFENYLRPKQSIKCQPLQRFKKPNERATNLYRNLKRLKILRWYNRVSKRFEEFP
LPKFLGFLQARNDDGLCKRKTGFEIYCEMASIHGFHIFVGAKTWQRILWWLLICNAVLLSFTLVIMSLSM
SKETPTIRFIDTMMKPTAEVPFPAVTICGFNTKEWMNSSQIVNQRNASWLELLEDLALPICPQIKICQWD
NRMVNCLDQLQPIWTLDQRLCCSFNYNKQLFSSYLGVSFVLRSNDEILQSSKSAGFEVLIHESHEIPNGA
TPRVFVPGESDAHIMLRPYINRFTKNLKGLSLQKRGCYFSTERRLILSDVYNQINCLAECRTESILKSCG
CIPPKSPIEKSWLICDLKQMQCVIDFDHDEIISGEQKNCDCLPPCEFNRYEFQSDIRFIKGMINNSIVNT
SNQETTNEVRVRVYYDSAIAEELLLDVYENWLTFIGTFGGITGLFMGCSFVSVFELIFFSCVRPTCNWLT
RQQILWRRRRNQRVGITESRSLGPAN

>PPK12 NP_611672.1 pickpocket 12 [Drosophila melanogaster]
MEPSPSAGTERQGSSLFLLKSFVRSLRQFLNQTSLHGLKFVGDSGLSSWERSFFLGSFVTALIITVHLIS
NIYVKWDSTPVIIGISPQATSILKVPFPAITICNMNQVQRSLVANYREGSNESALLKLLCESDSWESSEA
DEEFSASNFVTNNLKISEFVSNHSQSCERMLLFCRFSAVERNCSQLFQQILTDEGLCCVFNFQPPEYLYK
PFANNNRNLTNSDGFESVMWDPESGYPEQLPPKFYPATASGTGITLGFTAVLDAEMGEYYCSSTNGPGFK
VYFHNPIEVPKVKEAGLISAIGYETNYRIEMVRAEAVPAIRSISRDGRQCLFKNEKELIFYRIYTRLNCE
NECLAAFLYDTCSCIPFDHPLIYSNASICSMGDTSCVRRAQRASNRPGWAKCRQQCLPSCFDLNYLASGF
SFPLASNNFQLANALVESFNKSYLSKNIAVINVYFRESVYYGNTKNAYVGLTEFLSNVGGVMGLFMGFSV
ISLAEILYFLILKPLAELFVWKRSSHVDSEKSLKHNAFGIEKGQSSDNPSFWHSKELYPKGVSISAYENA
KSTKGLNYQ

>PPK13 NP_001014495.1 pickpocket 13 [Drosophila melanogaster]
MRFSRVIEEYLKNSTLHGARFIVDKDASWIERIFWVICLVASWYASWLLIKASLSAFENNAISFVVESSF
RDWNTNFPAIIVCESKNMDRIQEVAEQLWGADHDFTLEEVLSEIAFFRGESYHTVHECSGEEVTASCFYS
NFSYYAELVRSSCVDSISNCEWNNKPFECCKYFHRMETELGICYAINSMQAGKPKMPKLNMYSNRGSGPG
ALKMELHTEATVFILGTEEVPTLVTPKTDFLVVGPYISFVRYISKRDIENDEEIRQTSVHQRNCRFADEN
ILDVHKFYSYSACTVQCRKDRQMELCNCSSHLSPNSPDWQLCNMEGLECLNRNYEDLSVIIAKWSKLRGR
KGLVCDCLPSCTEVDITTVYDSKENMAGSQNAISRIEVGLIELPTERYKRNLVRGKLDLVVSIGGTTGLF
VGASLLSFVEIFYYITIRPYTTYINEKRRLKRLIIPKRFQ

>PPK14 NP_609017.2 pickpocket 14 [Drosophila melanogaster]
MFVRSTEKETRVVADRIRRQDQNPLAPVNTKSEIQRAWTLLIDSYISRSHIHGLYLLFLPSMRRRMRVLW
ALALICACTVLFHVSYLLGDRYHNKQFQTIVAHAHASIHHIAFPVVIICNKNRLNWSRLPEIKSLYNITP
SQDELFDRILTAYDGFSFHKFNAFDSLLGESLDELNHLNFTEIVIQMSWRCDEILRDCHWQTASRDCCKL
FRPRRLPLGYCLAFNELEKRRGTETGINTGLLLRLLLREGQHAPGNSGLKGFWLTVVESSVWFGFPIEVV
PHSRTNVAVTAVYHYFDESTLSLPSSWRHCVMDYEEESEHFRTLEGQKYMLENCQAECQQRYLLRYCNCT
VDLFYPPSNYPACRLKDLPCLAAHNHLLQNFEQPGEHPYVHREESGLVCECLHNCKSLTLLTDMRKSVQQ
PWLQPNSSAIESMWLNVYFKKPSMLVYKTNLIYTWVDLIVSFGGICQLCLGCSIISLIEFVFFALYKVPQ
LYWERFSNEHRSNK

>PPK15 NP_001097937.1 pickpocket 15 [Drosophila melanogaster]
MGGTENAKGQVLRKKRGFGFVTNIKDYCTNCTLAGFAYIANSRLHFMERIFWLICVFMSSLGCYQLIMGY
QRSFPTRAVSIVYESLPPFSKWKFPAVSVCELAYRGNLFPKFEEYITSLGVDVTGDYPYDVETGVSILLF
PALYNENGLKGKCGTVHKNCTDACAKCPSDNYRQILTWYGANCSDLFVECKLSHEPFDCCRHFLPLLTPF
GRCYMLNSLLNNEPGSKHWLPNELDPAHQKAVINVITRLDVQISVINAEDIPHTAFFPPGIPLITEGLSK
YMQFNQVAMKNDPDVKDIDPKIRSCFFPEEIPADSLYKSYSFSVCITECIRRLQMKACNCTSFLYNPNAD
PRYPDCDLEGFLCLEKTRMIKPDSRVLVNNNKGNNASCGCLPSCNDGDITTIYEPLLFVRNPNKYYNGTL
DMPFLPTDQYRRQSLRTPLDVVVSMGGMLGLFLGASILSAIEFVYYFTVRPLSNMLGARAARP

>PPK16 NP_001334673.1 pickpocket 16 [Drosophila melanogaster]
MAFKKRRIFDLRHVQAQQMATTAIHRRSHPMHNHSHNPIQPQHRFRQIGEWFTENMRNYCQTTSLHGFSY
ITRQDISRHERWFWLVVVILAIITSIVLVVVSWYWSQETPTVTVIESSHFPTWNIPFPAVTICNFNKISK
SKALSLLDQMQVPVGINRSELHNLFNLTLLPVDTMISNDSLQKYDRILSLNNLTLNRLTQQLSPDCIEMI
SSCIWKGINTRCESLFQRIDTMEGQCCTFNYFGGISNNFPEKIAYQVPKRPYRVTGCGYPTGLSVLLNPM
ISDYYGTFFSGFGFRLLLHDAYNFPDENSETKVVTTTRESFVRINPESTYATNDIRRMDLSLRNCLFGSE
MTMHGLRRYSFINCMFECRVRMTVDLCGCLPPYVYNNGSYKVCGVLQTNCIIHSKRLFSHALANLNFSLS
IVRETDSFPCGCLPDCQSNHYVSESTTGRLDISYFANRPTFNNATDRILLHVFFSDLMSTRYRTDIFQNW
LSALASFGGLLGLIMGFSIVTAFEFIYFLTFRPVFNYINRE

>PPK17 NP_001285988.1 pickpocket 17, isoform B [Drosophila melanogaster]
MGYWSETRAWLFRDPAKLIRYLVLFACCIVVIVQLYECFAKLYNPPISTHSYYSLNETIEMPSVTICREP
PYKEEVLTRLSGGACPHPKYATCWMKYPFGEISLDEFFENSTHDSGDTFVFYGLNEDKNNVVMNSSLHFY
MGRCYTLRPKESAKRVSKAVGYSIMLEHSMLTTSVSDVDTGSVGWHVFIHDKKENFTEINMKGSGRVEYV
FVGVNEEIEIKLQTQYFSNVQTREEACSDDENYSDLKCGEQCIWQDLADNMQCSGPWMHEIASEPCNDSL
SMRKLISDYKDVYENEDDFDCDCVQPCQSRIYTTFIQNRKAFNQPEPRTQIYIYYTTKLISMIEERPSYD
TTQFIADVGGSLGFLLGLSVLGLIGILEHMMLFFCGGFIKRMQQKEQAKLEANSEDGQSQTSDETIDVEI
AYKKKEKQPKY

>PPK18 NP_609308.3 pickpocket 18 [Drosophila melanogaster]
MARVEESYYRRLFREFLRQSYINGLHPFLYHTPVRYAKAIWLAFLTAIMIYTHIVIADLILEYLVQPTEI
HMAPDLVHVANSPFPAVGVCTSNKINGRLLRSYAEKLFHHQGNSSLQAVALDLDDMANRLLVLAQFYLNP
LESDHERWNATELDRLHRLLVAYHNGAGYSVRDILRDLSPGCGDLVIRGIVFGLPVNASQLFLKRPTSSN
VCCIFNYRRPSYSQFLTKRAEEDSEMRAVPMVVFESNSILNSVQFVLQDTPKEDITNTFHSNSAFQLIIF
PQEDYATIQSTTLGEVLVDHYSIVEIPIQPMFFSSGDGVRGVPPHIRRCYYPEEGMRLLNQSYYSLDECL
LVCRMKSMIDHCGCVSPPLTGSSMDLDYCMLPDLPCLMRWKSIWYGYSDFAYLQNKEENLVQRQCEHCLP
TCNGVSMSITTNVAPLRRTYNSSGYFTGLLKGLTNERPLAIIKLFFKLRFAQATKSQMVSSWVVLLNRFG
GILSLMYGFSIVSYIEILYYLTGKGFVYIYRSFLLDKEPINHNYSLYWRELRSNLPRQEQLRK

>PPK19 NP_651708.2 pickpocket 19 [Drosophila melanogaster]
MLLYTKELVIPRPRPGLLRFRNNPRGIKFREKLCNSFAHSNIHGMQHVFGEQHLWQRCLWLAIVLGAVIT
GFSLYTVLMHRHSEQLLVSLIETTQLPVYHIDFPAVAVCPWNHFNWQRAPSAFIRFLPRHPNAELRETFR
QLLASMDIMNFSNFNRIRILTKRNLTGISYLKMTDLMNFMTYRCDELFVADSCVFDETPYDCCKLFVREQ
TVKGQCLVFNSMISENSRKKHLINQFYPHKLSTAGEDSGLKFTINASYSFMNNIDALTPFGMNLMIKEPR
QWSNEMMYHLYPDTENFVAVHPLVTETSPNTYEMSPKKRRCYFDDEKNPTFQNTSLTYNRENCLVVCLHL
VVWKTCQCSLPAFLPPIDGVPECGINDAQCLGNNSDIFTYVKMGDQEKYINDSRQGHFCDCPDNCNSRLY
EMSLNVRKLDYPKNSTDQLIKAQVYYGQRVMTKIITKLKYTNIDLLANFGGIISLYIGASVMSFIELLFV

LGKLMWGFIRDARIKLKEYTK

>PPK20 NP_651705.2 pickpocket 20, isoform A [Drosophila melanogaster]
MAKGDNSVKPTEAAGFGDNERCLADLLKVHFRSYCEKSTIHCVRYLYDSHLHNLERFVVYPLFISTFNFK
LYIIIVFRIIWSMLLIISIGLSFFFYLLLSERFVSQKLQTVVHDPQFPVFLVPFPAVGICTDNRINWNKL
EAAKEQFLPTNASVELVESFTVLVSRMETLRFGSYLSSLAELEDDNLEAVGFVNLTELAIFMTLQCSDIM
VPKSCLWRSSSFNCCEYFVLEKTEFGFCLVFNSEVSPRSKAIKQKEGNNFYPRHNAKAGQSTGLNFDLIL
NESFRRPDSQANNNVYVSICQAPDQLNNVVYSITQNTETYVTVRPGLTWTDNTTRSIPPERRNCLFADEQ
GELDANDSAKNFGKPFQLSNCLNRCHESYLIQLCNCSLPIFFLYNHRVPDCNAVSLRCLARHNDIFSYDK
RRDEDALFSATKLGMTCSCLVDCYLLDYYTSTTTLPLSAHKLPKDPHQKLFRVDVHYQVETTPLYRTSLE
FTIIDLIANLGGIFGLCLGASMVSAFELIYYLTVGLAMHLYDHQYYGVLFKHLKAKWVNLKGYLRNEVGH
LAENPAAHKDTNDRKLRHPFNYRKNVW

>PPK21 NP_651704.2 pickpocket 21, isoform A [Drosophila melanogaster]
MLYPLELPRARRPLYRDYGGKSGLIQTQRDARKNSRLGKLWHFMLPYLKDYAAESSVHGIRYLADPKMRN
YLNIRVIWLLILLTTSIGAIVVYVDLNELYQTVRIQTTIKNTMLPIFRIPFPSIGLCPRNRLNWKILETE
AVDHFLGANVSAAQKDLFVKFFTAAGDPHLSRLNEMSNFFGNKTLTDELHMLDHLDLREVYKFIQFRCQD
LFHTCRWRGNPVNCCEVIEYQFTEAGLCFVFNTEISPASRQKAREDKYYPLRTPHYGEGSGLDLFLRLNR
SFIRPGKRGINVMIKQPQQWSDVVRHVPHEAHTRISITPRFTVTDERTRTVTPEIRRCIFGDEVDNPHYK
NFPDFEYWVGNCRSRCHQEHVLNLCKCSPSIFFPISDKDNFTACKASDFKCLYDNRFTFSIERHPEEDDF
VKNPFKESMICDCFTSCSQLVFDRVFTTTTLDNNETDTEAGTMRLDIFYQSGWFIQYQTNMRFTFVELLA
SFGGIIGLFLGASLLSAFELAYYFSIGLYLYIHGKRKLRKPEPGVLTIQFGQRKITPIKF

>PPK22 NP_733051.2 pickpocket 22, isoform B [Drosophila melanogaster]
MVKLASTSNAIWWIKNPKAAGEHNSKGKRKESLGSAFCLDMADLVRNISLQGYNKLLSPDLTLGQRLIWL
LVHMATTVSLIVVLSLTWEQFVAQSFVTNLKDPLFPVENVPFPAVSICPNNRISRQAVIQYAEELRLNSP
VIRPVEYFLERLRFFREFYTHVGVVVDTDDFITFQTFLDVFGTWNNETFFDTRRIMKMLTPRCQGFVLKC
TVANVEVPCFSKDAFQDSLTMYGPCCTFNMENKLKKRHFKNRLASSELGLKVVLNDSHVDYFAPILNTNG
YIVMIHNAENYASVYSSNVLEMFPGQGEDSYIAVCARVVDTDDSLKSFSPFSRRCYFEYEAQNPIHEQLM
NTYSLSYTFPNCITRCRIRSIIALCRCLPFQMPLQLVENLDGVVYCTLGHVSCLNQYIFKWRNILTERHI
VNGLEREIEEALYCPQCLPSCRDVQYEVSMSALPIDNYLATLKLDENNETEFGTDISVLRVYFGDPHAQY
YIRLLNNTWFEVFSTIGNIMSIFVGFSMVAIFEILFFVTKYIYKGCNRMVEQNIMDRKAKELETKKLYIC
P

>PPK23 NP_001014749.1 pickpocket 23, isoform B [Drosophila melanogaster]
MPQEKRHPPESRTQRFLETLVIFRRSLIYQTKEFFQNSTLHGVRYIAESGRPIGEKFMWFCFTSIGAVTA
LVIIMSLWEKFQTNPTITGLDTDFHNQNVVFPTTVVCPEAAFDHDKTYEKVYNTLANYDEAQAQMYTPFL
RILTSLNFENVRDAKVLSQSIPQNLLDAHTIREWAFEGHIDCKNVFVSCKYRDEDIPCCDHFEPIYTEHG
FCYAFNSRFKSTPTEDVKTGAPHDLYETDKKWALFFIPNSTSRIFIFSNEEYFGSDFNAQIDWSEPQLVE
VRISKKNTYTTDDARQLSIGQRKCIFSDEVKLNYFPDAYTFSSCMKQCRMNKAIKLCKCNPPFYKPIREL
SCVINIFTNLIAYILLLYLTPKANVPMCSIKDFDCLDEFKSNITNIKDCLQCELSCSKTVFNIDKLIKMS
DRPESLGVLVEFLTWPIIRYKREVLFGWVDLLVSFGGIASLFLGFSLLSGVEIIYYFTLRACCMVYKNRV
NYANRIFQSTSI

>PPK24 NP_651860.2 pickpocket 24 [Drosophila melanogaster]
MPVEMESLDQKPFRLAVGRAAWWIPNPGRVARYPPIRTLAPNHLPGTSAEDTRKLSFAAALKDLLQNLSF
HCYSKLVESGRRIQERFFWFVFHITALTVLIAFLWGTYTKEQEALVTTMYHPMYPIWKVEFPAISVCSLN
RISQRAAWQYAHNLSGKDPKQRNASHFYDQLKAFMYMYYDPSDLMDIDNALRFQSFLDRFDTAKEELFFN
TRNRMSALTPNCSDMFVSCRIAGRLFDCMDKFETTLTSHGFCCTFNYDGRYVNKRDFRQRYFGPDMGLVL
TLKTDPSDNFYKINGHNGYPDPFSGGVAERVAETGFNTLLPVRAKIFETLPEARSMSPSVRKCLFENEMP
WIFARHYTFSKCISACRAQSVVSLCECVPFSLPHRYIDGSEKRIYCTLQHLACLKRYEFKWLNVITSREN
VTGLEHELQDALYCPLCLASCTETRYSVRGAMTLGLPTPSQIKGPARSNPGPRANNSYGPASSSGSAKGP
VHSSSAPAELAVVRIYFAETHIQYFRQIIKSAWYETFSTIGNICGIIAGFSLIGICELLFFLAKQLWQAC
RAELRAELAHIHVQIRNAQVAEPEAERPMKLFILP

>PPK25 NP_995766.1 pickpocket 25 [Drosophila melanogaster]
MESKMSRPKQPDWLAELCQESSIHGMPYIARRDLHWAERLFWTFIILGSAYYAISSCLNQWYRFRDNPIV

YEYEYLFGLRIFPFVGITLCPRYHDETEIPRLINQTWGVDPSEDKEKAVYYRKFLLAINGLRYSTLETLE
PFENDTTLDNVNYLNILLTLQKKVIAVKIPPELAPIITEVGLCQTSSQLNRYGNPYGKLETQDMEPMKQC
GYLSNCITSLKPINSIVAPIFMYLHDVEEMMLPDDMRTPSFDAKDIESKDLDIMLHTTSAESEVRNLPVA
YRKCRFSDENNLQYYSPYHPSLCRLECRIKWALSLCNCKPYFYVAAPEVPICTVSGMLCLARSKWLERPC
DCYPSCREETFTIFKVSDQTGGDDNYSGERFERTLIINLQISRMGINRRVVFSTDQLIMSFGGAIGLFLG
ASFMTIYGVVYFFLTFIAYTCKNRFCKRFFF

>PPK26 NP_648125.3 pickpocket 26 [Drosophila melanogaster]
MSTDSTQPHTPSHEVAVRPPPCAANQWKRAALMARMAPAFRETVSDDSEDEVDTKKQIKYGSGFQAAKAI
IHEYCDYTTIHGIRYLGEKKRPLLERLFWISVLVLSVFTCVKLTLNIWDKWNNNPVIVSFAEKSTPVWQI
PFPAVTVCPETKTRREIFNFTDSYHQVRDFQSNVSGIVDLSDKQKGLYGAVSQVCEPHLHDVTLGNKTRR
GMEIIDALTEVSPHFDDTYLNCKWRNSPVKCSDIFHKFVTEDGVCFSFNSLSPAEIFRAEGIIPDFIFRE
ENRLSMDWNVEDGYSASADTSPYPNRVLGPGARAGLYLFMGGAEIDFDDMCRGPVQGFKILLHTPGDVAQ
VSKQYFRIPFDQEVLISIRPKIITTSDGLKHYEPNRRQCYFQKERELRYFNIYSQSNCELECLANFTLTK
CGCVKFSMPRNVNMPVCGDASLKCYNQAEDELLLREFTQGLVNAGENTRGETECNCLPSCTSIAYEAEIS
QADFDYKTVINTDSPEGKEEQSKRQGMKMSRVSIFFKEAQFLTSRRSELYGTTDFLANCGGLLGLFMGVS
MLSIVELIYFCTVRLISNLRMRRKTRKELLKAVNKDA

>PPK27 NP_647826.2 pickpocket 27 [Drosophila melanogaster]
MSRIVTAFNKTVVEYFRKTSLNGFGLLYFIRKRRIQRIFWFLFISFGILFASYAVFSMVLEFLSYSTIAD
LSELKVLEDEIHFPELKICSGYKFSYRNMLASAHDLVSSQNKSLDYWLNKLSLLSGYFDALSVKAENVDD
LNSLLDIKNISSFLLALTPACESLILKCKLNNIPANCLKLFTLKAYNDGNCCVLRNSNLTGELTLFMDSS
QIDEYPLNGNLPGFSLHVPSWQGRVSINPGEMAAVEIEVMELQGNSQLNEYAVEKRACYFSQEGESREKC
LHECRIKATLINCQCVPYPFEFRTQKFGYCTLENIRCLQLVERNWSPAQCPQCLPLCNQLFYRLNKQILG
HLHPWRSELNFKFKTPHRQRYKTNILYHWYQMLSNVGGVLGICIGCSFISGFELIYFLVFRLWTNYLRQP
ET

>PPK28 NP_573169.2 pickpocket 28 [Drosophila melanogaster]
MRTLTESRRRQSGSSGCKKDSESDDDENTICSRAAIKRSVVYYLKNSTLHGLKYIAEESITIPERIFFGL
AFVLVVILSVFFISNVYVKWSASPIIISTSAKQKLTSNMPFPAITICNLNQALLSKVDRIGRTSTNFSLL
MGLCDQGGDTTISYIGTWKYFKAILVDVAQPCEKMLLYCSFGSREEDCSWLFTSILTDDGLCCNFNALHP
SYLIRNYSDDVRLETAHPNTRYELIDWTPEKGYARNLPEFYFPRTSGGTGIRMGLTVVLNASIAEYYCTK
SMSVGFKVLVHNPAELPKVSNYGFVVTAGREARIPIEPVYEDALPTIRSIKKSVRRCLFSDENDLAYYRT
YSRKNCELECEAKLLLRECSCVLYYLPRIDPLARVCGPNDNQCTDRVQTEIESSLTNLSCENCWPGCFEL
TYRATLSTASIVSDPRFQAGENLPEYIFHGPYSNASEISILHFYYMTNIFRSTTKSEMFGFTEFLSNTGG
LLGLFMGFSIFSVIEIFFYITVRPYCASRTLRQRHKRRLEQLSWLTPIRMPVRRALRRNRGGLLRNPPPP
AYSDLQKFRGKLDKPETCKRKLWRTLQVRPVVDRADEELPTYPYLD

>MlNaC1 evg198836_CtenoMnelei
MITSGDWYRDERALVNSAYQLPVRHINSTGTSTDNTAPSDPPIQYDPYQVVNIKSRPSTPDIRLFRTKNS
DDAPSSNHSYAPLSHYSHPWTLSHNSYTPSSNLSHFESSNERDLAGSRSGAHSKAYSMDKKKCSLFERSN
SCVEAIPYIGSDRDRDRSKTPERSEKRNRTPDRPQHKNSGKTPENQRRDRAKTPEKRDRAKTPEKRDRAK
TPDRGRRSKSRTPDRLRHRSPDNYSERNNRADRRARSPPQFPAAMVTMPTSSISTVPYPPLPDRIPGSTR
QEEFPAAERVQVAELPLPAKVQVVDHPLSDQVDLSAGERRETEPFVVGRSDTIKRADQEPESSDSRYRDE
MRKAEEAEKYQNSSSSSGKRRSRQQDSSQDNRYRVGGSSRHGDVRMRAYSDSDAENYQEAGLNGHNFRHP
LNFRHRDTDRNGGQIPPLVYTSENTDRFYRIPYRIPYNNRPLDSYDDSDYDNLPGRPTSKSNRTNGALLM
PSRGARGRGDCGQEFNTRDPTRHPDLEYRPYPARFAPPEPACPYYSETTENEYLTPAEQAAANKGSRHRR
SHSDSSSSRTIMSPWSKDNRILTPKYPLLSTRDQTLTSQDRTLENASQRLSKVKCPVSSNNGNHGNNNMW
HDPLLGVDKKHIALEGEVESQQSKGDSKAESFAEFTTEVSMNYLRFIWNSPELIRKLLWAVLFLGFFVYS
FQCCLKSFNTYFSMPTSTKYNMYFEPNKLLRFPSITICNMNPHNTAYLDKEENNPMKAYLLSESRVPWDT
SNLTPDVSETDYRNFNVKKYYRQAGQKLNDFISEVVFFEEEIETSDAFREQFTTHGMCWTFNADGSRQVN
RTGMDFGLSFKLNINQSSYPNFVTTAGVKVMFHNYYEPALIDEYGLALTPGTENFISLSYERVRNLKQPY
GDCRDDAMPLYRNYSISGCERQCFTEIMIKECNCSSYYLKAMPGGKDVECNLYQEKNCVKELIRRYHAGE
FINSSECRCPDPCVSESYLATLSTAEYPAKNHGEALQRYNEANRGPNDVVQDVTFYRENLMKVHIYFEIL

SIKEVEKVGSYKWSNLMGDVGGQLGLFLGANIVTIFEFVDFYLRLLWYNIIANSYKRRRGDIKLKPTTKQ
NSSFNPASIPNDREQDISPLITSLS

>MlNaC2 evg118785_CtenoMnelei
MLQNPWPSLERQNAEVNLLTPNRRIKCPRCRQHFVFNKVRKDGIMGWLQDFKEFSDGTTAHGVKYIFHPS
VHGNFKIVRILFLVVWLVAIAYSLFVIYNAVGNYVGKPTGTKFQVIASNPYKEKPASIQFPTISVCSHNL
VTKSYFKSNDGLEELWSELDQWNPDTAENIDFNEGSPAAKYKDWTYEKIIAEGGPANWTFLQCEHFINLC
QEVIPQPDFFERETTLTGNCFRINPNGKLRGKGGDYGKMQLMFFADLNEYSDLTRREPQYGYTVVFHDHE
SYSSTIPSGFWLSPGSIYKVDLSLGKEFREPPPAGSCDPTRVNNTYGRYEENSCIAQCRDDVLMEKCGCV
HVSPPHPRNVPDKYYRGCTLEEWATCGLRAYKEWVVEYSNVNKVKTECNCPATCTEIAYKAQLSSSQLSK
FYAEEKAAKLPPGYENAQDVLENLLIIDILFTSMQISEIREIVTYGWGNFLGDVGGVLGLFLGASMFTII
EFIQFIVCAVLKGCCGLNDKESRSHSPLYNENL

>MlNaC3 evg1172868_CtenoMnelei
MTNNFDEELREFALITSAHGVRYIFHGHRIVVKVIFFLFWISAVLFCIVRASVSVRKYMEEKTTTLYEMH
PASASAIMDFPTITACNYNKIRKSYLATKPELKDWFQGISLFDFDQLENLNWESPELKKFENITYADLLR
EGEMYNDTFITCTQGHLRYCHDTDMGDGYFSRDVEFVSGSCFRINPKGNLKGKSGDYGVLSLILIADKDE
YIDTSHNIGWLVATHEAERYGASIDNGIKISPGQAYYINLDTMIIHNHKKHCAATEGKISGYGRYDQSTC
LLDCRDRMLNKTCGCLNSTPPLNDVFNYKECTIKQVATCSLKAYYQYVLDFADVKSDYEECGCDVVACEY
TLFRTTVTATPLPDTEIEHKLKAWNSPRHFDRFQRFSNYTAEDLAKNMARIEFFFGSSSITKVEEIISYD
FDNLLGDIGGVMGLFLGASIFTTMEFCTLVVNLLSRLWQWKIQPWLRSETRDRKIISETNL

>MlNaC4 evg1200311_CtenoMnelei
MSNRVITTDLRLACQTALTPNTMPETKIKIGLTRGLVDGGEIFAQFSLSSTAHYQHLLTYISAICSREKV
LAIYWFNRHDREFVLIHDNASMDYCLVQSGLHEILYIITETGGKPAGYNSKHKPEPIQLKTSSPSSGPSP
DTANLERSSDEDRPEETVQREDHIPLRKMKKSASLEWSYDNEQPPSVQEISSRDESAFDMTGRGERPTYY
PYLEHYQPSYPARPKPHRTPTRSKRPEPKVNNKYHTHSDQSDDDRNAKEVDQFMEPEPPKRKKKAKRRAN
RAALEQKRQEIEMETFKEFASTVTADYLAEMCSKASNGRRLIWVSLWLASFVYAWYNIALSIGVYMSKPT
ATKLNFYEASSDGVTFPTVTICNFNKFNKSYFDTDDITTKKGQLKDFLKITTPIWGHKQELDSASWDTKY
DKIVQQSMKDALQTASHSLEGTVEFCKFSGKPCLPADASPGDVADVEDADPTAAFNPVFTEHGLCYSFNQ
NGNLTMSRTGALYGLSLRLHVAQDDYFETQDVAGFKVLLHNSYEPPMIEEYGFALRPGSETYVRIRLQKY
KDTPRPLGYCDPYLEDQFSTNYTISVCVMQYRAINMLKVCGCITFYMDRPDIVLSEGKPYMKPKICNLKE
ERTCADNVLEGLALGTLKIDGEKVPEPDCPSPCVYTTYTYTVSQSEYPSSAVKDKVVEEVNRQKGTSPGE
DDASSWTIERIRDNYLKVHIYFEELSTLEMERHPSYLISNLIGDVGGQLGLLLGMNICSLVQFTDYIVRF
SFFKGFMSLVRLYKARRRNK

>MlNaC5 evg1156512_CtenoMnelei
MTHPNMPEEDYKLRAVQVIQNPNVDLTSTSKPPPTSTPCQNSNVGKQVQSPPSDVKSSKTCMKMSWVKDF
REFANDTSAHGVKYIFEGRYKLVKLLFLVTWLGFSIYACHVIITSIVRFVEKPTSTKYEVIQDDSEGRPE
RIEFPTISVCSMNKVRKSYLEAPENEAIREYYEVVDKYNVSLVKDLAKRFKNSDDPLHSIKDMTYEELIR
NGGPNPDRLLKCTQRAKYCHELPAFNGRDVSVMENSMTGNCWRVNPEGRLMGKMGDYGAMKLMFWADVQD
YSARTADVENQGFVVAFHDNSTYGSTMTAGFLMSPGTYYKADLRRKQEIRNRDKIESCNASLTENTYGAY
NEGSCALECKDEALHKACGCTNVVPPLNNGKYKSCTLEQWVDCGLAVYKEWFHNFTDTDRADQLCPCQIQ
CEEVRYEAQISSSSISPAFAEKLFPSVQPIISQPQYGYSNPDFNILYNTTQDILDNVMVLEVLFTSMRTN
EIKEIISYGLSNLLGDIGGVLGLFLGASLFTILEVFQFVFFSISKYCFDKGQPKHDSLTNGDKLSI

>MlNaC6 evg1125780_CtenoMnelei
MLLNNDTKPPDVPKKQSFFEDFKEFTTETTSHGIKYVFHAAYKWVRVIYLILWLAAVGYTVSIVFISCKK
YFSMETGTKIEEKHTPKHSSNEVAITHPTITVCPNNLISKSYLAKHPNLDELMTKLVTFNENETPSLFDD
PKYAGYGDMTYHDLMWQGRPRVGLLQCTYFAHKCGAVDPFNSEDYETKTYHWDKTYNGSNHKFYEWEVSL
SGSCYRINPRGNLWAKVGDYGEIKLTFFADLNDYSSASKEEPEYGYLVTFTDSGTYSSTATSGFFMSPGN
VYKADLRKYKEENLGPPAGKCDIESKTNVYGAYSTNSCNKLCRDQHVMEKCGCVHILPPHPENDPDTKFV

GCTLKQWAECGLRESTKFTEEFVNMNDDKVHCEHCNPACMEIWYEATISSSRLSTFYAEAKVSELDKLIT
GYQLNEGNKTTNYTIDKDTVYENLMVLKVMFTSIQESTIKEYYKYDYTQLIGDVGGQMGMLLGASLFTVI
EFIHFFIKGFFRLFRNKVNNPRSPPFQRHMDMDSLN

>MlNaC7 evg116939_CtenoMnelei
MEILDHIVYSGRTFTAMEKTPRGMLAKHRADRFPRSLTEQNPTSSDEPEERSPDMKRKKKQKEDKKPKHA
CLRTFTITKWIFYLAWASALGYTLFNISKSLEKFLSKPVSTKISVYHNEDGTMQFPTISLCNLNRVNSSY
LSRDPVHQRVWRIVDTNAPEEIDWSDPPTAKLGDMIYQDFLKKSPSWEQTLLFCAYAGVRRCSDLPVFKD
LSNMVEEELAPTGKCYRFNPKGLLHSKAGDYGSVYLRLNINLREYMDKSSNGFVMAMHHHSQYSSTRNTG
IVMSPGFKYRINIKTLERTELPVEDGGKCNSSNSYNSYGSYDLESCHTECRDRDLNKACGCIPVLPPNNI
HGYRACTLKEMYECGNNAYLNFVLEESDTEHGKEHSCDCWVPCKHYSYELSTATTAISREYAETKATGSE
GKRLNFTTEDVLKNNIILEAHFRDMYVQEISQVPEYSFWNLLADVGGMLGLFLGASVFTIIDFIRYAINY
SYKEYKSRFRDESPAKSKQKASKPKNNESSNGSRDKRIAPVYSGSRKKKTKVQLSPQAEKSRASSSDSEE
ISPDRKGLCEGSTSASALNGQNNVLKLI

>MlNaC8 evg123254_CtenoMnelei
MSESKWELLRTRSVWKGYYKDSSILDDIQEFTSETSAHGIKYIFEGPNRLVKLLFLIMWTGFSVYATFTI
CTSIIVYVNKPTGTKFEVVAAADPSFKDHGIKFPTVTVCSTNKVKKSFLDAKENEIIKEYFDIIDTYNVE
KSLDLGKRFEDPEDDMYSIRNMTYESLLEAGGPKPDRFLMCSQRAKYCNELGAFNEKGYPHYYSMENSLI
GRCWRVNPDGRLEGKMGDYGSLKLKFWADVQDYSERTADKETHGFIVAFHDNETYGSTMFSGVLMSPGNY
YKADLTLKETSYNKDKASSCNASLVNTTYGMYHEGACVLECKDRSLNASCGCVNVLPPENNGKYQACTLD
VWAKCGLPHYKKWHADYSDTKRKESICPCQVACIEKKYESDISSSSISPAYARKLFPVVNVILSNEAYGF
SNSNFNISYSTPDDIIENMMVLEVIFTSMQKLAVNEIITYDMGNLLGDIGGVLGLFLGASVFTIFEFIIF
GAICLSKVFQKLIGRNDREYESLNVQN

>MlNaC9 evg19430_CtenoMnelei
MPNFQPQWKVKDTIIRPVSLIKKIYSPEVFLHNSLEALRSTTSEESRNSNSIRRWPLSKPAEKKVKVNNC
SESNQEDASEDLVEEKTEETKCQRCPLLRGSSTAATIACTNNTDLTIQFLDGVIPPNHSQPFIVVANPSS
NTRPTSESNDRRSSKSRYSKRVSVSSVTCSIHNETLCSVPLENSDSDILEEREDETDPTQEELEKEESRF
FCMVRILWRAFYLAAWVAAAFYSVYNIVVFVNRYFERPTQTVITSVKPAVDHLEFPTISICNMNRIEKSF
MEVNPALKKVWERLEATKNVNWNNKTFAEIGSLTYSDVYDKSFSWRKVVDQCKYGGWRDCAELNYHNNEL
FTDVVGASGKCYTFNPQGTVFSKSVGDAGCFRFLMNVHSAEYLESVSEVGFIVDIHHHTNFQGGSKIEMS
PGYKYRVGIKPKIREELPIKLGGNCDPARSNTSYLAYDADSCEYECRDNYINRTCGCIISAPPDNLHNYL
TCSIRQMEDCGWLAYYYFLTRDENMPKHNDNSEDHKETLKKQFDREKFSFNRVQEKETQRSAVSPPSITL
GGSGHVFTTESTVYPTTTTMPLFVNSNVLNCSQRCPPPCFKRYYEIVVSSSKISTRHAKILASEINQKKR
LGVTADYLLKNHVLMEFYLADPFTEIVSSVPAYNLWHLLSDIGGVMGLFLGASIFSFFAFFKATVLRIYH
NSKERISFCVFLKNLLISK

>MlNaC10 evg1147253_CtenoMnelei
MPPKETSSEESSSSSKEKVKLFGVNDLASDYFQVLHQKGRPKLCRILWFILIIVLTAFTVYLTYRLIYNY
LQFGSFNKSETEWKSGLNLPAISICGTNYLNYTALKAALAAENSVAEHEGGDSLQKEFDDLVTDFRRYES
YGENLTAEDALKAKDLLNWERSKGSITVRFKTDIYDMVVGHFDYIFRGVGYRVEEEQKVEMINPTELGMC
LEINDKEELIQDVLGRNGMFTIDVDAHVKDYLFTTQTLGFVVFIRDYDETVMLNQGGYLIAPGTETFMKL
SAQNVTRLGYPWGTCENVLSKYSKYGKRFESVRECQERQQIEAMFKHCQCIPWYFAERMYTEKRYSVLDE
AVDAIKTSKYSRKRSTDTEDIDQEIKTTETRNGGASESQVFHSNYTEHICTFIQENLCKGLISNEIKNGV
LTMEECPEPCKYNTWSVELDSTAFPPTKEYFEHFIKYDAAIKDEKADFDYARENMARIHIFYKELKVDKQ
SQTPAYDISSFIAELGGTIDLFIGFSIFTVAQLIEIGIAFLVHKVLQRSKSSKRKNESG

>MlNaC11 evg115494utrorf_CtenoMnelei
MTIPPNQRAHDEETVDMVQSKSSLLIGRHFRLLFNKISGTRKTALLRKEMKMRDPAANTDTELWNENSDV
RKNDPTTEVLLKLEGGINTADTVELESESDIPQVRKLEISSLDNCVKSESPNQQSTSSSASVKGEEFNLE
NGRTTSASERVALNSKKKDDKDESETEMYVFDEKGARVRTHSVMEYLTMTWRLLYLSAWMAATIYSIYNI

SLFVDRFLKRPTHTEITSKPSLEEGLALPTISICNQNRIEKIFLDMNPAVKETWESIYSSKADWDDPSIG
MIGNYTYSKIYENSFGWQRSLDNCKYGIARTCYELNYPKDELFTNVVTESGKCYAFNPVGKVFSEYPGPD
GSFQFLMNIHREEYLEETSQEGFIVDIHHHTNFQGSSKIEMSPGYKYKVGIKPKIRREMPIKNGGKCDPT
RSITSYLAYDANSCEYECRDRYVNASCGCIISSPPNNKFNYSTCSIRRMEECGWIAFYNFLTRKENTPTS
GNDRNEPLESSSHDKRHRKARRKRNIDDEGRSQDKTTTSPYSAIQTAQSGNFTTYWMTTQPTTTTHHPRA
YDPASTAHLMNCSEVCPPPCEKQYYDIVVSSAKISRRYAESLAREVWVEKGREISANYSLRNHVLMEFYL
VDSFTELITSVPAYDFWHLLSDIGGVMGLFLGASLFSFLAFFKATALKIYRNKKKKMPFQVFVKDILMI

>MlNaC12 evg125633_CtenoMnelei
MVVIDTEPWAESTKREDVHSIISNRDFFGFQDLASDYYQVLRQRSRPSICRIVWTVTITGLLCVTLYMVY
KLISDFLQFNSFNVSSTKWTNNLTLPAISICGTNFINYTALSADFRHFNTSSGNEDLLEDFDNLLTELKK
FSTYGNVLEYLDLDQYRDLLAWEEEHGSITSNFRTGIYDMLVGEWDYLFRGSGHLIENPAKLVNPTELGL
CLELNDGGNMKQDISGSNGGIVLDLDAKVRDYLFTTATKGFVIFIRDQDETLMLDKAGYLVSPGTEAYIK
LSARTVSRLGKPHGTCQNNLSKYSKYGKRFESVRECKQRQRIEAMVEHCGCIPWYFANRMISMKKLDVLD
EAVEVIRSTTSEDHRDVDYPDEDDESDTDKEDKEEEAATSSTEEDGQTDPETEEEEDKEESEQPTSKKGS
EPEPEPDQDEEGGDVLPTSGDNNKSEEDRPIDDNGNGESKEEPPTDSNEGETGEVEPTNARKKKRSVKTV
IFKETLPSPKMRNLFSNINSRLVNKKSKRRADPPENLEKREAPTQWWPTPEPIHQSNYSDYICTFTMQAV
CDSVIEEEIKRGKLQMKGCPEPCSYNEWDVELATTVFPSTEKYFDSFIKFEVYVDPAPDFTYARENMARV
HIFYDDIKFDIQKQQKAYEAQNFIAEFGGTVDLFIGFSFFTIFQLIEIALAACVYKCMCWRKGSASCERS
VSLPSLDERDFARAKRDNVDAEISNGKVNGETIEVNILRYDLDRQDKTNEDNANVYDVATE

>MlNaC13 evg114124utrorf_CtenoMnelei
MVVLTVEPWNGATSVSPTTSDEEKGDKSHEMFGFQDLASDYYQVLRQKERPAWCRFVWGIVIIILLIFTG
YMVYQLLADYLQYNSFVESSIIWQKNLTLPAITICSTNPLNYTLLKDQLKRGNDTTLLDQFNQLMEDFVK
SSTIENGHLDISLDKFEDFNAFEHQNGSLVLRFGPHLYNMVFGQYDYVFRGVGYFLDYFEWNKYSQMTEL
GVCLELNDDGKLKQTVGGVNGGLVIDLDANVDHYLFTTMTKGFVVFIRDQDETIMLNRGGYVVSPGVENF
IKLTTTTHTRLGKPHGTCKNVPSKFGKYDHHYESVRECVQRQRMEVMVDMCHCIPWYLADRLFTLNKTDI
LDTYVERLQLHQPARERGRRKRQSEDGTTNTDTENTDTDNTDTDNTDTENTDTENTDTDNTDTENTETEN
TDTENETVEESPTTDSPQPPTTTPWYFVTNEPLHETDYVNLICGFVEMYICDKKLETAEMEGEIEFEPCP
EPCEYHEWNVEIASTLFPPSEEYFDKFLRDQLYLPNTPTYDSIRENMARVHIYYDELKVNQMVQTKAYEP
QNFVAEFGGTVDLFIGFSFFTIFQLIEISVAMCLTKLWQRRRKTRKKEVNTSNSVLANPSVM

>MlNaC14 evg126004_CtenoMnelei
MTEEGDSDALEALRRVLSLLERYDGAGDFFELYEANKATFKQLVSYENRRGSIQLRFSLNLDAVIFGTPH
YFFDGRGKNITDPRKHMQTTKSGMCLEFNNDETFQQSNGGPKGGFSIDLNTNVRDYLPTTSSHGFTVFIR
DPDENVMLNVGGFVISPGTETFVRLTSKSAKRLGPPHGTCKNVPNIYSKRNVKFESIRECWQRQVIQAMI
GKCGCIPIYFAVQMWYRNRTNVLDEFIELIKTVRAHKFYESIYEFTSEEDVARYVSMTNGNASRTKQDNM
ISEIERLKKMFVKSPYFPYHCSLIQERACTLFIDNEITKGKLKLEKCPEPCKYREWDAVISSIPFPPTPT
YFNEFMIGDGVRTFADAKENIARLHVFYDKIRVQETEQIPAYQTHNFIAEFGGTVDLFIGFSFFTLFQLI
EIGIATCVL

>MlNaC15 evg187866_CtenoMnelei
MTGFCLYIVVELLSGYYQYDSYNLEYSRVAQKYTLPAITICNVNPLNYTKLKEKGIYPAYKDWINVVKKR
NSTEDEEKTEPNGDLDDIDIHTQFKMDIKASVIDSLTSFAREPTTYKNFTSFAYTINGECMEMNDNEVLV
QRVNGAIGGLTLVLDTLTGDYLPETETAGFLVTLRMSNETVLSQEFGFLVAPGKQHNINLRTRKVTRLPE
PWGNCEETVDHFDGKGFLTFKECYLNQMLWMYPKLRDLGCGCYPWYFYERYIQDPEDSKYEPNIGWKLIK
YWTEVLPEQFRFNFTDTCKYEESSYKPNSGQIREESGVVSMEECVEICRDDTNCRYFLFTDTTNDILEAN
KCVLYKRGAEMEIIPSKNIVAGLVWGDTQCLNMEKCSVSAGAACVNKIMNELMGAEEEDLDDNGKSKTPP
TCFEPCSYMEHSYDVSVGDFPTRKYWDDYLSKEVGNYDNYDTARLNLLKLVFYTDQMSSIELEQTPSYEI
TSFIAELGGLTDLFIGFSFFTAYQFFEWLVTRVVRKLHTERRTIDSSDNVNVEDANL

>MlNaC16 evg184995_CtenoMnelei

MGRGGELIEGIDNDYLNSIASYKHILPKLIWTVLFSGMLTLGIYCSIELVRDYMDYPSYTEISSQFTTEF
HFPAITICNINRLNRTLMEESTIGNTNTNVYDLYSELMEETSFRSKSKGGVKNPKSKSKGRNKRNEPKIS
YEVINNMNVYRNFKWNIYSTLDKKTLTFAKDNLANLGDTFKVDLTEMGNCLEINDNQALVQKVNGPVGGL
SMILDAQTDHYVRSTESEGFYIVLRMANESVISKEYAFAVSPGKETFIQLETTEVTRLRPPYGSCQDTSD
LFHVKGEEETPGKISNPMTIKECFTGQVLWQFMKEPQCKCYPWYIYSRHIDRTVGKPTRNIDLEAQLYRY
FTSDLPQEKRIEFDNTTCYFQDELSYSGTTLATFESVQRVEGCADRCKTTENCVYFNWADALEQCDLFGE
GAELTADLDYRVTRGNVSCSSSAMEFHECSLTVEAKCNNLMIDLMMSGEDTQKPMECHEPCTYNKTTYTL
SSTNFPSKGIWDSSLAAAFPHYKTFEDAKRNLVKLVFYQELMTRTEEVQTAAYNWRSFVGELGGVLDLFV
GISVLTGFRIIEWLLCLVWKPRKRVGSESKLDVMNMNSSPTAIS

>BoNaC1 Bova011610
MLTSAGSVTPDLSLVRRPLPINCSTAAAMPESKLKIGLTRGLVDGGEIFAQFSLSSSAHYQHLFTYVSAI
CTGEKVLAIYWFSRHDREFVLIHDNATMNQCLAHAGLHEILYIISEGGPVGPKPQLGYNSKAKPDPLLQL
KTSSPSSGPSPDTANLEQSSEEDRDARLKETIPLRRKLKKSPSLEWNYDMEQPASFQEVPSKDEQSAFEV
LPRNERPTYYPYLEHYQPSYPPARPQRPPTKHHNHHHNYNQKHNHMSGSMSESECGQKEAETFLQNSDPP
PKRKKKAKRRANRAALEQRREEIEMETFKEFASTVTADYLAEIFSKASHGRRFIWVSLWLASFVYAWYNI
ALSINVYMAKPTSTKLNFYEAPPEGVIFPTVTLCNFNRFNKSYFEDGQNDQLKRFLRRTRPIWGHVDELD
QREWDTEYEEIKRLNIKRVYTEATHSLEDTVEFCRFSGSPCLSVDNRTTEVPSEAFQKVYTEHGLCYAFN
PNGWLKMSRTGALYGLSLRLHVAQDDYFETQDVAGFKVLLHNSYEPPMIEEYGFALRPGSESYVRIRLQK
YKDVERPLGYCDPLLETSFGGGYTIAGCVMQNRAINTMNKCRCIAFYMDPPPGVSSKNAAICNLFQEWNC
VEPLLEALALGDSEIDGYQIVIPDCPAPCIYTGYTYTISQSEYPSSSVQQRIQEAVQGQKGGDWSIEDIR
DNYLKVHIFFEELSTLEMERYPSYLISNLIGDVGGQLGLLLGMNICSLVQFTDYLMRASFFKGVMGLVRY
MKERKGRGYSKN

>BoNaC2 Bova01453
MISQNQIDIMEKTGINRSNASYEGANSDRGWRPPPTRSVSERYDRDASAALFYPERTGLVRRQKSEQSRK
SDADDRRSRYSQKYECDYDKLPRRSRSNTDTMIRRNQGNQDAPPRAVDYYVDYTCGNTNLSRDLSTTSPH
ARRGKTDMYIEEIPMSILPRKERGATPQSPDRVVTGATSSDKPDKIPEVESVAEFTSNITASFIRIIFEE
GCSIPRRIIWFVMWVACCGYAISNMNAAFTQYFLYDTSSQLSFTQKTGGNLPLPAITICNMNKFRESWLS
VDENVVLMKYEQERVWGKDLVTDEDNEKAKKITVDELEADGRHSIEDMFQEVAFSEENLKKVKRTGADYG
ASFMIHVNQSEYAKTTESAGIKVMLHDYREPPLMKEYGLAIPPGSEAYISTRTLRQKNLGAPYLNSNCTN
KTLQSAEFYTIARCNLECQSEILFEECNCTQAHHPRLGSESRVCSLYDHRMCTEGIMKKIHHGDFSARSE
QCGCIDACSSTSYSTSLSRAEFPSLATAREWINRPGSPYSNITSLKNNYIRLHVYFETLAVETVEKVASY
SMQNLLGDVGGNLGLFIGASVATLAEILDYVARVMYFTVVNKTWRGSGRGQAASK

>BoNaC3 Bova041514
MSSTIFLLIVESIACCRRDGKRKFRTLRSRFHSPEPHYATQDTTSSYDAHTISPTYHETRQNYSEYDRKL
ARPPPTEFNYSRAASSTHRPTRTRKPASSDPMQSRPGYPEFSHGPPLNMAYYRNMTDVESRFLGFNSDKT
LRSTTTRKIPLDISSGCSSSGGDEKVSQPRTGMIVESVGEFTQTMTASFVRFIFDDKSSVFRRLLWFVMW
VICTGYALSNIYNAFNHYLTFETSTKLSYKNAKDGNLSLPAITICNMNKYRKSWLSLPRNQVVKKFEDLR
VWGRKGKKIVITDEDIEKAKTVTMDVLESQGRHSLEDMFHNVKFGEQVLYEVGNTPLGDDWKSHFTERFT
ELGSCYTFNADGRKKVKRTGANFGATFTIHINQNDYADTQESAGIKVMLHHYQEPPLIKEFGLAIPPGSN
AFISTRTLQMYNLGPPYKNSNAPQPADGHRGADRGSPCTMAIPTDSPPALSIKSIDDLKENYILLHVYFE
TLAVEKVQKVPSYSVTNLLGDLGGNLGLFIGASVATLAEVLDYVARVMYAVVKQ

>BoNaC4 Bova05828
MSWVKDFREFANETSAHGVKNIFEGPSKIVKILFLLTWIALTIYSCSVIVSSIVRYVNRPTGTKFEVLVD
SDEMNSEDGENSNRKAIKFPTISICSSNKVRKSYLDLPENELLKEAWNIIDMYDDAELAKLKKRFDDPED
ELVKEGIVDITYQSVLDNGGPKESNVLMCTQRAIQCVNLPAFQKEGYIAMESSITGRCFRINPHGTLYGK
MGDYGSFKLYLWADIGDYSQSMVGVNWFILWLLSDESDEKSALIPILGEDTVQCTRLEDHIAEMSSDAPK
ELEPHLTLLSTVLFAVGQVVGSGIFVSPNEVVKQVGSPGMALVIWVVSGLISLCGALSFSELGTLIRRSG
GEYQYLRAAYGKLAAFLYMWVCGVIRNSMGCAVIALTFSSYLAGLFMSTDHAQFQITKKVGAAVAIVVII

TINSYSTKYGVRLVSLFCILKLAALFVLILLGAWKLCCGETENIAVGFGGTELNPAVWTTAFFAALWSYS
GWNCINVMSGEIKDVKKNLPRALVISQFLLITLFVLANIAYCSILSVADLNADDAIALTVGSKMFGKFGD
GWGRFGEIFFGLSVAVSSFGSLSSSLIVSSRVPFIAAQENMFPKCVALIQTRTGTPIVGLIYIGFASFLC
VWPSNMSQLIGYVGFLSWFWWGLCFLAVPVLRWRMPDCPRQFKVCLLVPIVAFISTVYLVIGPFFINFLP
CLSWLLITLLGVPIYYGYVDDENCCSSDRTKDEPAQGFTVAFHDNTTYGSTMSSGFLMSPGTYYKVDLRR
KKEIRSPPPSGACNASIGITSYGEYSEGACILECKDKALYDACKCVNVVPPLNYIDGIVDDEGNPVGRYE
SCTLKQWVDCGMETYKKWFFDFTNHSRAEDICDCSTPACEEISYEAQVSSSMMSRPWAETTFEKVKGIIG
TAPFSHPELDIVYDSAQDVLDNVMTLEVLFTSMQTSEIREVVTYTSANLLGDIGGVLGLFLGASIFTVLE
FCQFLFFSIAKHCCNLGGKKKKGGAQRLDDENGDEKMAMLTRHIFPCSKCDFEAISKNGLKEHLESDHPP
LNNDLCAKDDGVIKKPRNSMTKANERLKTLSNDSSFGTGGAYDDPQDDDYFPGEGSDAEASENAAQAKRR
MKSKRIRIRPPQNCTHNGCEFTTIKKVQMNRHRKDVHGDIIPRKKPISKQEHIQGKKSNRPRVREKKFSC
DCPVVIIISQLLMTVVVTDILKCFRLVSYPEVSMSSIRKVMPAPYNSSVQPGARSREHTVTQHTNVHGSQ
EVLRSLCDDWGEDNMSKAVDSRQFRTIDIDAYDEEAYQDIKSADVTGAQE

>BoNaC5 Bova000616
MVLHDTDESDEKSALIPILGEDTVQCTRLEDHIAEMSSDAPKELEPHLTLLSTVLFAVGQVVGSGIFVSP
NEVVKQVGSPGMALVIWVVSGLISLCGALSFSELGTLIRRSGGEYQYLRAAYGKLAAFLYMWVCGVIRNS
MGCAVIALTFSSYLAGLFMSTDHAQFQITKKVGAAVAIVVIITINSYSTKCGVRLVSLFCILKLAALFVL
ILLGVWKLCCGETENIAVGFGGTELNPAVWTTAFFAALWSYSGWNCINVMSGEIKDVKKNLPRALVISQF
LLITLFVLANLALTVGSKMFGKFGDGWGRFGEIFFGLSVAVSSFGSLSSSLIVSSRVPFIAAQENMFPKC
VALIQTRTGTPIVGLIYIGFASFLCVWPSNMSQLIGYVGFLSWFWWGLCFLAVPVLRWRMPDCPRQFKVC
LLVPIVAFISTVYLVIGPFFINFLPCLSWLLITLLGVPIYYGYVDDENCCSSDRTKDEPAQGFTVAFHDN
TTYGSTMSSGFLMSPGTYYKVDLRRKKEIRSPPPSGACNASIGITSYGEYSEGACILECKDKALYDACKC
VNVVPPLNYIDGIVDEEGNPVGRYESCTLKQWVDCGMETYKKWFFDFTNHSRAEDICDCSTPACEEISYE
AQVSSSMMSRPWAETTFEKVKGIIGTAPFSHPELDIVYDSAQDVLDNVMTLEVLFTSMQTSEIREVVTYT
SANLLGDIGGVLGLFLGASIFTVLEFCQFLFFSIAKHCCNLGGKKKKGGAQRLDDENGDEKMAIVKPYWN
DLPKAVKEASNVNDFKASLKKKKKKFNNFLVENPFIAKKKDKHLPLATFKRLTRHIFPCSKCDFEAISKN
GLKEHLESDHPPLNNDLCAKDDGVIKKPRNSMTKANERLKTLSNDSSFGTGGAYDDPQDDDYFPGEGSDG
EASENAAQAKRRMKSKRIRIRPPQNCTHNGCEFTTIKKVEQNMAAVQSLKWIGACLFYAITSFVIVAVNK
SVLTIYKFPSVQFVSFSQLLMTVVVTDILKCFGLVSYPEVSMSSIRKVMPLPIIHLFNLVLGLGSTQSLS
IPMFTVLRRFCVLFVMIGEVWLLGKRFNKMTCATVFAMLFGAIVASLGDLAFDSYAYIMVFANNFVTAAY
SFNILCTSICGPLTFNVVGVLKSAVMTYVGMIFGGDYIFSWLNFTGITISAVGAVVYSAATFIAANKTSK
EMVAKVHRGDAASIKA

>BoNaC6 Bova07806
MGRNGFDRSKKWWITSLSLLHPLNCLHATDPHLISPEPNQDSSWKCAVESIAEFSQETTAHGVKFFFQGK
KLSRVAFVIAWLAAIVYTFYTCYRGIVDYQSRPTGTKFEIITEDYVNVTFPTITLCNVNPVENDYIESVE
GLGSKELIPEEVALWRPLSHSGIVALKDVFHDATGSYWMYVMEYDANYRDLFNFIDKNGSFTSAAASHIT
RQVVEVVKYLLAAGVDHRDIKDENILINPTDNSVKLLDFGSASSYSAEQGAYLSYQGTDVYLPPEYYVTR
QYEAGPATVWAIGCLVHCLVAGDSPFDDKSEIANYVRLQWLDEGDDLAKDFVDQCMQRDPAKRPSVHELL
SHPWFNEPETNPTNLPVMKMTYADILKNGGPADDTILACKIGEDDCSSHPLLKDHKVFSREVTNAGNCFK
INAEGLLKVSIPGSLGAMTLLINTRSTQYRSGNLAEPSEGYKISFHDHLSDGQVQNRGFVLSPGFMYTIQ
LSNKLTKMLPIPYGLEDCVTSSSDPKYRRNVCQNECIAKYVQKKCGCVTYPNPARSMNIRSQECGHCLGS
CEFETYDHTVSSSVISNKFIEMMMSVAPSTQEGVDEEAAEKSPTCSFAFRKICEKLLANTLERLKTNRVK
ENILFLKIVFPVIERQTINQVVAYGLSNLLGDIGGVMGLFLGASVFTILEFVDFFVRAVFAYGKHLSAKP
SGWTMCNIGADIVNLNNDIVNMNNDVVNMNNDVVNCDIVNMNNDSLWDRDQR

>BoNaC7 Bova045921
MDLIAAGNGGSPPLLHPLNCLHATDPHLISPEPNQDSSWKCAVESIAEFSQETTAHGVKFFFQGKKLSRI
AFVIAWLAAIVYTFYTCYRGIVDYQSRPTGTKFEIITEDYVNVTFPTITLCNVNPVENDYIESVEGLGSK
ELIPEEVALWRPLSHSGIVALKDVFHDATGSYWMYVMEYDANYRDLFNFIDKNGSFTSAAASHITRQVVE
VVKYLLAAGVDHRDIKDENILINPTDNSVKLLDFGSASSYSAEQGAYLSYQGTDVYLPPEYYVTRQYEAG

PATVWAIGCLVHCLVAGDSPFDDKSEIANYVRLQWLDEGDDLAKDFVDQCMQRDPAKRPSVHELLSHPWF
NEPETNPTNLPVMKMTYADILKNGGPADDAILACKIGEDDCSSHPLLKDHKVFSREVTNAGNCFKINAEG
LLKVSIPGSLGAMTLLINTRSTQYRSGNLAEPSEGYKISFHDHLSDGQVQNRGFVLSPGFMYTIQLSNKL
TKMLPIPYGLEDCVTSSSDPKYRRNVCQNECIAKYVQKKCGCVTYPNPARSMNIRSEECGHCLGSCEFET
YDHTVSSSVISNKFIELMMSVAASTQEGVDEEAAEKSPTCSFAFRKICEKLLANTLERLKSNRVKENILF
LKIVFPVIERQTINQVVAYGLSNLLGDIGGVMGLFLGASVFTILEFVDFFVRAVFAYGKHLSAKPGATSK
QEISSGV

>BoNaC8 Bova011512
MTHNMTRAKEFTDSAYRSGNGFMKRVPAPPIPSRNAAFPIYDNLVQSRPAEQIPPGSPELPPLHHQNNSN
LHLLHMSVLFALVRKSWRSTGSVDMTGSLSLFHDNATMNQCLAHAGLHEILYIISEGGPVGPKPQLGYNS
KAKPDPLLQLKTSSPSSGPSPDTANLEQSSEEDRDARMKETIPLRRKLKKSPSLEWNYDMEQPASFQEVP
SKDEQSAFEVPLETSDRPTIHTWSTTSPHTLQPDPRDPQPNTTTTTIITTRSTTTCSMSESECGQKEAET
FLQNSDPPPKRKKKAKRRANRAALEQRREEIEMETFKEFASTVTADYLAEIFSKASHGRRFIWVSLWLAS
FVYAWYNIALSINVYMAKPTSTKLNFYEAPPEGVIFPTVTLCNFNRFNKSYFEDGQNDQLKRFLRRTRPI
WGHVDELDQREWDTEYEEIKTLNLKQLYERGAHTPTQTVEFCRFSGSPCLSVDNRTTEVPSEAFQKVYTE
HGLCYAFNPNGWLKMSRTGALYGLSLRLHVAQDDYFETQDVAGFKVLLHNSYEPPMIEEYGFALRPGSES
YVRIRLQKYKDVERPLGYCDPLLETSFGGGYTIAGCVMQNRAINTMNKCRCIAFYMDPPPGVSSKNAAIC
NLFQEWNCVEPLLEALALGDSEIDGYQIVIPDCPAPCIYTGYTYTISQSEYLAAACSRGYRKPCRDRREE
IGALRISGDVGGQLGLLLGMNICSLVQFTDYLMRASFFKGVMGLVRYMKERKGRGYSKN

>BoNaC9 Bova017512
MSSTIFLLIVESIACCRRDGKRKFRTLRSRLHSPRAPLCNSRYNEQLRCTHHLPTYHETRQNYSEYDRKL
SRPPPTEFNYSRAASSTHRPTRTRKPASSDPMQSRPGYPEFSLGPPLNMAYYRNMADAESRFLGFNSDKT
TEIQYDIPRYHNPHHDKLTREVRERKLRSQHDTPATTRKIPLDISSDCSSSGGMTKSHNPELGSCYTFNA
DGRKKVKRTGANFGATFTIHINQNDYADTQESAGIKVMLHHYQEPPLIKEFGLAIPPGSNAFISTRTLQM
YNLGPPYKNSNCTSTPQTDYPSVASAVNLVEKPTEAATEEPTEGVPCTMDIPTDSPPALSIKSIDDLKEN
YILLHVYFETLAVEKVQKVPSYSVTNLLGDLGGNLGLFIGASVATLAEVLDYVARVMYAVVKQ

>BoNaC10 Bova009214
MVVLHVQPWPTDDHQTSSSPPPTLGVPPVRPATTHPMKEIEFFGFQDLASDYYQVMRVPGRPRWCRALWF
IVVLFLLGFTFYMVYRLLHAYFQYSSYNKSSVTWESNVTLPAITICNINPFNYTNFKEEANGTDLVEDFE
TIMETLIFYNEDGDAWEEINDTIWEGFLEYEENNGALSLRFRDWLIHTVFGYYDYEFQGNGYFIDYDDYF
KFSQMTELGNCFELNDDGALKQSSGGARGGIRIDLDAKHADYLFSTPTQGFMVYIRDQDETLMLDHGGYV
VSPGTEVFLKLTAKSVTRLGEPHGTCENVPSRLAKYGPHYETVRECIQRQKLDVLMERCGCVPWFLADRL
STLKKTEILDEYIEKILDGMGTEAQVWGRRRRGAPSNPREDFLHRHKINHRGIKERFRDTRSLVEDPQHE
IFDNPGFDEFLDKFEKSDPDILDREKRNHNGGSTATPRPPSTSPYYKTEQYGPHICGYVEQYSCQMWLDT
LIRDGNFTLDPCPEPCSYHEWQVETSSTKFPPTEEYFERFVKDRVYLYSGEPTYEYIQNNMARLHIYYDE
LKVANIDQTKAYEAQNFVAEFGGVVDLFIGFSFFTCSS

>BoNaC11 Bova06865
MGIERDVEVIKNKAAAEAEKEERRENLERDMFYTTGDLTSDYFQSITRSKHPIWCRITWGAIIIGCLAFT
IYMVYNLLHGYLQFKSYNQDSVVWQSSVPLPALTICNVNVVNYTAWSQNTASAGSLEEELNWFILDVTTK
MKTSEKGTTVTLTDEEMERTNKLDQVTTEDGHVLSILSDLHSKFNKFFLGDGESHPFMEFSYRGVKGGFT
IDLHAREEEYLYATDAAGFMLYLRNPHEIVVADIGGIYLSPGEEHFISLEENVITRQKSPYGTCDDQKSD
FNTSNSQTVRECIQALYLRETIKRCGCLPWYLDDTLKTYFEGDEAAFEIWAARNNVPDGATLKGIEALSC
SFVQQTVCDLNVIDALARMEISETCREPCNYKEWEYSLSSGIFPPTYGYFMDFLEPYVNRDGYDNYASDT
SGDTDTSGDTDTSGDTDTSNSHDTIGQYFNYARKNFVRIHVYYDDVKLTKVVQTKAYELSNFIAEFGGAT
DLLMGLSFFTVFQLFEILVVYIIFRCCPRKKGNSGDDASNEV

>BoNaC12 Bova04282
LVDVQNVDSVDNNSSYPETHSERNIDKISKAGIRAIVQEEREKMEDFEVQDAVTEVKKKGLSCDGVYLVT

GGLGSLGIELATWLVSNGCTVLLIGRNAPSPSARVALDSLRKVGRTLVKVELGDVRDRDFVENLFLKNKI
GQSAYGFANSVVDGICRRRIEDNLPALSLQWGLIGVGMGTNIKQVCGSAPMSQITFHSCLMDVLDLSLSG
VVLLYVPYQENGDKPGGSSTALLSKVSKVLGVTDLVSHGDTLLSRFGVDSLMAVELSALLTRDAGAHFTP
DQIRELSFSGLHKLCSINEDQDDFAEETSTHGLMFAFRSRGPVKIISMVVCTVFFFVTVYNVALNIQKYR
EDPRGTLITQKIKISDPHAPTVFLCPSSMIDKTLLAPLLQEFYNKNEFINRNQINANRNILLRGGHYGAI
NFVTMLDPDHSLKNSLEYGLEVFIKDQGSIINRFDQGDILTSARVHTLKVAVEEVKSMKQRQDEIVCFDT
EDPGMYYPRAFYGSPRMCDKSCYAAHAVTFLNKTQGCIDVPNFDSEDMPPCTIRDLKDYMVYSSDIGITN
TKKELIDKYLEEAVPSGCVPPCILKSYSYYVHSNLIDDKLYTFLSTRLSTPVDKLKKTLIIIKILFPDIK
SEVTEIRTYPLSNLLGDVGGVFGLFLGFSVFTFLEYIYFAVFYLLKKLCGQKITGEWLVD

>BoNaC13 Bova05225
MAEEGMVMENKTAGGGHNGGDDDTRPVLDRDVFYRKEDLTSDYFQSITRSRHPLWCRLIWGIIIVASLAF
TVYMVYNLLDGYLEFNSFNQDSMVWQNGVALPALTICNVNVVNFTAWSEQSWSAEDLESELNWFIKDVTM
EMKTSSDDGSFIKLSEEDIKRAKKLDKVTLENGETVSIYDLHSKFEKYFLGNGADHPYMEFAYRYKDPPS
SDNYINTKLGQCLVLNDDQVMVQKLGGVLGGLTIDLDTREFEYLYATESAGFMLYLRNPHEIVISDIGGI
YLAPGFKHFISLERNDITRLQTPYGTCEDQKSVFNTSRYQTMRECIQSLYIEETVKTCGCIPWYVTDTLE
TYLSEDGDAFKTWLRTQGLPEETSMDEIETFACSFVDQTICDLNVIDILSNMDISKVCRESCMYKEWDYS
LNFGQFPPFMGYFSTFISPYVNRDGYEKSGPVGVGFDMDTDPNTDTDTETDPNTDTDTETDPSTDTVTDT
ETDTALDDQAFYTYAQKNFVRIHVYYDDVKLTKVVQTKAYELSNFIAEFGGATDLLMGLSFFTVFQLFEI
LVVYVIFRCCPKRK

>BoNaC14 Bova01274
MERDRVANIYLPSTEERPKKIKEQLKESSSLSVEEASVGLCSHALVICACIAILVTMPFSLLLCIKVIQE
YERAVIFRLGRIAKGGARGPGLCFHIPCLDEITVVDMRTLSFAVPPQEILTKDSVTVSVDAVVYFRISSA
VASVCNVEGVQKSTRLLAQTTLRNILGTKSLSDLLAEREAISQDMQNTLDSATAPWGVVVERVEIKDVRL
PANLQRAMAAEAEAFREAKAKEIAADGERNASHALRDASDTMMQSKCAIQLRYLQTLTTIASEHNSTVVF
PVPVDLLGTILDRGDPCEGRCGGNLVAGCRCQCCTAKLDVKARPLSEEKRNDVDQEGHLTLVWSLLGAVM
LGLALYVTILLVADYRSFPSYTKVSSESSHQFNFPATDANQMSYLGSGLYGGGLYGGGLYGGGRYGGGRY
GGLFNLGWTLRRIFNLGWTLRRILGHKFAKEIVEPGTMAELIQVDYTEMGNCLEINDNEILVQKVNGPVG
GLSFVLDTKVSHYTSDTTSQGFFIMLRMPGEKVVNKEYAFRVTPGHEVFVRLNPIQVSRLGEPWGTCLDA
PDIFTEYSLDEDDEMDVMSVKECFSSQVLWSYMKDEVCRCFPWYIYERYINNNGQKNDELYNALIEYWSN
GLPEDKRIQFGTTEVVSDQYSWAGTVIESSTQPSVQDCARQCESNDECSKFMFTDARFLPSLESGGSCEL
FEASATLSDSLGYKVFRGTVDGTGEVHECSISKENICGNLVMETLMSGDLSDLPPSCSEPCSYNRTEYSL
SMSRFPAQRLWEATLSDKYPQYSTLDAAQRNLIKLVFFQDVMRMTTETQTASYEIQNFIAELGGTVDLFI
GISFYTIYKLIEWVMYYIYRRHSKHKGASGPQSQAANKI

>AqNaC1 Aqu2.1.34376_001_PorifAmpQue
MPSKDKGENDPQENTGVRNRKEKSCQNPQFTKWAESSTIHGVDHIFLGKSKVRRVVWAVILLLAIGGCLY
GIIDRSIYFASKPTATTVTADINEDGIPFPAVTICNLSPISRQYADQHNLTSLLSYILFTDSNTHSKGFI
SSNCQANLEQITDTTITLKDVFRDGARNSSFILACHYGSARNKMDCANMTLRTTLTPRGLCYTFNGDPAS
PPLLVRSVGERFGLRMIFNISQSDYTHSINGDAGIRVSVHTRDEKPDPLLKGISVPPRSHASIALYPIRS
ISKPEITRCAPTDTQLSYFPGLSYTTSGCQANEHFERSQAQCGCVDVAESATNDCTVEDICCLYDEGTST
DTLNSTCLPSCNNMIFSSSVSYSQYPSDVTVSLLTSFQSQSAESIDDNILALNIYFGSLHTIVTSTYYTY
LWSGLLADIGGQLALFVGASVISFMELVLLCFDETKCSGVFIRKKIKKKHEEHELEERDDKESLNGGKIE
DKQANTNV

>AqNaC2 Aqu2.1.09805_001_PorifAmpQue
MAEDSHSKSSEEKTVKCHLHDRYLKEFLDDNTIGGINHIFRGRSKVRRLLWALIFIGSIVACITLISISF
QTFLEKPTASTITVITQDDEGVSFPSVTICNLNLERNESDMVADTGYLLMNHIFNPDENFHLTGLNSSFL
LNSCNAISDSFPASFRNTTLWNSQHPQETLDKLIHFCGFVSGINSAVIPCKDAFKPVLTSAGICYTFNGS
NNRIHSTGVRYGLKLILNIQQEERPSFNGKSGVKLIVHDGRDIARPNLYGIDVAPAHAVDVGVRRKASKD
ETNEADCIDSKELPFFSEYRYSQFACRQNAIVENLATSCDCSIHPDRPSSGPYSSTPHCTFDKGCCVLEQ

YQTFNPELACPLPCYFPYYEHTASYSSFPNGRYLNYLVEETNMSVDYIKDNFLSINVFVDDLQLTTTITK
YTFGVAELLGEIGGQMGLFLGISIISIVEVVVLFLDELKRLFCTKKMREKMQDIENAIELPEIEGTDVEE
DIDNKV

>AqNaC3 Aqu2.1.34363_001_PorifAmpQue
MNGKDKAKEPLKPADSERDEKGKGTCHLSDPYLSDFIDDNTIAGINKIFRGKSNLRRLIWAIIFIGSLIV
CTVMLSFSIKRFIDKPTASTITIVSNTEQGIAFPAVTFCNLNLERNSSNFLLRSTYQLMNYLYNADENFH
LSGLNDSYVLQLCDNVVRSSSEDILNATIWNIQNPSKTIDELIHYCGFIEGANSEVKPCKDAFKPILTSA
GICFTFNGSDNRIHSTGIRYGLKLILNIQQKERPSFNGKSGVKLVIHNGRDIARPNLYGISVPPGHAIDV
GVRKKAVEDDTNEAQCIHGMNLPFYPSDKFDYSQLACRENALAENIAHSSKCSCAIDRPSTGPYASTPNC
TFSKACCLLKEHYEFNAEEADCPSPCHFEYYEYTSSYSTFPNGLYLDTLVNKTNMSVNEIRENFLSVNVF
VDDLHTTTTITQYTYGVEALLGEVGGQLGLFIGVSIISFFEVLILCIDELKRLCCRGSVKRTMKKLEKMI
RLPEIDSGETDKSNEIELNSVEIISCDEERSIKDLSPCDEDNKAVLLPIEDKSNEVGLQSVKTTEV

>AqNaC4 Aqu2.1.26218_001_PorifAmpQue
MPTLVRNAHVWTDQYFSDFIDTTTINGVFQIFRGRSKIRQIFWGLLFIGSFIGCIVTFGYSFRNFAQKPT
ASTIKVITQAQTGLAFPAVTICNLNIYQNPNYSNVLSSEMYALIQYLFETDDIFNEFNITSECKDLIDNA
SEDYGKESLYDMLLPKDYSSNLIYDCTFRDDALGDAMSCKDQFYPVLTPGGICYTFNGVRSEMIAPVIKS
IGIKYGLKLVLNIEQETHPTFDGRTGVKVIIHERNDIPRPNLYGINVSPGQNIDIGVSRSFFIDETDQDK
CNNDIEGNFPFLPNIVYSQFACRINQLYERLSQQRNCGCLPIPYRPESGPYTNTPNCTLGNLCCLLREFP
IADASSTCQLPCNYSVYEYRDSYSSFPNGRALTEIARKVNMSKTDVKENFLSVNVFFEALHTTESITQYT
YGAVDLLGELGGNMGLFLGISIISIMEVIMLILDEIKHLCPKKVKKKFDNIDDKLRNYIPDIAPSQTDTP
NALEAGTEEVHVEDPSPSEADIKSNAEAIEES

>AqNaC5 Aqu2.1.09804_001_PorifAmpQue
MNKGKAGEPLNSLKAADSENDEKGKGRCHLNDPYLSDFIDDNTIAGINKIFRGKSNLRRLTWAIIFIGSL
IVCTVMLSFSIKRFIDKPTASTITIVSNTERGIPFPAVTFCNLNLERNSSNFLLRSTYQLMNYLYNADEN
FHLSGLNDSYVLQLCDNVVRSSSEDILNATIWNIQNPSKTIDELIHYCGFIEGPNSEVKPCKDAFKPILT
SAGICFTFNGSDNRIHSTGIRYGLKLILNIQQEKRPSFNGKSGVKLVIHNGRDIARPNLYGISVPPGHAI
DVGVRKMAVEDDTNEAQCIHEMNLPFFPSDKYDYSQLACRENAIAENIAQSSKCNCVIGRPSTGPYASTP
NCTFSKACCLLKEHYEFNPEEADCPSPCHFEYYEQTSSYSSFPNGFYLDTLVNKTNMSVNEIRENFLSVN
VFVGDLHTTTTITQYTYGIEALLGEIGGQLGLFIGVSIITFFEVLILCIDELKRLCCRGSVKRRMKKLEK
MVRLPEIDSGETDKSNEIELNSVKIIQCDKERSIKDLSPCDEDNKAVLLPIEDKSNEVGLQSIKTTEV

>AqNaC6 Aqu2.1.26214_001_PorifAmpQue
MKNAGRFSWSDKYFSDFVETTTINGVIHVFRGRSKIRQIMWGLLLISSFIAVCVVIGFNIKEYVNKPTAS
TILVSPSVKNGLPFPAVTICNLNVYTPTGEDKEPDFSSVIHSLFNSQDLVNQTSLFDECSEVINSSDTSD
AFDCEVWDLLLPDDKRFIYQCSFSHDADSEIVSCRDMFYPVLTPAGICYTFNGIRSKMPVPMAKDIGVRH
GLNLILNIEQDSHPTFHGLTGVKVIVHDRNDISRPNLYGISVAPGQNVAISVERKVYIDKTKERDCTNDE
RELGFFPSTVYSQFACKENALYQHLADESVCGCVPNPYRPSTGPYINTPNCTLHSLCCLLQEYFDYRVSS
ATCPLPCQFSMYDYKSSYSSFPNGHALKSIAKSLNLSQDAVKNNFLSVQVYLESLETHEYVTKYSKTLTG
LFGDIGGLIGLFLGMSIISIIEVLVLILDELKKLLCIKKFRKKVKKVDDMLTHFLPDVK

>AqNaC7 Aqu2.1.26220_001_PorifAmpQue
MQPAKGYTLTDQYFHDFVETTRISGIKHIFRGRSKIRRIMWALFFISSFVGCTLVVGKNIARYIEKPTAS
SIKVIPHDNGMRFPSVTICNINIYKDPNVSAVSKETYSLIYYLFNSDTKEYNSTQECIDNATEYYKKHDI
WSSLLPKENNFIHDCSFSYETGVISCKDMFYPVLTPAGICYTFNDFKMNEMLIPNITSGMKYGLKLVLNI
EEERYPAFEGKTGAQIIVHERNDIPRPNLAGISVPPGQNIDIGFIKAIKNDKTDSRDCINDGEKKLDFLP
DVVYSKFACQENELYERLASNCNCTINPYGLDVTNTSNCSLSNLCCMRQQYSKYNRNSSCKSPCKYTFYN
LKNSYSSFPRGRTLTEISKTVKMNKSAIRDNFLSVHVFLLDLETKETYSHNSFDISELLGELGGTMGLFL
GINILAIVEVIILILDEIKKYLCPKKCKQKLNKIENCIPECALSRGRNDTLLTDPVSTPEKENHETPYTT
AVDIDN

>AqNaC8 Aqu2.1.34361_001_PorifAmpQue
MAAEKKDVEMASEKEDMQMMETKKKKGRGCHLNDPYLKEFLDDNTIAGMNHIFKGQSKIKRLVWALIFIG
SMIACITLISISFRRFINKPTSSTITVVTENTKSGVEFPAVTICNHNLEYNLSDYIIRNTYLLMNYLFNA
DENFHLTGSNSSSVIKQCQALVGDAPSEILNATIWNIQNPSKAIDELIHYCGFIEGVNGEVEPCKDAFKP
VLTSAGICFTLNGSDHGIHSTGIRYGLKLVLNVQQEKRPSFNGKSGIKLVIHNGGDIARPNLYGISVPPG
RAIDVGVRRKATKDETSEAGCIDDMDLPFFPKDKFDYSQFACRENAIMERIAKRSSCNCVIHPDRPSTGA
YSSTPNCTMSNACCLLHEYNTFHPESSSEECPLPCYFSYYEWTASYSSFPNGRYLDRLVNTLNKSADYIK
NNFLSVNVFLDDIQLTTTITQYTYGPEALLGEIGGQLGLFIGVSIITFFEVLVLCVDELKRLCFKLQIIP
EEKINNLESRITLPEVEEDQTEN

>AqNaC9 Aqu2.1.25935_001_PorifAmpQue
MTKKEFKWSDQYFNDFVETTTINGVFHIFRGRSKTRRLLWGLLFLISFVSCTIVLGFSFKRYSEKPTVSA
INVISEENGMPFPSVTVCNRNFYKYPNISNETDTLIHHLFHSSGFLQNINRTQECKIDEDTPDFKLQDLL
IPKENNFIYYCALSYQAESEVMLCKDMFFPTLTPAGICYTFNGVRSKTRALNMTTTGKRYGLRLILNIEQ
ETHLAFDGVAGVQVIVHESNDIPRPNLHGIGVPPGQNVDIGVKRAISSDETDQDACIENNEKDLPFLPGV
VYSQYACRQNELYESLADKNLCNCIIDPFELDNATCFLHDLCCLQQQFTEHDRNSSCRPPCKYTYFDIIN
SYSSFPEGHALTEIKNSINTSDDYVKKNFLSVSVFLQALETRETTTRYSYGVVELLGELGGNLGLFLGIS
IISVMELLVLVVDELKKCVCPKKVKQKFKKFDEKLKCIPDCVEQEEKNEIQALTNF

>AqNaC10 Aqu2.1.36244_001_PorifAmpQue
MDEGNDPVNGEKSKCKNCTCTCTDRYLREFLEDNTIAGISHVFKGQSKVRRSFWALIFIAAIISCIALIS
LSIQIFLNKPTASTINVITLNGVPFPSVTICNINFEKNEALPLLSRTYNLMNQLFNADESFHSNSMNASS
VISNCRNTVPRNNSDATFEATIWNIQRPARSKRRLIHYCGFVTGVNSNVSNCKSLFQPVLTPAGICFNYN
GSSNTIHSTGTRYGMKLVLNIEQDLRPSYNGKAGVILSVHDGKDVARPNVNGINVAPGQAIDVGVNLKEY
IDETKEANCTAGQDLVFFEDYDYSQYACAQDALIKQIAKPNVCNCTLLPRRPSNGEYDHTPNCTFPTSCC
LLNEYKTINTEEQCPLPCRYRYYDYTTSYASYPNSFILEDLMRDENVTEDYLRKNYLSINIYINDLRYSV
VTTSYTFGVAGLLGDIGGQLGLFIGVSIITFFEVLILCLDELKRICCPDFVINKCKNMKKKGESSDIVAG
ERETRRQIQRKAWSNDSL

>AqNaC11 Aqu2.1.26219_001_PorifAmpQue
MTKKGFKWSDQYFNDFVETTTINGVFHIFRGRTKTRRLLWGLLFFISFVSCTIVLGFSFKRYSEKPTVSA
INVILGENGIPFPSVTVCNQNFYKDLNVSNETNALIHHLFHSGGFLHDINRTQQCKIDEDRHDFELQDLL
IPKENNFIHYCAFSHQAESEIMLCKDKFFPTLTPAGICYTFNGVRSKTRAPKMTTTGKRYGLRLILNIEQ
ETHPVFDGVAGVQVIVHESNDIPRPNLHGVGVSPGQNVDIGVKRAIMFDETDQDECIKDNGKDLPFLPGV
VYSQYACRQNELYESLADKNLCNCIIDPFELGNATCSSHDLCCLQQQFTEHDRNSSCRPPCKYTYYDIMN
SYSSFPEGHALTEIKNSINASEDYIKKNFLSASVFLQALETRETTTRYSYGVVELLGELGGNLGLFLGIS
IISVMELLVLVIDEVKKCVCPKKVKQKFEKFDDKLKCIPDCVEQEEKDENTSGPSAELNKIA

>AqNaC12 Aqu2.1.34365_001_PorifAmpQue
MNPADETDAEKVSGSRCKSCQLKDSYFNDLDKTTIAGLNHVFMKDSSKIRRLIWALFFIGCILGCLVLIG
LSINRFVDKPTASTITVVSNDETGIFFPAVTICNLNLKKNDSDVLLNTTYQVMNFLYNSDESFQFTGSNT
MSLGNTCTAPLSNSIQNATIWDIVNPDRAVDELIHYCGFLHGADSDVVMCEDLFEPVLTSAGICYTFNGT
NKLANSTGIRYGLKLILNIQQKERPSFNGKSGVKLVIHDGRDISRPNLYGISVPPGHAIDVGVHKMATQD
ETSQANCIKSMNLPFFPSDKFDYSQFACRANAVAENIARRSKCNCVIQPDRPPGLYASTPNCTFGKACCL
LQEHYKFNPEEINCPLPCHFQYYEHTASYSSFPNGQYLQRLMEASNMSAEDIKDNFLSINVFIDDFQVTT
TTTQYTYGIEALLGEIGGLLGLFIGVSIITFFELLVLCVDELKRLCCSQAIIKRMKRIEETALVPVVESA
EGSLSNEEDAPEEVTSQPEAESNKTSPSSRDANSDSNEGKCIKIEEIKL

>AqNaC13 Aqu2.1.13200_001_PorifAmpQue
MCSDGAGHAYDKVSKYFKKKITIAGLSHVFPERTKKDDRTRSGLTNPEKIIMVIWALFFTGCVLGCLVLI
GLSTKRFVDKPTASTITVVSHDKTGLPFPAVTICNLNLKKNDSDFLLNTTYQAIISLYNADKSSQFTSNK

KIDHLLNTCTASLSNSIQNATIWDIVSPEMAVNEFIHYCGFLHGADSDVVMCKDLFEPVLTSAGICFTFN
GTNKLANSTGRRYGLKLVLNIQQEERPSFSGKLGVKLVIHDGKDIARPSLYGISVPPGFAVDVGVRKMAT
RDETSEAKCIDDMNLPFFPSDKFQYSQFACRENAVAENIARGSKCNCVIQPDRSPGLYASTPNCTFSKAC
CLLQEHYRFHPEEIDCPLPCHFEYYEHTASYSSFPNGQYLQLLMEELNMSAEYVKDNFLSIDVFFDDFQV
TTTTTKYTYGIEALLGEIGGLLGLFIGVNIINFFELLVLSGDGLGMLCRRAGRSCRRALEKIKKRKNERK
EEPMKMDTQPGSSRSGNGHA

>AqNaC14 Aqu2.1.20433_001_PorifAmpQue
MAGNDPQNPKAPVADKTSRSPTVTSGPPSPETNRPPALEPPRSGVDREDTSTVDITPVYDKVSEYVREKI
TIAGLGHVFPNPEKKKGVHSLSTCEKTMMVVWAVFFTGCVLGCLVLIGLSFKRFVDKPTASTITVVSHDK
AGLPFPAVTICNLNLKKNDSDFILNTTYQAIISLYNADKSSQFTSNKKIDHLLNTCTASLSNSIQNATIW
DIVSPEMAVNEFIHYCGFLHGADSDVVMCKDLFEPVLTSAGICFTFNGTNKLANSTGRRYGLKLVLNIQQ
EERPSFSGKLGVKLVIHDGKDIARPSLYGISVPPGFAVDVGVRKMATRDETSEAKCIDNMNLPFFPSDKF
QYSQFACRENAVAENIARGSKCNCVIQPDRPPGLYASTPNCTFSKACCLLQEHYTFHPEEIDCPLPCHFE
YYEHTASYSSFPHGQYLQVLMEELNMSAEYVKNNFLSIDVFFDDFQVTTTTTKYTYGIETLLGEIGGLLG
LFIGVNIINFFELLVLCMDATKLCNKCFKKEKPNKRTNPVELDNVLVTKGSRNSTKPMNTVVGDTVTVN
